# Adverse events associated with tirzepatide: a focus on subgroup-specific differences

**DOI:** 10.1186/s40360-026-01105-3

**Published:** 2026-02-26

**Authors:** Zhongming Yu, Yiming Qi, Qian Gan, Guodong Wu, Xinhao Zhang, Cheng Jiang, Jing Li

**Affiliations:** 1 Zhejiang Academy of Traditional Chinese Medicine, Tongde Hospital of Zhejiang Province, Gucui Road NO. 234, Hangzhou, Zhejiang 310012 China; 2 Zhejiang Provincial Key Laboratory of Disease-Syndrome Integrated Cancer Prevention and Treatment, Tongde Hospital of Zhejiang Province Affiliated to Zhejiang Chinese Medical University, Hangzhou, Zhejiang 310012 China; 3 Fourth Affiliated Hospital of Harbin Medical University, Harbin, Heilongjiang 150081 China; 4 Fourth Military Medical University, Xi’an, Shanxi 710032 China

**Keywords:** Adverse event, Tirzepatide, Subgroup, General disorders and administration site conditions, Gastrointestinal disorders

## Abstract

**Background:**

Tirzepatide is anticipated to play a pivotal role in managing overweight/obesity and/or type 2 diabetes. Although prior studies have employed spontaneous reporting databases for large-scale safety monitoring of tirzepatide focusing on novel adverse events, current understanding of potential disparities of the adverse events among subgroups remains unexplored.

**Objective:**

This study aimed to evaluate the differences of adverse events across subgroups related to tirzepatide.

**Research design and methods:**

Using the data from the United States Food and Drug Administration Adverse Event Reporting System (FAERS) and Japanese Adverse Drug Event Report (JADER) databases, the differences in adverse events across specific subgroups concerning reporter type, sex, age, dose, indication, report year, onset time, and outcome were investigated using the ROR algorithm and Chi-Square Test/Fisher’s exact test. To further verify the differences, subgroup-based stratified analyses and sensitivity analyses were conducted.

**Results:**

The results showed that the adverse events related to tirzepatide showed significant difference across subgroups. Notably, males were significantly more inclined to report “gastrointestinal disorders”. Within indication-based subgroups, tirzepatide used in patients with diabetes mellitus was significantly more frequently associated with “gastrointestinal disorders” compared with patients in weight control purpose.

**Conclusion:**

This study provides contribution for identification potential difference across subgroups, which can guide future research and more targeted monitoring strategies for tirzepatide in clinical practice. Further studies are required to validate these observations.

**Clinical trial number:**

Not applicable.

**Supplementary Information:**

The online version contains supplementary material available at 10.1186/s40360-026-01105-3.

## Highlights

Sex differences: Males more inclined to gastrointestinal disorders.

Indication differences: Diabetic patients more inclined to gastrointestinal disorders (vs. weight control).

**Supplementary Information:**

The online version contains supplementary material available at 10.1186/s40360-026-01105-3.

## Introduction

Type 2 diabetes (T2D) is a chronic condition marked by the progressive disruption of hyperglycemia and insulin glucose feedback mechanisms [1]. Data from the International Diabetes Federation Diabetes Atlas reveals a global uptrend in the prevalence of T2D, projected to reach 783 million among individuals aged 20 to 79 by 2045 [2]. Metabolic irregularities in various organ systems contribute to T2D development, including compromised insulin signaling in skeletal muscle and adipose tissue, changes in glucose, lipid, and amino acid utilization by the liver, and dysregulation of insulin and glucagon secretion by pancreatic islet cells, particularly β cells and α cells [3].

Traditionally, two types of therapeutic agents have been used to treat T2D: insulin or its analogues, such as insulin lispro and insulin aspart, as well as oral medications such as glipizide, glimepiride, metformin, acarbose, pioglitazone, and saxagliptin [3]. A reduced pro-insulin effect of glucagon-like peptide-1 (GLP-1) was observed in T2D patients, prompting the development of GLP-1-based T2D treatments that restore intestinal pro-insulin effects with hyperphysiological doses of GLP-1 receptor agonists [4]. In recent years, novel T2D therapies targeting GLP-1 and glucose-dependent insulin-promoting peptide (GIP) receptors have emerged rapidly [5–7], offering new method for managing blood glucose, body weight, and lipid metabolism [8].

Tirzepatide, a first-class and the only receptor agonist of double GLP-1 and GIP, is formulated as a synthetic linear peptide containing 39 amino acids based on the natural GIP sequence. It boasts GIP receptor binding affinity akin to natural GIP, with GLP-1 receptor affinity five times lower than natural GLP-1. Tirzepatide significantly lowers blood sugar levels, enhances insulin sensitivity, and promotes over 20% body weight reduction, thereby improving lipid metabolism [9]. In May 2022, the FDA approved tirzepatide (MOUNJARO) as an anti-hyperglycemic drug for enhancing blood sugar control in adult diabetes patients [10]. Subsequently, tirzepatide demonstrated substantial and lasting weight reductions in obese individuals over a 72-week span, leading to the approval of another product (ZEPBOUND), specifically for weight management in November 2023 [11, 12]. Given its promising experimental outcomes, tirzepatide is anticipated to play a pivotal role in managing overweight/obesity and/or T2D.

The unique dual GLP-1 and GIP receptor agonism of tirzepatide may lead to a safety spectrum differing from single receptor agonism. A focused analysis of its adverse event patterns is necessary to complement trial data. Prior studies have employed spontaneous reporting databases for large-scale safety monitoring of tirzepatide [13–16], identifying novel adverse events. However, there was still critical knowledge gap in adverse events of tirzepatide across diverse subpopulations. For instance, gastrointestinal disorders are frequently observed adverse events associated with tirzepatide [17] but may be vary in significance. Identifying which patient subgroups are more susceptible to the specific adverse events may be help in inform risk mitigation strategies and patient counseling. Moreover, as one of the first agents simultaneously approved for type 2 diabetes and weight management, the patient population of tirzepatide is inherently heterogeneous. Understanding how adverse events manifest across these distinct indications is crucial for tailored clinical management. To our best knowledge, only a handful of studies have revealed the sex-specific differences [18, 19], yet the potential disparities among other subgroups remain largely unexplored. The FDA Adverse Event Reporting System (FAERS), a publicly accessible spontaneous reporting database, stands as the largest and most comprehensive post-marketing safety surveillance database globally [20]. The Japanese Adverse Drug Event Report (JADER) database serves as a comprehensive repository for documenting and managing drug-related adverse events reported within Japan [21]. This study focused on discerning the differences in tirzepatide adverse events across specific subgroups through the FAERS and JADER databases. The findings of this study help advance the understanding beyond simply confirming which adverse events are associated with tirzepatide, by clarifying for which patients and under what circumstances certain adverse events are more frequently reported. This study provides contribution for identification potential difference across subgroups, which can guide future research and more targeted monitoring strategies for tirzepatide in clinical practice.

## Materials and methods

### Data source and collection

Data for this study were downloaded from the FAERS and JADER databases. The data in the FAERS database covered from the second quarter of 2022 (the quarter in which tirzepatide was approved by the FDA) to the third quarter of 2024 (the most recent quarter available at the start of this study, posted on 30-Oct-2024). The duplicate reports were identified and removed following FDA’s recommendations, retaining only the most recent version of each case: when the “CASEID” is the same, the report with the latest “FDA_DT” is selected; when both the “CASEID” and “FDA_DT” are the same, the report with the largest “PRIMARYID” is chosen. The adverse event reports of tirzepatide were identified by searching for the generic name “TIRZEPATIDE” in the “prod_ai” column. Then, the reports with tirzepatide as the primary suspected (PS) drug were extracted by searching for the “PS” in the “role_cod” column. The clinical characteristics of tirzepatide-associated adverse event reports, which were selected based on the availability and quality of relevant data fields, were investigated based on the FAERS database. To reduce the deviations in different periods, the data in the JADER database spanning the same period with those in the FAERS database were extracted. The reports of tirzepatide were identified using generic name “チルゼパチド” in the “医薬品(一般名)” column and “被疑薬” in the “医薬品の関与” column. The additional details regarding data source and collection can be found in previously published articles [21–26].

### Statistical analysis

The intensity of tirzepatide-associated signals from the FAERS and JADER databases was investigated at the system organ class (SOC) and preferred term (PT) levels [27, 28], respectively. Two Frequentist methods including the reporting odds ratio (ROR) [29, 30] and the proportional reporting ratio (PRR) [31, 32], along with two Bayesian methods, including the Bayesian confidence propagation neural network (BCPNN) [31–33] and the multi-item gamma Poisson shrinker (MGPS) [31–33], were employed. The fourfold table, equations and criteria of the four disproportionality algorithms for tirzepatide signal detection are shown in Supplementary Tables S1-S2.

Based on the results of clinical characteristics and signal detection from the FAERS database, the differences in adverse events across specific subgroups were investigated using the ROR algorithm and Chi-Square Test/Fisher’s exact test. The fourfold table, criteria of ROR and Chi-Square Test/Fisher’s exact for difference detection of tirzepatide signals are shown in Supplementary Tables S3-S4.

To further verify the differences, subgroup-based stratified analyses and sensitivity analyses from the FAERS database were conducted using four disproportionality algorithms. Reports with missing or invalid clinical characteristic fields were excluded from the respective clinical characteristics, subgroup analyses, stratified analyses, and sensitivity analyses but were retained in the overall signal detection. In signal detection, subgroup analyses, stratified analyses, and sensitivity analyses, the signals of “product issues”, “injury, poisoning and procedural complications”, “social circumstances”, and consistent with indications, which might cause by the product issues, inappropriate medication, disease progression, ineffective treatment, or other factors, were excluded. All data processing and statistical analyses were performed using Python 3 programming language in Jupyter Notebook version 6.4.12. The additional details regarding statistical analysis can be found in previously published articles [22–26].

## Results

### Signal detection and clinical characteristic

**Fig. 1:**
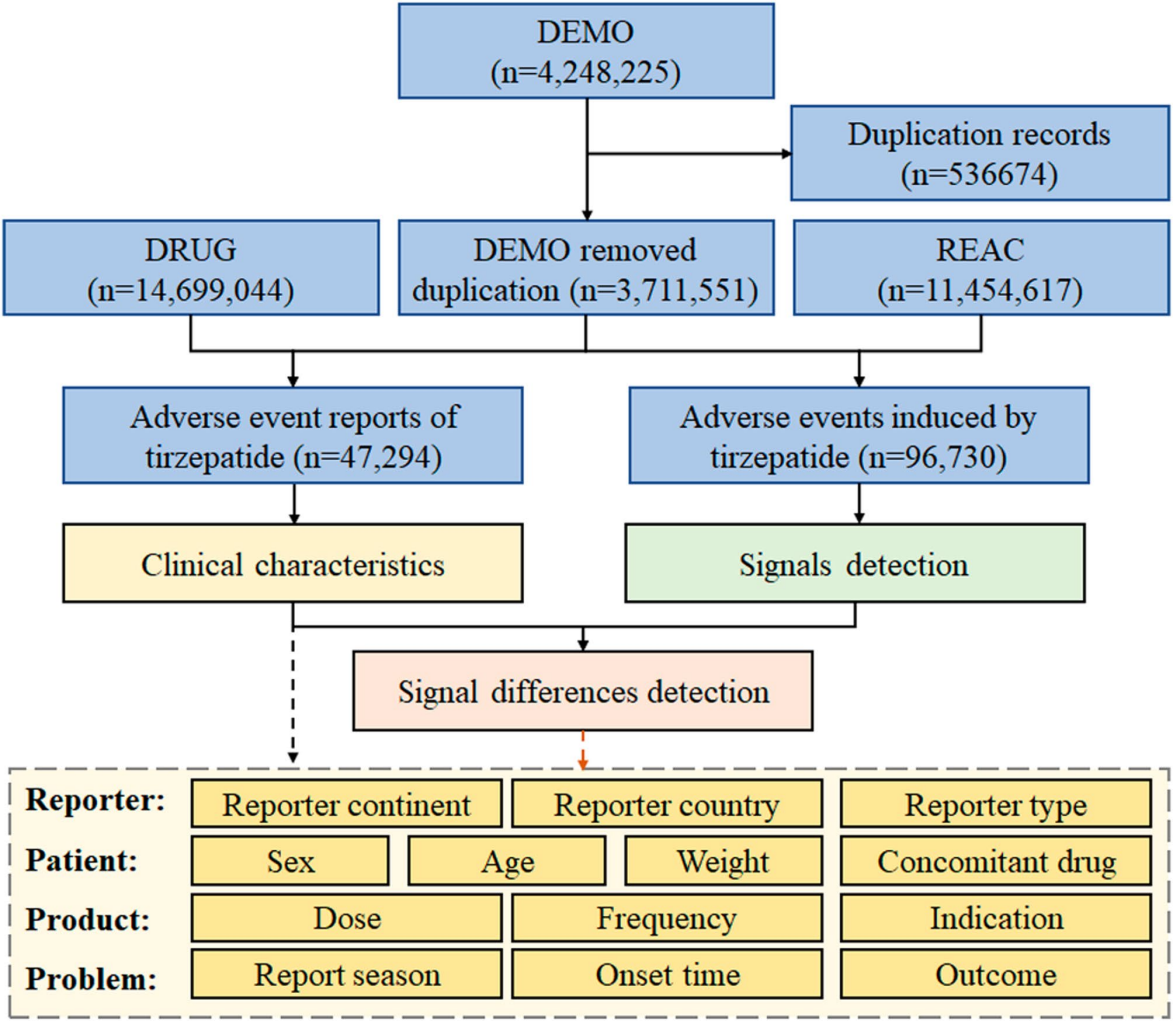
Flow diagram of data collection and analysis of tirzepatide-associated adverse events from the FAERS database. Abbreviations: DEMO, patient demographic and administrative information; DRUG, drug information; REAC, coded for the adverse events

A total of 47,294 reports and 96,730 adverse events associated with tirzepatide were identified from the FAERS database. A flow diagram of data collection and analysis of tirzepatide-associated adverse events from the FAERS database is shown in Fig. 1. The number and signal strength of tirzepatide at the SOC level from the FAERS database are described in Supplementary Table S5. Statistically, it was found that tirzepatide-associated adverse events involved 27 SOCs. The significant SOCs that met four criteria were “injury, poisoning and procedural complications” and “gastrointestinal disorders”. A total of 118 signals at the PT level were detected after conforming to the four algorithms simultaneously. There were 34 tirzepatide-unrelated signals, which might cause by the product issues, inappropriate medication, disease progression or ineffective treatment. The number and signal strength of 34 tirzepatide-unrelated signals at the PT level from the FAERS database are listed in Supplementary Table S6. After excluding the 34 tirzepatide-unrelated signals, 84 significant disproportionality PTs from the FAERS database are shown in Supplementary Table S7.

From the JADER database, a total of 250 reports and 388 adverse events associated with tirzepatide were identified. The number and signal strength of tirzepatide at the SOC level from the JADER database are described in Supplementary Table S8. The significant SOCs that met four criteria were “gastrointestinal disorders”, “metabolism and nutrition disorders” and “hepatobiliary disorders”. A total of 22 signals at the PT level were detected after conforming to the four algorithms simultaneously. No tirzepatide-unrelated signals were detected at the PT level from the JADER database. Number and signal strength of tirzepatide-related signals at the PT level from the JADER database are described in Supplementary Table S9.

The “gastrointestinal disorders” was the only significant SOC that met the criteria of all four disproportionality algorithms in both the JADER and FAERS databases. “Metabolism and nutrition disorders” and “hepatobiliary disorders” were SOCs satisfied the criteria of all four algorithms in the JADER database but not significant in the FAERS database.

**Fig. 2:**
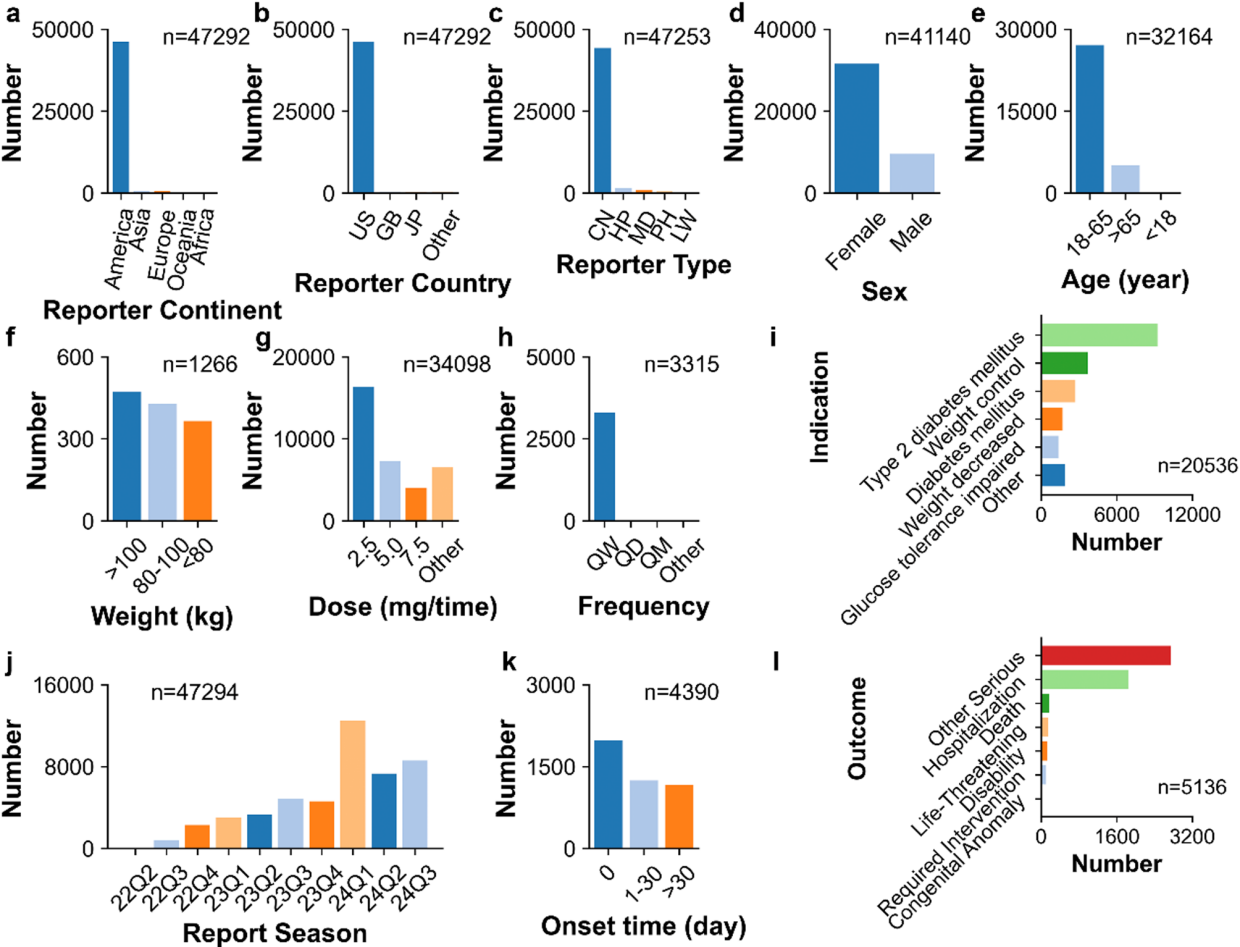
Clinical characteristics of tirzepatide-associated adverse event reports from the FAERS database. (**a**) Reporter continent. (**b**) Reporter country. (**c**) Reporter type. (**d**) Sex. (**e**) Age. (**f**) Weight. (**g**) Dose. (**h**) Frequency. (**i**) Indication. (**j**) Report season. (**k**) Onset time. (**l**) Outcome. Abbreviations: US, United States; GB, United Kingdom; JP, Japan; CN, consumer; HP, Health-professional; MD, Physician; PH, pharmacist; LW, lawyer; QW, quaque week; QD, quaque day; QM, quaque month

**Fig. 3:**
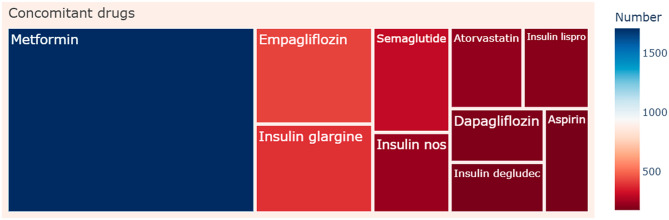
Rectangular tree plot of top 10 ranked concomitant drugs of tirzepatide-associated adverse event reports from the FAERS database

The clinical characteristics of 47,294 tirzepatide-associated adverse event reports from the FAERS database are illustrated in Figs. 2 and 3. Concerning the distribution of adverse event reports by reporter country from the FAERS database, the United States accounted for 98% of all reports, positioning America (*n* = 46,162) as the primary source. As FAERS is overwhelmingly United States-dominated, differences across continent subgroups within FAERS may introduce systematic reporting bias, as acknowledged but not fully addressed. Weight data were available for only 1,266 patients. The highest proportion of patients belonged to the weight category of over 100 kg (*n* = 473), making up 37% of the total cases. A weekly administration frequency (QW) was the most common dosing frequency (*n* = 3,292, 99%). There were insufficient data on different continent, weight and different dosing frequency. Thus, the differences across continent, weight, and dosing frequency subgroups require further study. The differences in adverse events across reporter type, sex, age, dose, indication, report year, onset time, and outcome subgroups were specifically investigated.

### Differences across reporter type subgroups

**Fig. 4:**
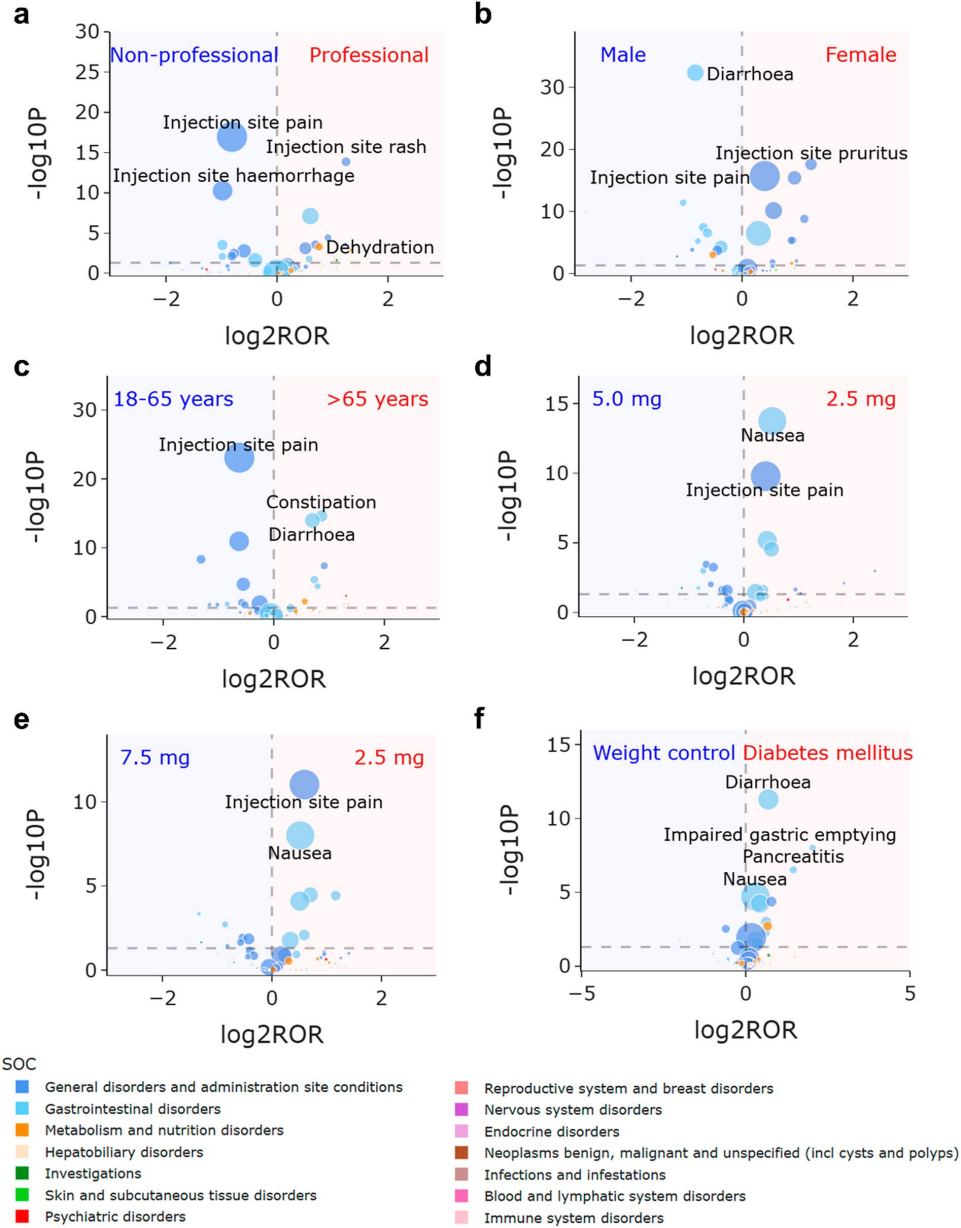
Volcano plots for difference detection of tirzepatide signals regarding reporter and patient from the FAERS database. (**a**) Difference between professional and non-professional reporters. (**b**) Difference between female and male patients. (**c**) Difference between patients with age > 65 years and 18–65 years. (**d**) Difference between patients taking signal dose 2.5 mg and 5.0 mg. (**e**) Difference between patients taking signal dose 2.5 mg and 7.5 mg. (**f**) Difference between patients with indication of diabetes mellitus and weight control. The colors of each point represent different SOCs. The sizes of each point represent the number of reports of each PT induced by tirzepatide. In these plots, 84 tirzepatide-associated signals are shown

Excluding reports from unknown reporters, consumers (*n* = 44,275) reported majority of adverse event reports at 94% from the FAERS database. Figure 4a depicts volcano plot highlighting the variations in tirzepatide-related signals across reporter type subgroup. Both professional (including physicians, health-professionals, pharmacists, and other health-professionals) and non-professional (including consumers and lawyers) reporters contributed to reports of “general disorders and administration site conditions,” with nuances in focus. Professional reporters were notably more inclined to report “injection site rash”, while non-professionals were significantly more frequently to report “injection site pain” (Professional vs. Non-professional: “injection site rash”, ROR 2.369, 95% CI 1.889–2.972, *P* < 0.001; “injection site pain”, ROR 0.570, 95% CI 0.500-0.649, *P* < 0.001). Moreover, “dehydration” was predominantly reported by professionals (Professional vs. Non-professional: “dehydration”, ROR 1.690, 95% CI 1.252–2.281, P 0.001).

Number and signal strength of tirzepatide-related signals at the PT level from the FAERS database stratified by professional and non-professional reporter are show in Supplementary Tables S10-S11. The reporter type-based stratified analyses analogously revealed strong signals of “injection site rash” and “dehydration” by professional reporter, while notably weaker signal of these adverse events by non-professional reporter [Professional vs. Non-professional: “injection site rash”, 29.85 (24.11–36.96) vs. 15.72 (14.31–17.27); “dehydration”, 5.30 (3.99–7.05) vs. 2.59 (2.35–2.86)]. Given that consumers account for 94% proportion in all the reports, these results highlight the urgent necessity of strengthening educational initiatives aimed at consumers and their families regarding specific injection site events and dehydration.

### Differences across sex subgroups

Regarding patients, females (*n* = 31,548) represented 77% of the cases, outnumbering males (*n* = 9,592) at 23%. This was consistent with findings from large-scale epidemiological studies demonstrating a higher prevalence of obesity among adult females compared to males [34, 35]. Figure 4b depicts volcano plot highlighting the variations in tirzepatide-related signals across sex subgroups. Females were notably more frequently to report “general disorders and administration site conditions” like “injection site pruritus”, while males were significantly more inclined to report “gastrointestinal disorders” such as “diarrhoea” (Female vs. Male: “injection site pruritus”, ROR 2.367, 95% CI 1.939–2.889, *P* < 0.001; “diarrhoea”, ROR 0.558, 95% CI 0.506–0.614, *P* < 0.001).

Number and signal strength of tirzepatide-related signals at the PT level from the FAERS database stratified by males and females are show in Supplementary Tables S12-S13. The sex-based stratified analyses revealed a stronger signal of “diarrhoea” for males [Male vs. female: “diarrhoea”, 3.34 (3.08–3.61) vs. not significant]. Furthermore, the signal strength of “injection site pruritus” was similar in the male and female stratification [Male vs. female: “injection site pruritus”, 12.53 (10.32–15.22) vs. 12.45 (11.61–13.36)], suggesting that the sex differences in this administration site event are not unique to tirzepatide but may also exist in other drugs.

### Differences across age subgroups

Majority of patients fell into the age group of 18 to 65 years (*n* = 27,088, 84%), followed by over 65 years (*n* = 5,059, 16%). Figure 4c depicts volcano plot highlighting the variations in tirzepatide-related signals across age subgroups. Patients over 65 years of age were notably more frequently to report “gastrointestinal disorders” like “constipation”, while those aged 18–65 years were significantly more frequently to report “general disorders and administration site conditions,” such as “injection site pain” (> 65 years vs. 18–65 years: “constipation”, ROR 1.818, 95% CI 1.565–2.112, *P* < 0.001; “injection site pain”, ROR 0.649, 95% CI 0.597–0.707, *P* < 0.001).

Number and signal strength of tirzepatide-related signals at the PT level from the FAERS database stratified by patients with 18 to 65 years and over 65 years are show in Supplementary Tables S14-S15. The age-based stratified analyses revealed similar trend but with small differences [> 65 years vs. 18–65 years: “constipation”, 4.08 (3.58–4.64) vs. 3.98 (3.67–4.31); “injection site pain”, 16.54 (15.24–17.96) vs. 20.63 (19.95–21.33)], suggesting that the age differences in these events may also exist in other drugs. The differences in tirzepatide-related signals among minors are subject to further investigation due to a lack of data on patients under 18 years old.

### Differences across dose subgroups

The recommended doses of tirzepatide are 2.5 mg, 5.0 mg, 7.5 mg, 10.0 mg, 12.5 mg, or 15.0 mg administered via subcutaneous injection weekly. Excluding cases with missing or incomparable dose unit data (“dose_unit” column as MG/M**2 or IU), effective dose data were available for 34,098 cases. Majority of doses were 2.5 mg (*n* = 16,354, 48%), 5.0 mg (*n* = 7,264, 21%), and 7.5 mg (*n* = 3,994, 12%). Figure 4d and e depict volcano plots highlighting the variations in tirzepatide-related signals across dose subgroups (2.5 mg, 5.0 mg and 7.5 mg, the three most common doses). Common adverse events related to tirzepatide, such as “nausea” and “injection site pain”, were more frequently reported at the 2.5 mg dose (2.5 mg vs. 5.0 mg: “nausea”, ROR 1.435, 95% CI 1.308–1.574, *P* < 0.001; 2.5 mg vs. 5.0 mg: “injection site pain”, ROR 1.319, 95% CI 1.212–1.437, *P* < 0.001; 2.5 mg vs. 7.5 mg: “injection site pain”, ROR 1.509, 95% CI 1.340–1.700, *P* < 0.001; 2.5 mg vs. 7.5 mg: “nausea”, ROR 1.432, 95% CI 1.266–1.620, *P* < 0.001).

Number and signal strength of tirzepatide-related signals at the PT level from the FAERS database based on reports taking 2.5 mg, 5.0 mg and 7.5 mg doses are presented in Supplementary Tables S16-S18. The dose-based sensitivity analyses revealed stronger signals of “nausea” and “injection site pain” at 2.5 mg dose when compared with 5.0 mg and 7.5 mg doses [2.5 mg vs. 5.0 mg vs. 7.5 mg: “nausea”, 5.58 (5.34–5.83) vs. 3.85 (3.54–4.18) vs. 3.85 (3.43–4.32); “injection site pain”, 15.26 (14.64–15.92) vs. 11.23 (10.42–12.11) vs. 9.75 (8.72–10.91)]. As a starting dose of 2.5 mg was the recommended, the results indicate that these common adverse events may be self-limiting and tend to improve as tirzepatide is continuously used.

### Differences across indication subgroups

**Fig. 5:**
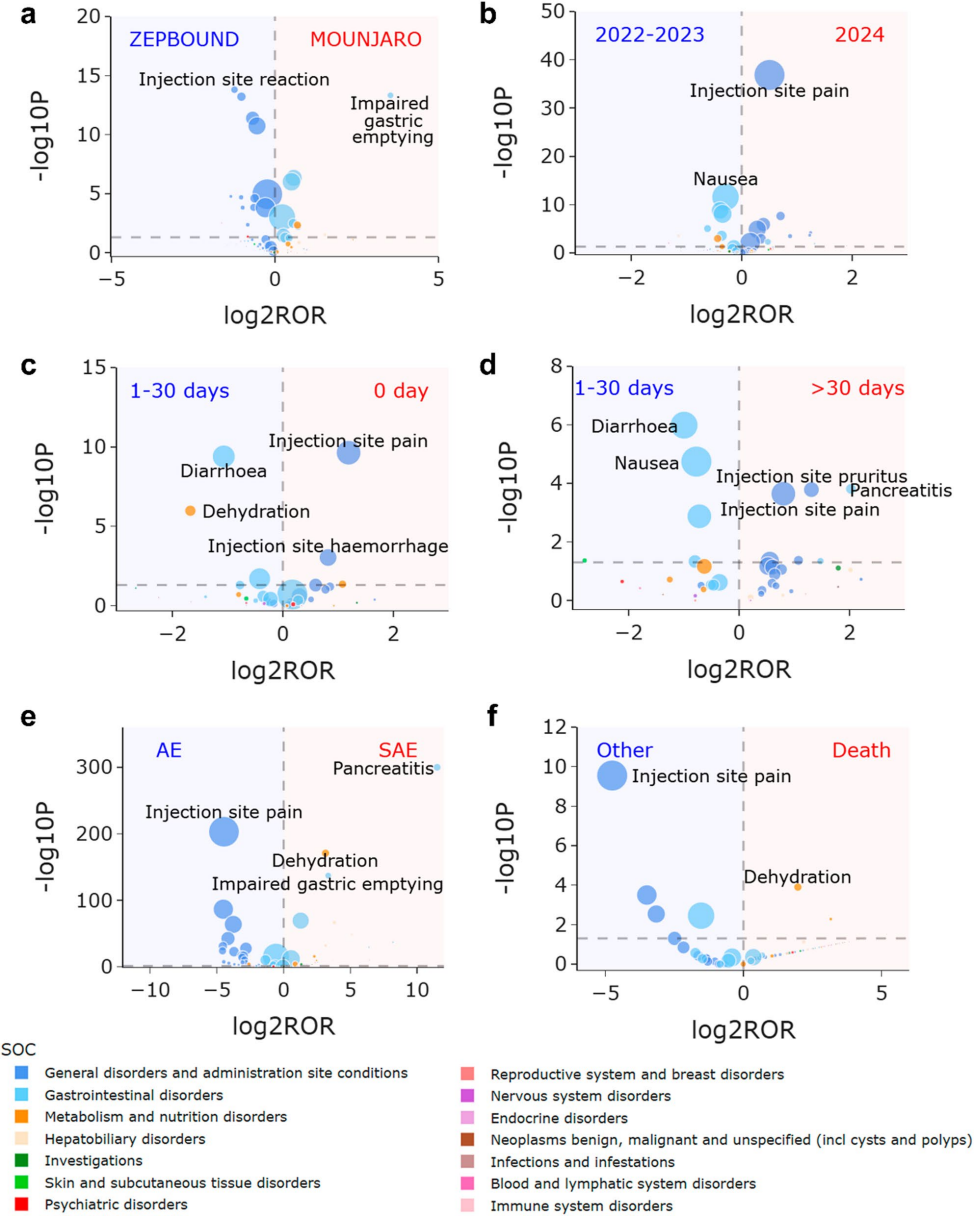
Volcano plots for difference detection of tirzepatide signals regarding problem and product from the FAERS database. (**a**) Difference between Mounjaro and Zepbound. (**b**) Differences between year 2024 and 2022–2023. (**c**) Difference between onset time 0 day and 1–30 days. (**d**) Difference between onset time > 30 days and 1–30 days. (**e**) Difference between SAE and AE. (**f**) Difference between patients with death outcome and others. The colors of each point represent different SOCs. The sizes of each point represent the number of reports of each PT induced by tirzepatide. In these plots, 84 tirzepatide-associated signals are shown. Abbreviations: AE, adverse event; SAE, serious adverse event

Out of 20,536 reported indications, “type 2 diabetes mellitus” (*n* = 9,218) accounted for the majority at 45%, followed by “weight control” (*n* = 3,664) at 18%, “diabetes mellitus” (*n* = 2,701) at 13%, and “weight decreased” (*n* = 1,685) at 8%. Figures 4f and 5a depict volcano plots highlighting the variations in tirzepatide-related signals across indication subgroups. Compared with patients in weight control purpose (including reports with indications as “weight control” and “weight decreased”), tirzepatide used in patients with diabetes mellitus (including reports with indications as “type 2 diabetes mellitus” and “diabetes mellitus”) was significantly more frequently to report “gastrointestinal disorders”, such as “diarrhoea”, “impaired gastric emptying” and “pancreatitis” (Diabetes mellitus vs. Weight control: “diarrhoea”, ROR 1.612, 95% CI 1.406–1.848, *P* < 0.001; “impaired gastric emptying”, ROR 4.103, 95% CI 2.432–6.921, *P* < 0.001; “pancreatitis”, ROR 2.728, 95% CI 1.828–4.070, *P* < 0.001). Additionally, MOUNJARO, primarily used for diabetes control, was also significantly more frequently to report “gastrointestinal disorders” like “impaired gastric emptying”, whereas ZEPBOUND, specifically used for weight management, was significantly more frequently to report “general disorders and administration site conditions,” such as “injection site reaction” (MOUNJARO vs. ZEPBOUND: “impaired gastric emptying”, ROR 11.563, 95% CI 4.312–31.004, *P* < 0.001; “injection site reaction”, ROR 0.424, 95% CI 0.338–0.531, *P* < 0.001).

Number and signal strength of tirzepatide-related signals at the PT level from the FAERS database based on reports with diabetes mellitus and weight control indication are presented in Supplementary Tables S19-S20. The indication-based sensitivity analyses revealed stronger signals of “diarrhoea”, “impaired gastric emptying” and “pancreatitis” for patients with diabetes mellitus when compared with patients for weight control purpose [Diabetes mellitus vs. Weight control: “diarrhoea”, 2.97 (2.77–3.19) vs. not significant; “impaired gastric emptying”, 23.90 (19.83–28.81) vs. 5.57 (3.40–9.11); “pancreatitis”, 9.27 (7.84–10.96) vs. 3.35 (2.32–4.83)].

### Differences across report year subgroups

The number of tirzepatide-associated reports increased from the second quarter of 2022 to the third quarter of 2024, significantly spiking in the first quarter of 2024. Figure 5b depicts volcano plot highlighting the variations in tirzepatide-related signals across report time subgroups. In the year 2024, there was a significantly higher frequency of reporting “general disorders and administration site conditions,” such as “injection site pain” (2024 vs. 2022–2023: “injection site pain”, ROR 1.417, 95% CI 1.343–1.495, *P* < 0.001).

Number and signal strength of tirzepatide-related signals at the PT level from the FAERS database based on reports in years 2022–2023 and 2024 are presented in Supplementary Tables S21-S22. The report year-based sensitivity analyses revealed stronger signals of “injection site pain” in 2024 [2024 vs. 2022–2023: “injection site pain”, 17.70 (17.14–18.28) vs. 12.02 (11.50-12.56)]. Since the ZEPBOUND may be more frequently to induce “general disorders and administration site conditions,” these differences among report year may be somewhat related to the approval and market launch of the product in November 2023.

### Differences across onset time subgroups

Excluding false and missing data, 4,390 reports detailed the effective onset time of tirzepatide-associated adverse events. Among these, 45% of adverse events occurred on the first day of administration (*n* = 1,980). Figure 5c and d depict volcano plots highlighting the variations in tirzepatide-related signals across onset time subgroups. Compared with 1–30 days of medication, injection site events were more frequently to occur on the day and 30 days after, but the focus areas differed. On the day of medication there was a significantly higher frequency of reporting “injection site haemorrhage” (0 day vs. 1–30 days: “injection site haemorrhage”, ROR 1.763, 95% CI 1.255–2.477, *P* < 0.001), while after 30 days there was a significantly higher frequency of reporting “injection site pruritus” (> 30 days vs. 1–30 days: “injection site pruritus”, ROR 2.478, 95% CI 1.521–4.034, *P* < 0.001). Moreover, “pancreatitis” had a significantly higher frequency of reporting after 30 days (> 30 days vs. 1–30 days: “pancreatitis”, ROR 4.068, 95% CI 1.851–8.939, *P* < 0.001).

Number and signal strength of tirzepatide-related signals at the PT level from the FAERS database based on reports occur on the day, 1–30 days and over 30 days of medication are presented in Supplementary Tables S23-S25. The onset time-based sensitivity analyses revealed a stronger signal of “injection site haemorrhage” on the day, while stronger signals of “injection site pruritus” and “pancreatitis” over 30 days [0 day vs. 1–30 days vs. over 30 days: “injection site haemorrhage”, 17.35 (14.54–20.70) vs. 9.79 (7.32–13.10) vs. 14.09 (10.84–18.32); “injection site pruritus”, 8.58 (6.33–11.63) vs. 7.73 (5.17–11.56) vs. 19.21 (14.55–25.35); “pancreatitis”, not significant vs. not significant vs. 15.46 (10.64–22.44)]. This result aids in understanding the onset time pattern of tirzepatide.

### Differences across outcome subgroups

Out of 47,294 reports, 4,652 reports led to 5,136 serious outcomes, classified into categories including “death,” “life-threatening,” “hospitalization-initial or prolonged,” “disability,” “congenital anomaly,” “required intervention to prevent permanent impairment/damage,” and “other serious.” “Other serious” was the most common outcome, accounting for 53% of the serious cases. Figure 5e and f depict volcano plots highlighting the variations in tirzepatide-related signals across outcome subgroups. Injection site events predominantly resulted in non-severe outcomes. Notably, “pancreatitis” and “dehydration” more frequently led to severe outcomes (SAE vs. AE: “pancreatitis”, ROR 2886.631, 95% CI 405.631-20542.399, *P* < 0.001; “dehydration”, ROR 8.787, 95% CI 7.313–10.558, *P* < 0.001), with “dehydration” being more frequently reported in cases resulting in death (Death vs. Other: “dehydration”, ROR 3.916, 95% CI 1.844–8.318, *P* < 0.001).

To further investigate the serious adverse events, this study included only reports that led to severe outcomes from the FAERS database for sensitivity analysis. Number and signal strength of tirzepatide-related signals at the PT level from the FAERS database based on reports with serious outcomes are show in Supplementary Table S26. Compared with the results involving all adverse events in Supplementary Table S7, the outcome-based sensitivity analysis revealed strong signals for “pancreatitis” and “dehydration” in serious adverse event reports [All adverse events vs. Serious adverse events: “pancreatitis”, 7.84 (7.10–8.65) vs. 61.51 (55.66–67.97); “dehydration”, 2.92 (2.67–3.20) vs. 12.73 (11.27–14.39)]. These findings suggest that particular attention should be paid to pancreatitis and dehydration to prevent serious outcomes.

## Discussion

The use of spontaneous reporting system databases is valuable for signal detection. It has also been well-established in the pharmacovigilance literature for generating and exploring initial hypotheses regarding subgroup differences (e.g., sex-based subgroup differences [18, 36–40]). This study provides contribution for identification potential difference across subgroups. However, it is important to note that analyzing clinical differences using spontaneous reporting system databases presents inherent challenges due to confounding factors. In this study, several methodological strategies were implemented to enhance the robustness of subgroup analyses within these acknowledged constraints.

Firstly, the ROR algorithm and Chi-Square Test/Fisher’s exact test were employed to analyze differences in adverse events across subgroups. In this study, several subgroup comparisons were based on very small cell counts (*N* ≤ 40 or any of a, b, c, d < 5). Fisher’s exact test is particularly suitable for such low-count scenarios, as it calculates the exact p-value using the hypergeometric distribution, thereby ensuring statistical robustness and reliability. For subgroups with *N* ≥ 40 and all of a, b, c, d ≥ 5, the Chi-Square Test was deemed more appropriate. Currently, the Chi-Square Test/Fisher’s exact test is widely accepted in the literature for comparing two groups in disproportionality analyses [41–46]. Based on these considerations, this study adopted the integrated use of ROR and Chi-Square Test/Fisher’s exact test, which enables quantification of the magnitude of signal differences between groups (via ROR) and assessment of statistical significance (via Chi-Square Test/Fisher’s exact test).

Secondly, stratified analysis and sensitivity analysis were conducted to validate the results of the ROR and Chi-Square Test/Fisher’s exact test. Specifically, only reports with relevant subgroup information were included, thereby reducing the impact of reports with unreported information. Additionally, key potential confounding factors were controlled, which facilitates more accurate exploration of the differences in adverse events within specific subgroups.

Thirdly, it is important to emphasize that the study results merely reflect associations observed in the data. The observed differences may arise from biological/clinical heterogeneity in drug risks or confounding factors, such as differential reporting behaviors (e.g., underreporting in certain populations). It is important to avoid overly causal interpretation of the results within the spontaneous reporting system database alone. Therefore, the results of this study still need to be validated through extensive literature reviews and clinical expertise. Subsequently, this study discussed the main adverse events with identified subgroup differences by analyzing relevant literature. Discrepancies that could not be reasonably explained were excluded from the final study conclusion.

### General disorders and administration site conditions

The SOC of “general disorders and administration site conditions” encompassed 31 PTs from the FAERS database, predominantly related to administration site events. Clinical trial data indicate that 1.9-8.0% of tirzepatide-treated patients experienced injection site reactions [47, 48]. The findings regarding outcome subgroups corroborate earlier studies that these events predominantly resulted in non-severe outcomes [49, 50]. No relevant signals were detected in the JADER database, which may be attributed to the fact that JADER primarily collects reports of serious adverse events. The consistency of these findings with existing literatures underscores the reliability of this study.

The subgroup analysis confirmed prior findings of a higher frequency of administration site events in female patients [18, 19]. Further sex-based stratified analyses revealed similar signal strength of administration site events in the male and female stratification, suggesting that the sex differences in this administration site event are not unique to tirzepatide but may also exist in other drugs. Kim et al. analyzed sex differences in adverse event reporting rates following COVID-19 vaccination, with injection site pruritus showing the largest difference (9.00 [5.34–15.17]) [51]. Females may experience greater distress from externally visible adverse events, which could be explained by heightened societal expectations and internalized pressures concerning appearance [52]. This may lead to a reporting bias in which cosmetically salient adverse events are disproportionately reported by females [52]. Consequently, the difference observed in administration site events may reflect differences in individual reporting tendencies. Based solely on these data, it is not feasible to conclude that there are sex-based differences in the occurrence of administration site events. Future research should apply the Gender Hypothesis more rigorously to additional datasets, which may offer further insight into the underlying causes of sex disparities in adverse events [52].

Additionally, this study provides novel insights into the onset time pattern of administration site events. Both on the day of medication and after 30 days, there was a significantly higher frequency of reporting administration site events compared with 1–30 days, though the focus areas differed. On the day of medication, attention should be directed towards “injection site hemorrhage”, while after more than 30 days, emphasis should shift to “injection site pruritus”. Injection site hemorrhage is primarily an immediate, mechanical complication resulting from the needle puncture itself, such as direct capillary injury or inadequate local hemostasis. Consequently, it manifests almost instantaneously or within a very short post-injection window. In contrast, injection site pruritus represents a local sensory and frequently immune-mediated response. It may be attributed to several delayed factors, such as injection site fibrosis, sensitization of local nerve endings, stimulation from drug accumulation or metabolites after long-term use, or the H1R/TRPV1 signaling pathway [53], and therefore usually occurs during the later stages of medication. These finding underscores the importance of time-dependent monitoring strategies to accurately identify and manage different injection site adverse events.

### Gastrointestinal disorders

The “gastrointestinal disorders” was the only significant SOC in both the JADER and FAERS databases. “Nausea”, “diarrhoea”, and “vomiting” emerged as the most common adverse events. Throughout the clinical trials, nausea (12%-24%), diarrhoea (12%-22%), and vomiting (2%-13%) were the most commonly reported gastrointestinal events associated with tirzepatide [54–56], consistent with the results of this study. While gastrointestinal events were transient and of mild-to-moderate severity [56], “pancreatitis” was significantly more frequent leading to severe outcomes and occurred after 30 days of medication. Although less than 5% of pancreatitis cases are drug-induced and tirzepatide appears to exhibit safety regarding pancreatitis risk [57], there is emerging evidence of a substantial risk associated with GLP-1 receptor agonists [58]. The finding suggests that long-term monitoring of pancreatitis and its related symptoms may be crucial to prevent serious consequences. Notably, the finding should be interpreted with caution due to limited number of reports.

Subgroup analysis results from this study revealed that gastrointestinal disorders were more frequently detected in male patients, aligning with previous studies [18, 19]. This observed sex disparity may be attributable to a combination of behavioral and biological factors. From a behavioral perspective, males are often linked with unhealthy lifestyles, such as poor diet, stress, physical inactivity, smoking, and excessive alcohol consumption, leading to a higher prevalence of non-malignant upper gastrointestinal diseases [59]. Biologically, estrogen’s potential protective role in mucosal defense and gastrointestinal epithelia protection might contribute to varying susceptibilities [60–62]. Gender distribution analysis has indicated that males dominate among adult inflammatory bowel disease patients [63]. A meta-analysis of epidemiological studies revealed a higher incidence of inflammatory bowel disease in males, with a male-to-female ratio of 1.64:1 for Crohn’s disease and 1.29:1 for ulcerative colitis [64]. Furthermore, male patients are more likely to have severe Crohn’s disease and experience penetrating or structural complications of the disease [65–67]. It is important to note that sex-based difference observed in gastrointestinal disorders requires cautious interpretation.

This study further revealed that individuals with diabetes mellitus were more susceptible to gastrointestinal disorders. This may be attributed to the increased sensitivity of the digestive system and the use of concomitant drugs in patients with diabetes mellitus. As a systemic disease, diabetes mellitus induces vascular endothelial damage, promotes the development of chronic inflammation, and causes organic and functional lesions in multiple systems and organs [68]. Gastrointestinal disorders are common complications of diabetes mellitus, including gastroparesis, nonalcoholic fatty liver disease, gastroesophageal reflux disease, and chronic diarrhea [68, 69]. Chen et al. conducted a Mendelian randomization study to investigate the associations between type 2 diabetes and gastrointestinal diseases. The results demonstrated that genetic liability to type 2 diabetes was significantly associated with an elevated risk of 12 gastrointestinal conditions, including gastroesophageal reflux disease, gastric ulcer, acute gastritis, chronic gastritis, irritable bowel syndrome, diverticular disease, acute pancreatitis, cholelithiasis, cholelithiasis with cholecystitis, nonalcoholic fatty liver disease, liver cirrhosis, and ulcerative colitis [70]. Moreover, the observed difference may be also affected by the use of concomitant drugs in the diabetic patient population. Metformin, the most prescribed medication for type 2 diabetes mellitus, is well established to cause a high incidence of gastrointestinal adverse events [71]. Gastrointestinal adverse events induced by metformin are associated with dose escalation, immediate-release formulations, alterations in gut microbiota, epigenetic susceptibility, inhibition of organic cation transporters, as well as interactions with serotonin, histamine, and enterohepatic circulation [72]. Given metformin’s well-established gastrointestinal events, its co-administration with tirzepatide is a highly plausible alternative explanation for the stronger gastrointestinal disorders in diabetic patients. For patients experiencing gastrointestinal events during concurrent metformin and tirzepatide therapy, management strategies include appropriate titration of immediate-release metformin, use of extended-release metformin and gut microbiome modulators, as well as alternative pharmacological therapies when metformin cannot be tolerated at all [73]. The findings underscore the importance of physicians remaining vigilant about the development of gastrointestinal complications in patients with diabetes mellitus. Future research with larger, population-based samples and detailed stratification is needed to elucidate the precise mechanisms behind this association.

### Metabolism and nutrition disorders

“Dehydration”, a common signal within metabolism and nutrition disorders, was identified from both the FAERS and JADER databases. Given that tirzepatide is associated with gastrointestinal events such as nausea, vomiting, and diarrhea, these events can potentially lead to dehydration in severe cases. Furthermore, concomitant hypoglycemic drugs use may increase the risk of dehydration. Kido et al. call for documenting the potential drug interactions between guideline-directed medical therapy agents and tirzepatide in major drug interaction databases, particularly regarding risks of dehydration [74]. Subgroup analysis results indicate that “dehydration” was more frequently reported in cases resulting in severe outcomes, including death, underscoring the significance of addressing dehydration. Severe dehydration may be accompanied by symptoms such as acute kidney injury [75] and systemic acidosis [76]. Clinicians should adhere to standard titration protocols, closely monitor blood pressure and renal function, and exercise caution in patients with complex medication regimens, thereby maximizing the therapeutic benefits of tirzepatide while minimizing risks [76]. This study further revealed that “dehydration” was predominantly reported by professionals. This finding underscores the importance of enhancing patient education and monitoring for dehydration-related symptoms following medication administration to prevent serious outcomes. Due to the limited number of reports, this result still requires further verification.

### Hepatobiliary disorders

The results revealed particularly strong signals for “hepatobiliary disorders” such as “cholangitis acute” and “cholecystitis” in the JADER database. Although “hepatobiliary disorders” was not the significant SOC associated with tirzepatide from the FAERS database, several signals such as “cholelithiasis”, “cholecystitis”, “biliary colic”, and “cholangitis acute” within hepatobiliary disorders were also identified at the PT level. A systematic review and meta-analysis highlighted an increased risk of gallbladder or biliary diseases with the use of GLP-1 receptor agonists, including albiglutide, dulaglutide, exenatide, liraglutide, lixisenatide, and semaglutide [77]. Another systematic review and meta-analysis also emphasized the heightened risk of gallbladder or biliary diseases associated with tirzepatide, underscoring the need for vigilance in clinical practice [57]. This study highlights the necessity for enhanced monitoring of hepatobiliary disorders during tirzepatide treatment, though it was not the significant SOC associated with tirzepatide from the FAERS database.

### Strengths and limitations

This study offers a comprehensive description and characterization of the differences of tirzepatide’s adverse events across subgroups, revealing several critical safety findings. Firstly, males were significantly more inclined to report “gastrointestinal disorders”. Secondly, compared with patients in weight control purpose, tirzepatide used in patients with diabetes mellitus was significantly more frequently to report “gastrointestinal disorders”. This study provides a basis for potential targeted monitoring of tirzepatide use.

While this study benefits from large-sample real-world investigations and data mining techniques, several limitations require consideration. Firstly, the utilization of the FAERS and JADER as spontaneous reporting systems may engender incomplete data, potentially predisposing results to biases. The substantial prevalence of missing data underscores the necessity for validation through additional datasets. Both FAERS and JADER are inherently subject to reporting bias, including underreporting and stimulated reporting. Reporting behavior may be influenced by event severity, regulatory actions, and clinical awareness, potentially leading to an overrepresentation of serious adverse events and underreporting of milder events. Moreover, differences in reporting practices and regulatory requirements between FAERS and JADER may partially account for discrepancies in signal strength observed between the two databases. Secondly, the lack of total patient population data receiving tirzepatide treatment hinders the calculation of adverse event incidence rates. Thirdly, residual confounding factors confounding is a key limitation of this study. For instance, the concurrent use of hypoglycemic medications may heighten the risk of hypoglycemia. Furthermore, the therapeutic efficacy of traditional Chinese medicines in blood sugar regulation and amelioration of liver and gastrointestinal maladies is well-documented [78–81]. Additionally, the overrepresentation of females in sex-based subgroup analyses could be attributed to the prevalent use of tirzepatide for weight management, which typically garners greater demand among females than males, potentially skewing the outcomes. However, the present study inadequately addresses the impact of these confounding factors. Fourthly, the differential market availability of tirzepatide across various countries, with some regions yet to introduce the drug, may introduce additional biases into the analysis. Fifthly, while this study compared the reporting rates of tirzepatide with those of all other drugs as comparators, future investigations should meticulously select appropriate comparators for a more in-depth exploration of tirzepatide-associated adverse events. Sixthly, a causal relationship between tirzepatide and adverse events was not definitively established. The results should be interpreted as generating hypotheses for clinical difference, which require prospective clinical studies for confirmation [82, 83]. Despite these limitations, the study findings offer valuable insights for professionals to diligently monitor patients for adverse event profile of tirzepatide.

## Conclusion

This study provides contribution for identification potential difference across subgroups, which can guide future research and more targeted monitoring strategies for tirzepatide in clinical practice. Further studies are required to validate these observations.

## Supplementary Information

Below is the link to the electronic supplementary material.

Supplementary Material 1

## Data Availability

Raw data from FAERS and JADER databases can be downloaded at https://fis.fda.gov/extensions/FPD-QDE-FAERS/FPD-QDE-FAERS.html and https://www.info.pmda.go.jp/fukusayoudb/CsvDownload.jsp; further inquiries can be directed to the corresponding author.

## Abbreviations

FAERS: Food and Drug Administration Adverse Event Reporting System
T2D: Type 2 diabetes
GLP-1: Glucagon like peptide-1
GIP: Glucose dependent insulin-promoting peptide
FDA: Food and Drug Administration
PS: Primary suspected
PT: Preferred terms
ROR: Reporting odds ratio
PRR: Proportional reporting ratio
BCPNN: Bayesian confidence propagation neural network
MGPS: Multi-item gamma Poisson shrinker
SOC: System organ class

## Declarations

### Author statement

All authors confirm that this manuscript is our original work and has not been published nor has it been submitted simultaneously elsewhere. All authors have checked the manuscript and have agreed to the submission.
