# Supplementary material for: Adverse events associated with tirzepatide: a focus on subgroup-specific differences
Source: BMC Pharmacol Toxicol. 2026 Feb 26;27:53. doi: 10.1186/s40360-026-01105-3 (PMC13041219; doi:10.1186/s40360-026-01105-3)
Supplement: Supplementary file 1 — Supplementary Material 1 [file 40360_2026_1105_MOESM1_ESM.docx]

# Supplementary Material

## Supplementary Table S1. Fourfold table of disproportionality analysis for tirzepatide signal detection.

|  | Target adverse event | Other adverse events | Total |
| --- | --- | --- | --- |
| Tirzepatide | a | b | a+b |
| All other drugs | c | d | c+d |
| Total | a+c | b+d | N=a+b+c+d |

For specific details, please refer to the references cited in the Method section of the manuscript.

## Supplementary Table S2. Equations and criteria of four algorithms for tirzepatide signal detection.

| Algorithms | Equation | Criteria |
| --- | --- | --- |
| ROR | ROR=(ad)/(bc) | Lower limit of 95% CI>1, N≥2 |
|  | Lower limit of 95% CI=e^ln(ROR)-1.96(1/a+1/b+1/c+1/d)^0.5^  Upper limit of 95% CI=e^ln(ROR)+1.96(1/a+1/b+1/c+1/d)^0.5^ |  |
| PRR | PRR=a(c+d)/c/(a+b) | PRR≥2, χ2≥4, N≥3 |
|  | χ2=[(ad-bc)^2](a+b+c+d)/[(a+b)(c+d)(a+c)(b+d)] |  |
| BCPNN | IC=log_2_a(a+b+c+d)/(a+c)/(a+b) | IC025>0 |
|  | IC025=E(IC)-2V(IC)^0.5 |  |
| MGPS | EBGM=a(a+b+c+d)/(a+c)/(a+b) | EBGM05>2 |
|  | EBGM05=e^ln(EBGM)-1.96(1/a+1/b+1/c+1/d)^0.5^ |  |

**Abbreviations:** ROR, reporting odds ratio; CI, confidence interval; PRR, proportional reporting ratio; χ2, chi-squared; BCPNN, Bayesian confidence propagation neural network; IC, information component; IC025, lower limit of 95% confidence interval of IC; E(IC), IC expectation; V(IC), variance of IC; MGPS, multi-item gamma Poisson shrinker; EBGM, empirical Bayesian geometric mean; EBGM05, lower limit of 95% confidence interval of EBGM. For specific details, please refer to the references cited in the Method section of the manuscript.

## Supplementary Table S3. Fourfold table of disproportionality analysis for difference detection of tirzepatide signals.

|  | Target adverse event with tirzepatide | Other adverse events with tirzepatide | Total |
| --- | --- | --- | --- |
| Factor 1 | a | b | a+b |
| Factor 2 | c | d | c+d |
| Total | a+c | b+d | N=a+b+c+d |

For specific details, please refer to the references cited in the Method section of the manuscript.

## Supplementary Table S4. Criteria of ROR and Chi-Square Test/Fisher’s exact Test for difference detection of tirzepatide signals.

|  | ROR | Chi-Square Test/Fisher’s exact Test |
| --- | --- | --- |
| Factor 1 more frequently to report | ROR>1, Lower limit of 95% CI>1,  N≥2 | P<0.05 |
| Factor 2 more frequently to report | ROR<1, Upper limit of 95% CI<1,  N≥2 | P<0.05 |

**Abbreviations:** ROR, reporting odds ratio; 95% CI, 95% confidence interval. When N is greater than or equal to 40 and a, b, c, and d are all greater than or equal to 5, Chi-Square Test is used. When N is less than or equal to 40 or one of a, b, c, d is less than 5, the Fisher’s exact Test is applied. For specific details, please refer to the references cited in the Method section of the manuscript.

## Supplementary Table S5. Number and signal strength of tirzepatide at the SOC level from the FAERS database.

| **SOC** | **Number** | **ROR (95% CI)** | **PRR (χ2)** | **IC (IC025)** | **EBGM (EBGM05)** |
| --- | --- | --- | --- | --- | --- |
| Injury, poisoning and procedural complications ^abcd^ | 28406 | 2.72 (2.68-2.76) | 2.22 (21493.59) | 1.13 (1.11) | 2.19 (2.16) |
| General disorders and administration site conditions ^ac^ | 25568 | 1.67 (1.65-1.69) | 1.49 (5003.97) | 0.57 (0.55) | 1.49 (1.47) |
| Gastrointestinal disorders ^abcd^ | 18482 | 2.75 (2.70-2.79) | 2.41 (16285.19) | 1.25 (1.23) | 2.38 (2.35) |
| Investigations | 5370 | 0.94 (0.91-0.96) | 0.94 (21.46) | -0.09 (-0.13) | 0.94 (0.92) |
| Nervous system disorders | 3383 | 0.46 (0.45-0.48) | 0.48 (2009.91) | -1.04 (-1.09) | 0.49 (0.47) |
| Metabolism and nutrition disorders ^ac^ | 3257 | 1.78 (1.72-1.85) | 1.76 (1069.32) | 0.80 (0.75) | 1.75 (1.69) |
| Skin and subcutaneous tissue disorders | 1849 | 0.35 (0.33-0.37) | 0.36 (2181.23) | -1.46 (-1.52) | 0.36 (0.35) |
| Psychiatric disorders | 1762 | 0.37 (0.35-0.39) | 0.38 (1829.90) | -1.38 (-1.45) | 0.39 (0.37) |
| Musculoskeletal and connective tissue disorders | 1531 | 0.29 (0.28-0.31) | 0.30 (2602.75) | -1.72 (-1.79) | 0.30 (0.29) |
| Infections and infestations | 1344 | 0.21 (0.20-0.23) | 0.23 (3806.03) | -2.14 (-2.22) | 0.23 (0.21) |
| Eye disorders | 920 | 0.46 (0.43-0.49) | 0.47 (574.38) | -1.10 (-1.19) | 0.47 (0.44) |
| Respiratory, thoracic and mediastinal disorders | 879 | 0.19 (0.18-0.20) | 0.20 (3020.29) | -2.34 (-2.43) | 0.20 (0.19) |
| Renal and urinary disorders | 611 | 0.39 (0.36-0.42) | 0.39 (586.47) | -1.35 (-1.46) | 0.39 (0.36) |
| Cardiac disorders | 502 | 0.27 (0.25-0.30) | 0.27 (979.97) | -1.86 (-1.98) | 0.28 (0.25) |
| Hepatobiliary disorders | 480 | 0.57 (0.52-0.63) | 0.58 (151.08) | -0.79 (-0.92) | 0.58 (0.53) |
| Immune system disorders | 475 | 0.42 (0.39-0.46) | 0.43 (371.14) | -1.23 (-1.36) | 0.43 (0.39) |
| Vascular disorders | 392 | 0.22 (0.20-0.24) | 0.22 (1099.96) | -2.17 (-2.32) | 0.22 (0.20) |
| Reproductive system and breast disorders | 345 | 0.61 (0.55-0.68) | 0.61 (84.18) | -0.70 (-0.85) | 0.62 (0.55) |
| Neoplasms benign, malignant and unspecified (incl cysts and polyps) | 268 | 0.10 (0.08-0.11) | 0.10 (2288.49) | -3.34 (-3.51) | 0.10 (0.09) |
| Product issues | 246 | 0.12 (0.11-0.14) | 0.12 (1554.42) | -3.00 (-3.18) | 0.12 (0.11) |
| Ear and labyrinth disorders | 212 | 0.53 (0.46-0.61) | 0.53 (88.40) | -0.91 (-1.11) | 0.53 (0.46) |
| Blood and lymphatic system disorders | 140 | 0.08 (0.07-0.10) | 0.08 (1442.25) | -3.58 (-3.81) | 0.08 (0.07) |
| Endocrine disorders | 120 | 0.43 (0.36-0.52) | 0.43 (88.83) | -1.20 (-1.46) | 0.44 (0.36) |
| Surgical and medical procedures | 81 | 0.05 (0.04-0.06) | 0.05 (1480.83) | -4.31 (-4.61) | 0.05 (0.04) |
| Social circumstances | 60 | 0.13 (0.10-0.17) | 0.13 (350.65) | -2.93 (-3.28) | 0.13 (0.10) |
| Pregnancy, puerperium and perinatal conditions | 38 | 0.12 (0.09-0.17) | 0.13 (232.72) | -2.99 (-3.42) | 0.13 (0.09) |
| Congenital, familial and genetic disorders | 9 | 0.04 (0.02-0.09) | 0.05 (182.38) | -4.46 (-5.23) | 0.05 (0.02) |

^a^: SOCs met the criteria of ROR algorithm. ^b^: SOCs met the criteria of PRR algorithm. ^c^: SOCs met the criteria of BCPNN algorithm. ^d^: SOCs met the criteria of MGPS algorithm. **Abbreviations:** SOC, system organ class; ROR, reporting odds ratio; CI, confidence interval; PRR, proportional reporting ratio; χ2, chi-squared; IC, information component; IC025, lower limit of 95% confidence interval of IC; EBGM, empirical Bayesian geometric mean; EBGM05, lower limit of 95% confidence interval of EBGM.

## Supplementary Table S6. Number and signal strength of 34 tirzepatide-unrelated signals at the PT level from the FAERS database.

| **PT** | **Number** | **ROR (95% CI)** | **PRR (χ2)** | **IC (IC025)** | **EBGM (EBGM05)** |  |
| --- | --- | --- | --- | --- | --- | --- |
| **Product issues** | | | | | | |
| Product tampering | 161 | 74.55 (61.19-90.83) | 74.43 (7138.49) | 5.52 (4.90) | 45.94 (37.71) |  |
| Product after taste | 16 | 4.02 (2.44-6.62) | 4.02 (35.14) | 1.97 (1.03) | 3.92 (2.38) |  |
| Suspected product tampering | 14 | 9.61 (5.58-16.58) | 9.61 (99.87) | 3.16 (1.77) | 8.96 (5.20) |  |
| Product compounding quality issue | 3 | 12.58 (3.82-41.38) | 12.58 (28.89) | 3.52 (0.12) | 11.46 (3.48) |  |
| **Injury, poisoning and procedural complications** | | | | | | |
| Incorrect dose administered | 12241 | 42.87 (41.96-43.80) | 37.57 (331589.50) | 4.84 (4.81) | 28.71 (28.10) |  |
| Off label use | 5259 | 2.65 (2.58-2.72) | 2.56 (4998.09) | 1.34 (1.30) | 2.53 (2.46) |  |
| Extra dose administered | 3341 | 58.15 (55.78-60.63) | 56.18 (122583.62) | 5.26 (5.19) | 38.32 (36.76) |  |
| Accidental underdose | 1786 | 80.54 (75.83-85.55) | 79.08 (82304.95) | 5.57 (5.46) | 47.66 (44.87) |  |
| Accidental overdose | 552 | 13.47 (12.33-14.71) | 13.40 (5687.79) | 3.60 (3.44) | 12.13 (11.10) |  |
| Product administered at inappropriate site | 540 | 12.80 (11.71-14.00) | 12.74 (5271.48) | 3.53 (3.38) | 11.59 (10.60) |  |
| Wrong patient received product | 47 | 6.78 (5.05-9.10) | 6.78 (218.95) | 2.69 (2.11) | 6.46 (4.82) |  |
| Counterfeit product administered | 18 | 5.90 (3.68-9.48) | 5.90 (69.81) | 2.50 (1.50) | 5.67 (3.53) |  |
| Intercepted product selection error | 7 | 7.83 (3.64-16.83) | 7.83 (39.08) | 2.89 (0.98) | 7.40 (3.44) |  |
| Foreign body | 4 | 6.91 (2.52-18.93) | 6.91 (19.08) | 2.72 (0.30) | 6.58 (2.40) |  |
| Hypobarism | 3 | 11.01 (3.37-35.95) | 11.01 (24.96) | 3.34 (0.10) | 10.15 (3.11) |  |
| **Consistent with indications** | | | | | | |
| Blood glucose increased | 1274 | 6.70 (6.33-7.10) | 6.63 (5776.85) | 2.66 (2.57) | 6.33 (5.98) |  |
| Decreased appetite | 1174 | 3.21 (3.03-3.41) | 3.19 (1721.25) | 1.65 (1.56) | 3.13 (2.95) |  |
| Weight decreased | 956 | 2.21 (2.07-2.36) | 2.20 (614.47) | 1.12 (1.02) | 2.17 (2.04) |  |
| Abdominal pain upper | 882 | 3.05 (2.85-3.26) | 3.03 (1171.17) | 1.57 (1.47) | 2.98 (2.78) |  |
| Dyspepsia | 616 | 4.62 (4.26-5.01) | 4.60 (1670.68) | 2.16 (2.03) | 4.46 (4.12) |  |
| Hunger | 496 | 29.13 (26.40-32.15) | 28.99 (10752.16) | 4.55 (4.35) | 23.45 (21.25) |  |
| Gastrointestinal disorder | 492 | 2.45 (2.24-2.68) | 2.44 (409.99) | 1.27 (1.13) | 2.41 (2.20) |  |
| Blood glucose decreased | 478 | 10.88 (9.91-11.95) | 10.83 (3907.89) | 3.32 (3.16) | 10.00 (9.11) |  |
| Abdominal distension | 446 | 3.07 (2.79-3.37) | 3.06 (603.11) | 1.59 (1.44) | 3.01 (2.74) |  |
| Gastrooesophageal reflux disease | 344 | 3.22 (2.89-3.59) | 3.21 (511.06) | 1.66 (1.49) | 3.15 (2.83) |  |
| Increased appetite | 323 | 12.56 (11.20-14.09) | 12.52 (3095.04) | 3.51 (3.30) | 11.41 (10.17) |  |
| Glycosylated haemoglobin increased | 318 | 8.67 (7.74-9.72) | 8.65 (2004.46) | 3.02 (2.82) | 8.12 (7.25) |  |
| Hypoglycaemia | 249 | 5.16 (4.55-5.86) | 5.15 (798.35) | 2.32 (2.11) | 4.98 (4.38) |  |
| Blood glucose abnormal | 149 | 5.96 (5.05-7.02) | 5.95 (583.99) | 2.51 (2.23) | 5.71 (4.84) |  |
| Blood glucose fluctuation | 62 | 5.53 (4.29-7.14) | 5.53 (219.62) | 2.41 (1.94) | 5.32 (4.13) |  |
| Glycosylated haemoglobin decreased | 31 | 10.52 (7.29-15.20) | 10.52 (245.12) | 3.28 (2.40) | 9.74 (6.74) |  |
| Glycosylated haemoglobin abnormal | 30 | 8.04 (5.56-11.65) | 8.04 (173.15) | 2.92 (2.11) | 7.59 (5.24) |  |
| Diabetic retinopathy | 20 | 5.41 (3.46-8.47) | 5.41 (68.75) | 2.38 (1.47) | 5.22 (3.33) |  |
| Blood insulin increased | 8 | 9.49 (4.62-19.50) | 9.49 (56.21) | 3.15 (1.23) | 8.85 (4.31) |  |

**Abbreviations:** PT, preferred term; ROR, reporting odds ratio; CI, confidence interval; PRR, proportional reporting ratio; χ2, chi-squared; IC, information component; IC025, lower limit of 95% confidence interval of IC; EBGM, empirical Bayesian geometric mean; EBGM05, lower limit of 95% confidence interval of EBGM.

## Supplementary Table S7. Number and signal strength of 84 tirzepatide-related signals at the PT level from the FAERS database.

| **PT** | **Number** | **ROR (95% CI)** | **PRR (χ2)** | **IC (IC025)** | **EBGM (EBGM05)** |
| --- | --- | --- | --- | --- | --- |
| **General disorders and administration site conditions** | | | | | |
| Injection site pain | 6398 | 16.15 (15.72-16.59) | 15.14 (75231.28) | 3.76 (3.72) | 13.53 (13.17) |
| Injection site haemorrhage | 2789 | 26.83 (25.74-27.97) | 26.09 (55129.46) | 4.43 (4.36) | 21.53 (20.65) |
| Injection site erythema | 2214 | 17.76 (16.97-18.58) | 17.37 (29804.14) | 3.93 (3.86) | 15.26 (14.59) |
| Injection site bruising | 1410 | 14.69 (13.90-15.53) | 14.49 (15783.26) | 3.70 (3.61) | 13.01 (12.31) |
| Injection site pruritus | 1130 | 15.00 (14.10-15.97) | 14.84 (12960.04) | 3.73 (3.63) | 13.29 (12.49) |
| Injection site mass | 783 | 14.25 (13.23-15.35) | 14.14 (8538.84) | 3.67 (3.54) | 12.73 (11.82) |
| Injection site swelling | 640 | 6.04 (5.57-6.54) | 6.00 (2541.22) | 2.53 (2.40) | 5.76 (5.32) |
| Injection site rash | 640 | 15.10 (13.91-16.40) | 15.01 (7424.90) | 3.75 (3.60) | 13.42 (12.36) |
| Injection site injury | 617 | 87.52 (78.85-97.14) | 86.97 (30126.50) | 5.66 (5.40) | 50.39 (45.40) |
| Injection site urticaria | 517 | 14.49 (13.22-15.88) | 14.42 (5752.84) | 3.70 (3.53) | 12.95 (11.82) |
| Injury associated with device | 472 | 11.27 (10.25-12.39) | 11.22 (4011.24) | 3.37 (3.20) | 10.33 (9.39) |
| Injection site reaction | 406 | 5.49 (4.97-6.06) | 5.47 (1417.28) | 2.40 (2.24) | 5.27 (4.77) |
| Injection site irritation | 192 | 16.64 (14.31-19.36) | 16.61 (2468.45) | 3.88 (3.56) | 14.68 (12.62) |
| Injection site discomfort | 170 | 10.17 (8.69-11.90) | 10.15 (1291.38) | 3.24 (2.94) | 9.42 (8.06) |
| Injection site warmth | 146 | 8.76 (7.40-10.36) | 8.75 (932.41) | 3.04 (2.72) | 8.21 (6.94) |
| Injection site coldness | 114 | 154.04 (116.53-203.63) | 153.86 (7493.66) | 6.07 (5.07) | 67.16 (50.81) |
| Injection site hypersensitivity | 100 | 21.22 (17.14-26.25) | 21.19 (1630.06) | 4.18 (3.64) | 18.11 (14.63) |
| Injection site vesicles | 84 | 8.14 (6.52-10.15) | 8.13 (491.38) | 2.94 (2.51) | 7.67 (6.15) |
| Injection site paraesthesia | 77 | 33.14 (25.74-42.68) | 33.12 (1870.82) | 4.70 (3.94) | 26.05 (20.23) |
| Injection site discolouration | 69 | 4.37 (3.44-5.56) | 4.37 (173.06) | 2.09 (1.67) | 4.25 (3.34) |
| Injection site inflammation | 59 | 8.26 (6.34-10.76) | 8.26 (351.62) | 2.96 (2.42) | 7.78 (5.97) |
| Injection site induration | 56 | 4.62 (3.53-6.03) | 4.61 (152.56) | 2.16 (1.69) | 4.48 (3.43) |
| Injection site indentation | 34 | 7.37 (5.21-10.42) | 7.37 (176.02) | 2.81 (2.07) | 6.99 (4.94) |
| Injection site scar | 29 | 6.78 (4.67-9.87) | 6.78 (135.18) | 2.69 (1.91) | 6.47 (4.45) |
| Injection site hypoaesthesia | 22 | 8.97 (5.82-13.84) | 8.97 (144.73) | 3.07 (2.04) | 8.40 (5.45) |
| Injection site laceration | 17 | 31.69 (18.55-54.15) | 31.68 (397.82) | 4.65 (2.67) | 25.16 (14.73) |
| Injection site scab | 14 | 12.55 (7.23-21.78) | 12.55 (134.43) | 3.52 (1.97) | 11.43 (6.59) |
| Thirst decreased | 12 | 13.82 (7.60-25.13) | 13.81 (127.62) | 3.64 (1.88) | 12.47 (6.85) |
| Injection site thrombosis | 6 | 13.29 (5.71-30.92) | 13.29 (61.27) | 3.59 (1.06) | 12.04 (5.18) |
| Application site wound | 3 | 8.81 (2.72-28.47) | 8.81 (19.31) | 3.05 (0.04) | 8.26 (2.56) |
| Injection site dermatitis | 3 | 17.61 (5.23-59.27) | 17.61 (40.88) | 3.95 (0.18) | 15.45 (4.59) |
| **Gastrointestinal disorders** | | | | | |
| Nausea | 4999 | 4.86 (4.72-5.01) | 4.66 (13993.41) | 2.18 (2.13) | 4.52 (4.39) |
| Diarrhoea | 2362 | 2.35 (2.25-2.45) | 2.32 (1750.48) | 1.20 (1.13) | 2.29 (2.20) |
| Vomiting | 2015 | 3.26 (3.12-3.41) | 3.21 (3007.48) | 1.66 (1.59) | 3.15 (3.02) |
| Constipation | 1483 | 4.53 (4.30-4.78) | 4.48 (3872.23) | 2.12 (2.04) | 4.35 (4.13) |
| Eructation | 841 | 41.27 (38.15-44.66) | 40.92 (24296.63) | 4.94 (4.77) | 30.61 (28.29) |
| Abdominal discomfort | 706 | 2.54 (2.36-2.74) | 2.53 (640.96) | 1.32 (1.21) | 2.50 (2.32) |
| Flatulence | 453 | 6.10 (5.55-6.71) | 6.08 (1829.96) | 2.54 (2.39) | 5.83 (5.30) |
| Pancreatitis | 425 | 7.84 (7.10-8.65) | 7.81 (2367.51) | 2.88 (2.72) | 7.38 (6.69) |
| Impaired gastric emptying | 322 | 19.85 (17.63-22.34) | 19.78 (4915.80) | 4.09 (3.85) | 17.08 (15.17) |
| Gastrointestinal sounds abnormal | 49 | 5.98 (4.49-7.96) | 5.97 (193.15) | 2.52 (1.97) | 5.73 (4.30) |
| Small intestinal obstruction | 43 | 2.78 (2.05-3.76) | 2.78 (47.84) | 1.45 (0.96) | 2.74 (2.02) |
| Ileus | 41 | 2.89 (2.12-3.93) | 2.88 (49.27) | 1.51 (0.99) | 2.84 (2.08) |
| Food poisoning | 37 | 3.60 (2.59-4.99) | 3.60 (67.30) | 1.82 (1.25) | 3.52 (2.54) |
| Vomiting projectile | 30 | 8.25 (5.70-11.95) | 8.25 (178.58) | 2.96 (2.14) | 7.77 (5.37) |
| Abdominal rigidity | 13 | 3.59 (2.07-6.24) | 3.59 (23.59) | 1.81 (0.79) | 3.51 (2.02) |
| **Metabolism and nutrition disorders** | | | | | |
| Dehydration | 469 | 2.92 (2.67-3.20) | 2.91 (576.38) | 1.52 (1.38) | 2.87 (2.62) |
| Feeding disorder | 254 | 6.65 (5.86-7.55) | 6.64 (1151.70) | 2.66 (2.45) | 6.34 (5.58) |
| Food craving | 123 | 18.28 (15.12-22.11) | 18.26 (1736.64) | 3.99 (3.55) | 15.94 (13.18) |
| Hypophagia | 89 | 2.50 (2.03-3.09) | 2.50 (78.42) | 1.30 (0.97) | 2.47 (2.00) |
| Fluid intake reduced | 44 | 6.00 (4.43-8.12) | 5.99 (174.18) | 2.52 (1.94) | 5.75 (4.25) |
| Appetite disorder | 35 | 2.91 (2.08-4.07) | 2.91 (42.90) | 1.52 (0.96) | 2.87 (2.05) |
| Food aversion | 23 | 7.67 (5.03-11.70) | 7.67 (125.28) | 2.86 (1.92) | 7.26 (4.76) |
| Starvation ketoacidosis | 6 | 20.72 (8.70-49.36) | 20.72 (95.72) | 4.15 (1.20) | 17.76 (7.46) |
| Lack of satiety | 4 | 9.79 (3.53-27.14) | 9.78 (29.12) | 3.19 (0.44) | 9.11 (3.28) |
| **Hepatobiliary disorders** | | | | | |
| Cholelithiasis | 115 | 3.30 (2.74-3.97) | 3.29 (178.64) | 1.69 (1.39) | 3.23 (2.68) |
| Gallbladder disorder | 84 | 4.86 (3.90-6.04) | 4.85 (246.88) | 2.23 (1.85) | 4.70 (3.78) |
| Cholecystitis | 50 | 3.87 (2.92-5.13) | 3.87 (103.04) | 1.92 (1.43) | 3.78 (2.85) |
| Biliary colic | 15 | 3.63 (2.17-6.07) | 3.63 (27.75) | 1.83 (0.88) | 3.55 (2.13) |
| Cholangitis acute | 7 | 5.96 (2.79-12.73) | 5.96 (27.47) | 2.52 (0.79) | 5.72 (2.68) |
| **Investigations** | | | | | |
| Lipase increased | 80 | 9.97 (7.93-12.53) | 9.96 (594.55) | 3.21 (2.74) | 9.26 (7.37) |
| Amylase increased | 24 | 4.47 (2.97-6.72) | 4.47 (62.20) | 2.12 (1.35) | 4.34 (2.89) |
| Pancreatic enzymes increased | 20 | 11.03 (6.97-17.44) | 11.03 (166.67) | 3.35 (2.16) | 10.16 (6.43) |
| Gastric pH decreased | 13 | 9.60 (5.45-16.90) | 9.60 (92.59) | 3.16 (1.71) | 8.95 (5.08) |
| Body mass index decreased | 6 | 6.91 (3.03-15.74) | 6.91 (28.63) | 2.72 (0.74) | 6.58 (2.89) |
| Blood calcitonin increased | 5 | 24.46 (9.33-64.12) | 24.46 (93.12) | 4.35 (0.97) | 20.42 (7.79) |
| **Skin and subcutaneous tissue disorders** | | | | | |
| Sensitive skin | 60 | 3.74 (2.89-4.84) | 3.74 (116.72) | 1.87 (1.43) | 3.65 (2.83) |
| Skin sensitisation | 10 | 8.27 (4.35-15.70) | 8.27 (59.70) | 2.96 (1.36) | 7.79 (4.10) |
| Skin laxity | 7 | 7.41 (3.45-15.90) | 7.40 (36.48) | 2.81 (0.94) | 7.02 (3.27) |
| **Psychiatric disorders** | | | | | |
| Sleep disorder due to general medical condition, insomnia type | 73 | 4.29 (3.39-5.42) | 4.29 (177.45) | 2.06 (1.66) | 4.17 (3.30) |
| **Reproductive system and breast disorders** | | | | | |
| Menstrual disorder | 33 | 3.65 (2.58-5.16) | 3.65 (61.54) | 1.84 (1.23) | 3.57 (2.52) |
| Postmenopausal haemorrhage | 29 | 8.37 (5.74-12.20) | 8.37 (175.59) | 2.98 (2.13) | 7.88 (5.40) |
| Oligomenorrhoea | 11 | 6.73 (3.66-12.35) | 6.73 (50.73) | 2.68 (1.28) | 6.42 (3.49) |
| **Nervous system disorders** | | | | | |
| Allodynia | 27 | 12.01 (8.08-17.85) | 12.01 (247.21) | 3.46 (2.44) | 10.99 (7.39) |
| Hypoglycaemic unconsciousness | 11 | 10.01 (5.41-18.53) | 10.01 (82.22) | 3.22 (1.59) | 9.30 (5.03) |
| Wernicke's encephalopathy | 6 | 7.92 (3.46-18.10) | 7.92 (33.96) | 2.90 (0.82) | 7.48 (3.27) |
| **Endocrine disorders** | | | | | |
| Thyroid mass | 32 | 4.51 (3.17-6.41) | 4.51 (84.05) | 2.13 (1.48) | 4.38 (3.07) |
| **Neoplasms benign, malignant and unspecified** | | | | | |
| Papillary thyroid cancer | 12 | 4.94 (2.77-8.81) | 4.94 (36.23) | 2.26 (1.07) | 4.78 (2.69) |
| Benign neoplasm of thyroid gland | 8 | 8.39 (4.09-17.19) | 8.39 (48.58) | 2.98 (1.16) | 7.89 (3.85) |
| Medullary thyroid cancer | 8 | 16.20 (7.73-33.92) | 16.20 (100.23) | 3.84 (1.50) | 14.35 (6.85) |
| **Infections and infestations** | | | | | |
| Injection site infection | 19 | 5.17 (3.26-8.18) | 5.16 (61.11) | 2.32 (1.39) | 4.99 (3.15) |
| Injection site pustule | 7 | 11.91 (5.47-25.92) | 11.91 (63.53) | 3.45 (1.21) | 10.91 (5.01) |
| **Blood and lymphatic system disorders** | | | | | |
| Lymph node pain | 12 | 4.52 (2.54-8.04) | 4.52 (31.63) | 2.13 (0.98) | 4.39 (2.46) |
| **Immune system disorders** | | | | | |
| Allergy to metals | 5 | 5.39 (2.20-13.20) | 5.39 (17.08) | 2.38 (0.40) | 5.19 (2.12) |

**Abbreviations:** PT, preferred term; ROR, reporting odds ratio; CI, confidence interval; PRR, proportional reporting ratio; χ2, chi-squared; IC, information component; IC025, lower limit of 95% confidence interval of IC; EBGM, empirical Bayesian geometric mean; EBGM05, lower limit of 95% confidence interval of EBGM.

## Supplementary Table S8. Number and signal strength of tirzepatide at the SOC level from the JADER database.

| **SOC** | **Number** | **ROR (95% CI)** | **PRR (χ2)** | **IC (IC025)** | **EBGM (EBGM05)** |
| --- | --- | --- | --- | --- | --- |
| Gastrointestinal disorders (SOC: 10017947) ^abcd^ | 100 | 3.91 (3.11-4.91) | 3.16 (160.39) | 1.66 (1.30) | 3.15 (2.51) |
| Metabolism and nutrition disorders (SOC: 10027433) ^abcd^ | 99 | 7.12 (5.67-8.96) | 5.56 (386.15) | 2.47 (2.08) | 5.54 (4.40) |
| Hepatobiliary disorders (SOC: 10019805) ^abcd^ | 41 | 3.17 (2.29-4.38) | 2.94 (54.23) | 1.55 (1.01) | 2.93 (2.12) |
| Infections and infestations (SOC: 10021881) | 22 | 0.54 (0.35-0.84) | 0.57 (7.95) | -0.81 (-1.41) | 0.57 (0.37) |
| Nervous system disorders (SOC: 10029205) | 22 | 0.71 (0.46-1.09) | 0.72 (2.54) | -0.47 (-1.07) | 0.72 (0.47) |
| Investigations (SOC: 10022891) | 16 | 0.57 (0.35-0.94) | 0.59 (4.89) | -0.76 (-1.44) | 0.59 (0.36) |
| Neoplasms benign, malignant and unspecified (incl cysts and polyps) (SOC: 10029104) | 15 | 0.68 (0.40-1.13) | 0.69 (2.25) | -0.54 (-1.25) | 0.69 (0.41) |
| Renal and urinary disorders (SOC: 10038359) | 12 | 0.76 (0.43-1.35) | 0.77 (0.89) | -0.38 (-1.17) | 0.77 (0.43) |
| General disorders and administration site conditions (SOC: 10018065) | 10 | 0.38 (0.20-0.70) | 0.39 (10.09) | -1.35 (-2.15) | 0.39 (0.21) |
| Injury, poisoning and procedural complications (SOC: 10022117) | 7 | 0.61 (0.29-1.29) | 0.62 (1.68) | -0.69 (-1.65) | 0.62 (0.29) |
| Eye disorders (SOC: 10015919) | 6 | 1.19 (0.53-2.66) | 1.18 (0.17) | 0.24 (-0.90) | 1.18 (0.53) |
| Immune system disorders (SOC: 10021428) | 6 | 0.56 (0.25-1.26) | 0.57 (2.00) | -0.81 (-1.82) | 0.57 (0.25) |
| Cardiac disorders (SOC: 10007541) | 6 | 0.36 (0.16-0.81) | 0.37 (6.59) | -1.42 (-2.39) | 0.37 (0.17) |
| Skin and subcutaneous tissue disorders (SOC: 10040785) | 5 | 0.28 (0.11-0.67) | 0.29 (9.30) | -1.80 (-2.81) | 0.29 (0.12) |
| Respiratory, thoracic and mediastinal disorders (SOC: 10038738) | 5 | 0.17 (0.07-0.41) | 0.18 (19.82) | -2.46 (-3.44) | 0.18 (0.08) |
| Psychiatric disorders (SOC: 10037175) | 4 | 0.65 (0.24-1.75) | 0.66 (0.72) | -0.60 (-1.80) | 0.66 (0.25) |
| Blood and lymphatic system disorders (SOC: 10005329) | 4 | 0.15 (0.06-0.40) | 0.16 (19.27) | -2.66 (-3.70) | 0.16 (0.06) |
| Vascular disorders (SOC: 10047065) | 3 | 0.33 (0.10-1.02) | 0.33 (4.15) | -1.59 (-2.78) | 0.33 (0.11) |
| Musculoskeletal and connective tissue disorders (SOC: 10028395) | 2 | 0.22 (0.05-0.87) | 0.22 (5.68) | -2.19 (-3.43) | 0.22 (0.05) |
| Reproductive system and breast disorders (SOC: 10038604) | 1 | 0.70 (0.10-4.98) | 0.70 (0.13) | -0.51 (-2.33) | 0.70 (0.10) |
| Endocrine disorders (SOC: 10014698) | 1 | 0.06 (0.01-0.39) | 0.06 (16.11) | -4.11 (-5.24) | 0.06 (0.01) |
| Congenital, familial and genetic disorders (SOC: 10010331) | 1 | 1.13 (0.16-8.06) | 1.13 (0.02) | 0.18 (-1.96) | 1.13 (0.16) |

^a^: SOCs met the criteria of ROR algorithm. ^b^: SOCs met the criteria of PRR algorithm. ^c^: SOCs met the criteria of BCPNN algorithm. ^d^: SOCs met the criteria of MGPS algorithm. **Abbreviations:** SOC, system organ class; ROR, reporting odds ratio; CI, confidence interval; PRR, proportional reporting ratio; χ2, chi-squared; IC, information component; IC025, lower limit of 95% confidence interval of IC; EBGM, empirical Bayesian geometric mean; EBGM05, lower limit of 95% confidence interval of EBGM.

## Supplementary Table S9. Number and signal strength of tirzepatide-related signals at the PT level from the JADER database.

| **PT** | **Number** | **ROR (95% CI)** | **PRR (χ2)** | **IC (IC025)** | **EBGM (EBGM05)** |
| --- | --- | --- | --- | --- | --- |
| **Metabolism and nutrition disorders (SOC: 10027433)** | | | | | |
| Hypoglycaemia (PT: 10020993) | 25 | 27.51 (18.26-41.46) | 25.80 (582.59) | 4.65 (3.11) | 25.18 (16.71) |
| Decreased appetite (PT: 10061428) | 17 | 6.43 (3.95-10.47) | 6.19 (74.10) | 2.62 (1.56) | 6.16 (3.78) |
| Dehydration (PT: 10012174) | 13 | 14.57 (8.35-25.42) | 14.12 (156.60) | 3.80 (2.06) | 13.93 (7.99) |
| Diabetic ketoacidosis (PT: 10012671) | 8 | 10.46 (5.18-21.14) | 10.27 (66.36) | 3.35 (1.35) | 10.17 (5.03) |
| Euglycaemic diabetic ketoacidosis (PT: 10080061) | 6 | 19.01 (8.43-42.90) | 18.73 (98.95) | 4.20 (1.29) | 18.41 (8.16) |
| Ketoacidosis (PT: 10023379) | 5 | 27.28 (11.16-66.69) | 26.94 (121.68) | 4.71 (1.13) | 26.26 (10.74) |
| Hyperglycaemia (PT: 10020635) | 4 | 8.59 (3.19-23.10) | 8.51 (26.32) | 3.08 (0.46) | 8.45 (3.14) |
| **Gastrointestinal disorders (SOC: 10017947)** | | | | | |
| Vomiting (PT: 10047700) | 24 | 19.66 (12.96-29.82) | 18.50 (391.48) | 4.18 (2.83) | 18.19 (11.99) |
| Pancreatitis (PT: 10033645) | 13 | 18.33 (10.50-32.02) | 17.75 (202.30) | 4.13 (2.21) | 17.46 (10.00) |
| Nausea (PT: 10028813) | 8 | 4.65 (2.30-9.37) | 4.57 (22.32) | 2.19 (0.73) | 4.55 (2.26) |
| Pancreatitis acute (PT: 10033647) | 8 | 16.89 (8.34-34.22) | 16.57 (115.25) | 4.03 (1.61) | 16.31 (8.05) |
| Retching (PT: 10038776) | 8 | 4.61 (2.28-9.30) | 4.53 (22.04) | 2.18 (0.72) | 4.52 (2.24) |
| Ileus paralytic (PT: 10021333) | 5 | 15.80 (6.50-38.44) | 15.61 (67.38) | 3.94 (0.98) | 15.39 (6.32) |
| Pneumatosis intestinalis (PT: 10057030) | 3 | 9.77 (3.12-30.58) | 9.70 (23.20) | 3.27 (0.15) | 9.62 (3.07) |
| **Hepatobiliary disorders (SOC: 10019805)** | | | | | |
| Cholecystitis (PT: 10008612) | 11 | 29.75 (16.20-54.64) | 28.94 (288.64) | 4.82 (2.25) | 28.15 (15.33) |
| Cholelithiasis (PT: 10008629) | 7 | 63.21 (29.27-136.51) | 62.08 (396.28) | 5.87 (1.77) | 58.52 (27.10) |
| Cholecystitis acute (PT: 10008614) | 7 | 41.28 (19.26-88.47) | 40.55 (259.66) | 5.29 (1.71) | 39.01 (18.20) |
| Cholangitis acute (PT: 10008605) | 6 | 107.14 (45.92-249.96) | 105.50 (561.98) | 6.58 (1.56) | 95.54 (40.95) |
| **Investigations (SOC: 10022891)** | | | | | |
| Weight decreased (PT: 10047895) | 6 | 19.37 (8.58-43.72) | 19.09 (101.01) | 4.23 (1.29) | 18.75 (8.31) |
| **Nervous system disorders (SOC: 10029205)** | | | | | |
| Dizziness (PT: 10013573) | 4 | 5.50 (2.05-14.77) | 5.45 (14.50) | 2.44 (0.22) | 5.43 (2.02) |
| **Infections and infestations (SOC: 10021881)** | | | | | |
| Enteritis infectious (PT: 10058839) | 3 | 27.05 (8.56-85.50) | 26.85 (72.72) | 4.71 (0.37) | 26.17 (8.28) |
| **Respiratory, thoracic and mediastinal disorders (SOC: 10038738)** | | | | | |
| Asphyxia (PT: 10003497) | 3 | 28.85 (9.12-91.30) | 28.63 (77.81) | 4.80 (0.37) | 27.87 (8.81) |

**Abbreviations:** PT, preferred term; ROR, reporting odds ratio; CI, confidence interval; PRR, proportional reporting ratio; χ2, chi-squared; IC, information component; IC025, lower limit of 95% confidence interval of IC; EBGM, empirical Bayesian geometric mean; EBGM05, lower limit of 95% confidence interval of EBGM.

## Supplementary Table S10. Number and signal strength of tirzepatide-related signals at the PT level from the FAERS database stratified by professional reporter.

| **PT** | **Number** | **ROR (95% CI)** | **PRR (χ2)** | **IC (IC025)** | **EBGM (EBGM05)** |  |
| --- | --- | --- | --- | --- | --- | --- |
| **General disorders and administration site conditions (SOC: 10018065)** | | | | | | |
| Injection site pain (PT: 10022086) | 245 | 11.81 (10.38-13.43) | 11.38 (2299.88) | 3.49 (3.24) | 11.26 (9.90) |  |
| Injection site erythema (PT: 10022061) | 157 | 21.06 (17.94-24.72) | 20.55 (2861.58) | 4.33 (3.93) | 20.13 (17.16) |  |
| Injection site pruritus (PT: 10022093) | 99 | 23.56 (19.27-28.80) | 23.20 (2054.38) | 4.50 (3.93) | 22.67 (18.54) |  |
| Injection site haemorrhage (PT: 10022067) | 94 | 40.38 (32.80-49.71) | 39.78 (3413.05) | 5.26 (4.47) | 38.23 (31.06) |  |
| Injection site rash (PT: 10022094) | 88 | 29.85 (24.11-36.96) | 29.44 (2346.27) | 4.84 (4.13) | 28.59 (23.09) |  |
| Injection site swelling (PT: 10053425) | 63 | 8.72 (6.79-11.18) | 8.64 (422.05) | 3.10 (2.57) | 8.57 (6.68) |  |
| Injection site bruising (PT: 10022052) | 61 | 14.94 (11.59-19.26) | 14.80 (773.52) | 3.87 (3.21) | 14.59 (11.32) |  |
| Injection site reaction (PT: 10022095) | 46 | 9.35 (6.98-12.51) | 9.28 (336.99) | 3.20 (2.55) | 9.20 (6.88) |  |
| Injection site urticaria (PT: 10022107) | 41 | 18.09 (13.27-24.67) | 17.98 (645.55) | 4.14 (3.21) | 17.67 (12.96) |  |
| Injury associated with device (PT: 10069803) | 34 | 31.42 (22.31-44.26) | 31.25 (964.35) | 4.92 (3.55) | 30.30 (21.51) |  |
| Injection site mass (PT: 10022081) | 30 | 12.72 (8.86-18.25) | 12.66 (318.14) | 3.64 (2.67) | 12.51 (8.72) |  |
| Injection site injury (PT: 10066083) | 23 | 182.40 (116.70-285.07) | 181.72 (3473.44) | 7.26 (3.74) | 152.85 (97.80) |  |
| Injection site warmth (PT: 10022112) | 20 | 21.62 (13.87-33.70) | 21.56 (383.45) | 4.40 (2.79) | 21.10 (13.54) |  |
| Injection site irritation (PT: 10022079) | 17 | 22.32 (13.79-36.13) | 22.26 (337.41) | 4.44 (2.65) | 21.78 (13.46) |  |
| Injection site hypersensitivity (PT: 10022071) | 15 | 53.84 (31.98-90.62) | 53.71 (734.65) | 5.67 (2.88) | 50.90 (30.24) |  |
| Injection site discomfort (PT: 10054266) | 6 | 7.14 (3.20-15.95) | 7.13 (31.42) | 2.83 (0.83) | 7.09 (3.17) |  |
| Injection site discolouration (PT: 10051572) | 5 | 6.24 (2.59-15.03) | 6.23 (21.82) | 2.63 (0.55) | 6.20 (2.57) |  |
| Injection site inflammation (PT: 10022078) | 5 | 10.77 (4.46-26.02) | 10.77 (43.80) | 3.41 (0.84) | 10.66 (4.41) |  |
| Injection site induration (PT: 10022075) | 4 | 5.94 (2.22-15.88) | 5.94 (16.33) | 2.56 (0.28) | 5.91 (2.21) |  |
| **Gastrointestinal disorders (SOC: 10017947)** | | | | | | |
| Nausea (PT: 10028813) | 316 | 5.34 (4.77-5.99) | 5.12 (1052.87) | 2.35 (2.16) | 5.10 (4.55) |  |
| Vomiting (PT: 10047700) | 186 | 4.81 (4.16-5.57) | 4.70 (541.99) | 2.23 (1.98) | 4.68 (4.04) |  |
| Diarrhoea (PT: 10012735) | 163 | 2.94 (2.52-3.44) | 2.89 (202.57) | 1.53 (1.28) | 2.88 (2.47) |  |
| Constipation (PT: 10010774) | 73 | 4.73 (3.75-5.96) | 4.69 (211.22) | 2.22 (1.81) | 4.67 (3.70) |  |
| Pancreatitis (PT: 10033645) | 39 | 9.33 (6.80-12.80) | 9.27 (285.25) | 3.20 (2.47) | 9.19 (6.70) |  |
| Eructation (PT: 10015137) | 28 | 45.11 (30.86-65.96) | 44.91 (1148.33) | 5.42 (3.58) | 42.94 (29.37) |  |
| Impaired gastric emptying (PT: 10021518) | 25 | 32.21 (21.60-48.01) | 32.08 (728.43) | 4.96 (3.27) | 31.07 (20.84) |  |
| Flatulence (PT: 10016766) | 15 | 5.69 (3.42-9.46) | 5.68 (57.48) | 2.50 (1.40) | 5.65 (3.40) |  |
| Gastritis (PT: 10017853) | 12 | 5.98 (3.39-10.55) | 5.97 (49.34) | 2.57 (1.30) | 5.94 (3.36) |  |
| Pancreatitis acute (PT: 10033647) | 11 | 3.80 (2.10-6.87) | 3.79 (22.54) | 1.92 (0.78) | 3.78 (2.09) |  |
| Small intestinal obstruction (PT: 10041101) | 8 | 7.24 (3.61-14.52) | 7.23 (42.62) | 2.84 (1.12) | 7.18 (3.58) |  |
| Ileus (PT: 10021328) | 7 | 5.20 (2.47-10.93) | 5.20 (23.59) | 2.37 (0.74) | 5.17 (2.46) |  |
| Pancreatitis necrotising (PT: 10033654) | 3 | 7.84 (2.52-24.43) | 7.84 (17.75) | 2.96 (0.08) | 7.78 (2.50) |  |
| **Metabolism and nutrition disorders (SOC: 10027433)** | | | | | | |
| Dehydration (PT: 10012174) | 48 | 5.30 (3.99-7.05) | 5.27 (165.24) | 2.39 (1.86) | 5.24 (3.94) |  |
| Feeding disorder (PT: 10061148) | 19 | 12.18 (7.74-19.16) | 12.14 (191.87) | 3.59 (2.30) | 12.00 (7.63) |  |
| Diabetic ketoacidosis (PT: 10012671) | 14 | 5.09 (3.01-8.62) | 5.09 (45.72) | 2.34 (1.25) | 5.06 (2.99) |  |
| Hypophagia (PT: 10063743) | 13 | 6.77 (3.92-11.69) | 6.76 (63.39) | 2.75 (1.48) | 6.72 (3.89) |  |
| Ketoacidosis (PT: 10023379) | 8 | 9.45 (4.71-18.98) | 9.44 (59.80) | 3.23 (1.31) | 9.36 (4.66) |  |
| Food craving (PT: 10056465) | 8 | 39.68 (19.55-80.51) | 39.63 (289.23) | 5.25 (1.91) | 38.09 (18.77) |  |
| Euglycaemic diabetic ketoacidosis (PT: 10080061) | 7 | 6.53 (3.11-13.75) | 6.53 (32.56) | 2.70 (0.92) | 6.49 (3.09) |  |
| Acidosis (PT: 10000486) | 6 | 6.74 (3.02-15.05) | 6.73 (29.08) | 2.74 (0.79) | 6.69 (3.00) |  |
| Hypovolaemia (PT: 10021137) | 4 | 5.70 (2.13-15.24) | 5.70 (15.41) | 2.50 (0.26) | 5.67 (2.12) |  |
| Fluid intake reduced (PT: 10056291) | 3 | 8.41 (2.70-26.23) | 8.41 (19.42) | 3.06 (0.10) | 8.35 (2.68) |  |
| **Hepatobiliary disorders (SOC: 10019805)** | | | | | | |
| Cholelithiasis (PT: 10008629) | 14 | 6.56 (3.87-11.10) | 6.54 (65.34) | 2.70 (1.50) | 6.51 (3.84) |  |
| Gallbladder disorder (PT: 10017626) | 9 | 12.61 (6.53-24.36) | 12.60 (94.85) | 3.64 (1.62) | 12.45 (6.45) |  |
| Cholecystitis (PT: 10008612) | 7 | 5.96 (2.83-12.53) | 5.95 (28.68) | 2.57 (0.85) | 5.92 (2.82) |  |
| Acute cholecystitis necrotic (PT: 10090325) | 3 | 84.39 (25.91-274.85) | 84.35 (227.06) | 6.28 (0.43) | 77.59 (23.83) |  |
| **Investigations (SOC: 10022891)** | | | | | | |
| Lipase increased (PT: 10024574) | 10 | 12.56 (6.73-23.46) | 12.55 (104.89) | 3.63 (1.73) | 12.40 (6.64) |  |
| Glomerular filtration rate decreased (PT: 10018358) | 8 | 4.17 (2.08-8.35) | 4.16 (19.15) | 2.05 (0.65) | 4.15 (2.07) |  |
| Pancreatic enzymes increased (PT: 10061900) | 4 | 26.57 (9.84-71.79) | 26.56 (95.71) | 4.69 (0.80) | 25.86 (9.57) |  |
| Drug screen positive (PT: 10049177) | 3 | 12.37 (3.96-38.64) | 12.36 (30.93) | 3.61 (0.23) | 12.22 (3.91) |  |
| **Reproductive system and breast disorders (SOC: 10038604)** | | | | | | |
| Menstruation irregular (PT: 10027339) | 7 | 10.35 (4.91-21.81) | 10.34 (58.45) | 3.36 (1.22) | 10.24 (4.86) |  |
| Menstrual disorder (PT: 10027327) | 5 | 12.11 (5.01-29.27) | 12.10 (50.29) | 3.58 (0.89) | 11.96 (4.95) |  |
| Postmenopausal haemorrhage (PT: 10055870) | 5 | 35.17 (14.40-85.90) | 35.15 (160.00) | 5.08 (1.18) | 33.94 (13.90) |  |
| Oligomenorrhoea (PT: 10030295) | 3 | 34.57 (10.92-109.41) | 34.55 (94.34) | 5.06 (0.40) | 33.38 (10.55) |  |
| **Nervous system disorders (SOC: 10029205)** | | | | | | |
| Allodynia (PT: 10053552) | 6 | 54.16 (23.79-123.33) | 54.11 (296.03) | 5.68 (1.52) | 51.27 (22.51) |  |
| Hyperaesthesia (PT: 10020568) | 4 | 6.33 (2.37-16.94) | 6.33 (17.84) | 2.65 (0.32) | 6.30 (2.35) |  |
| Hypoglycaemic unconsciousness (PT: 10065981) | 3 | 73.57 (22.73-238.14) | 73.54 (199.32) | 6.09 (0.43) | 68.36 (21.12) |  |
| **Infections and infestations (SOC: 10021881)** | | | | | | |
| Appendicitis (PT: 10003011) | 6 | 5.30 (2.37-11.82) | 5.29 (20.77) | 2.40 (0.61) | 5.27 (2.36) |  |
| Injection site cellulitis (PT: 10050057) | 4 | 30.37 (11.22-82.20) | 30.35 (110.04) | 4.88 (0.82) | 29.45 (10.88) |  |
| **Eye disorders (SOC: 10015919)** | | | | | | |
| Retinopathy (PT: 10038923) | 4 | 11.09 (4.14-29.73) | 11.08 (36.28) | 3.46 (0.57) | 10.97 (4.09) |  |
| Macular oedema (PT: 10025415) | 4 | 7.30 (2.73-19.53) | 7.30 (21.58) | 2.86 (0.39) | 7.25 (2.71) |  |
| **Skin and subcutaneous tissue disorders (SOC: 10040785)** | | | | | | |
| Sensitive skin (PT: 10081765) | 5 | 8.99 (3.73-21.70) | 8.98 (35.15) | 3.16 (0.76) | 8.91 (3.69) |  |
| **Respiratory, thoracic and mediastinal disorders (SOC: 10038738)** | | | | | | |
| Hiccups (PT: 10020039) | 4 | 6.41 (2.40-17.14) | 6.41 (18.13) | 2.67 (0.32) | 6.37 (2.38) |  |

For both tirzepatide and all other drugs, only reports from professionals were included. **Abbreviations:** PT, preferred term; ROR, reporting odds ratio; CI, confidence interval; PRR, proportional reporting ratio; χ2, chi-squared; IC, information component; IC025, lower limit of 95% confidence interval of IC; EBGM, empirical Bayesian geometric mean; EBGM05, lower limit of 95% confidence interval of EBGM.

## Supplementary Table S11. Number and signal strength of tirzepatide-related signals at the PT level from the FAERS database stratified by non-professional reporter.

| **PT** | **Number** | **ROR (95% CI)** | **PRR (χ2)** | **IC (IC025)** | **EBGM (EBGM05)** |
| --- | --- | --- | --- | --- | --- |
| **General disorders and administration site conditions (SOC: 10018065)** | | | | | |
| Injection site pain (PT: 10022086) | 6153 | 13.44 (13.07-13.83) | 12.60 (54445.61) | 3.40 (3.36) | 10.55 (10.25) |
| Injection site haemorrhage (PT: 10022067) | 2695 | 15.88 (15.21-16.57) | 15.44 (28887.51) | 3.64 (3.57) | 12.43 (11.91) |
| Injection site erythema (PT: 10022061) | 2056 | 16.41 (15.63-17.24) | 16.06 (22851.85) | 3.68 (3.60) | 12.83 (12.22) |
| Injection site bruising (PT: 10022052) | 1349 | 10.87 (10.25-11.52) | 10.72 (10075.69) | 3.21 (3.11) | 9.22 (8.70) |
| Injection site pruritus (PT: 10022093) | 1029 | 13.03 (12.17-13.94) | 12.89 (9265.70) | 3.43 (3.31) | 10.75 (10.05) |
| Injection site mass (PT: 10022081) | 753 | 10.70 (9.90-11.57) | 10.62 (5562.71) | 3.19 (3.06) | 9.15 (8.46) |
| Injection site injury (PT: 10066083) | 594 | 49.45 (44.34-55.15) | 49.13 (15263.05) | 4.77 (4.56) | 27.22 (24.41) |
| Injection site swelling (PT: 10053425) | 577 | 6.30 (5.78-6.86) | 6.26 (2308.76) | 2.53 (2.39) | 5.76 (5.28) |
| Injection site rash (PT: 10022094) | 552 | 15.72 (14.31-17.27) | 15.63 (5974.51) | 3.65 (3.48) | 12.56 (11.43) |
| Injection site urticaria (PT: 10022107) | 475 | 13.99 (12.65-15.46) | 13.92 (4607.42) | 3.52 (3.34) | 11.45 (10.35) |
| Injury associated with device (PT: 10069803) | 438 | 6.65 (6.02-7.34) | 6.62 (1879.74) | 2.60 (2.44) | 6.05 (5.48) |
| Injection site reaction (PT: 10022095) | 360 | 5.46 (4.90-6.09) | 5.45 (1197.03) | 2.34 (2.17) | 5.07 (4.55) |
| Injection site irritation (PT: 10022079) | 175 | 16.85 (14.24-19.93) | 16.82 (2024.64) | 3.73 (3.39) | 13.30 (11.24) |
| Injection site discomfort (PT: 10054266) | 164 | 8.36 (7.09-9.84) | 8.34 (928.45) | 2.89 (2.60) | 7.43 (6.31) |
| Injection site warmth (PT: 10022112) | 126 | 7.13 (5.92-8.57) | 7.12 (591.16) | 2.69 (2.36) | 6.46 (5.37) |
| Injection site coldness (PT: 10050082) | 112 | 106.36 (77.99-145.07) | 106.23 (4160.27) | 5.27 (4.50) | 38.50 (28.23) |
| Injection site hypersensitivity (PT: 10022071) | 85 | 17.80 (13.97-22.70) | 17.79 (1034.11) | 3.80 (3.25) | 13.89 (10.90) |
| Injection site vesicles (PT: 10022111) | 81 | 8.67 (6.86-10.95) | 8.66 (478.48) | 2.94 (2.49) | 7.68 (6.08) |
| Injection site paraesthesia (PT: 10022088) | 75 | 24.12 (18.44-31.56) | 24.10 (1178.02) | 4.12 (3.46) | 17.39 (13.29) |
| Injection site discolouration (PT: 10051572) | 64 | 3.49 (2.71-4.49) | 3.49 (107.25) | 1.74 (1.32) | 3.35 (2.60) |
| Injection site inflammation (PT: 10022078) | 54 | 8.21 (6.18-10.92) | 8.21 (299.88) | 2.87 (2.30) | 7.32 (5.51) |
| Injection site induration (PT: 10022075) | 52 | 3.98 (3.01-5.28) | 3.98 (108.77) | 1.92 (1.44) | 3.79 (2.86) |
| Injection site indentation (PT: 10079277) | 34 | 4.59 (3.24-6.50) | 4.59 (88.45) | 2.11 (1.47) | 4.33 (3.05) |
| Injection site scar (PT: 10059009) | 29 | 4.94 (3.38-7.22) | 4.94 (84.15) | 2.21 (1.50) | 4.64 (3.17) |
| Injection site hypoaesthesia (PT: 10074586) | 21 | 7.35 (4.67-11.57) | 7.35 (102.43) | 2.73 (1.75) | 6.65 (4.22) |
| Injection site laceration (PT: 10067253) | 15 | 18.38 (10.29-32.82) | 18.38 (187.80) | 3.83 (2.14) | 14.24 (7.97) |
| Injection site haematoma (PT: 10022066) | 15 | 3.80 (2.26-6.41) | 3.80 (29.10) | 1.86 (0.89) | 3.63 (2.15) |
| Injection site scab (PT: 10066210) | 13 | 11.08 (6.13-20.04) | 11.08 (100.31) | 3.25 (1.72) | 9.48 (5.24) |
| Thirst decreased (PT: 10050200) | 11 | 8.09 (4.31-15.19) | 8.09 (60.05) | 2.85 (1.36) | 7.23 (3.85) |
| Injection site dryness (PT: 10067252) | 7 | 6.43 (2.95-14.04) | 6.43 (28.95) | 2.56 (0.78) | 5.90 (2.70) |
| Injection site thrombosis (PT: 10022104) | 5 | 12.78 (4.86-33.63) | 12.78 (44.61) | 3.42 (0.72) | 10.68 (4.06) |
| Injection site exfoliation (PT: 10068689) | 5 | 9.49 (3.69-24.40) | 9.49 (32.68) | 3.05 (0.62) | 8.31 (3.23) |
| **Gastrointestinal disorders (SOC: 10017947)** | | | | | |
| Nausea (PT: 10028813) | 4679 | 4.43 (4.30-4.57) | 4.25 (11004.95) | 2.01 (1.97) | 4.04 (3.91) |
| Vomiting (PT: 10047700) | 1826 | 3.16 (3.01-3.31) | 3.12 (2507.64) | 1.59 (1.52) | 3.01 (2.87) |
| Constipation (PT: 10010774) | 1407 | 3.55 (3.36-3.75) | 3.51 (2397.14) | 1.75 (1.67) | 3.37 (3.19) |
| Eructation (PT: 10015137) | 813 | 26.82 (24.68-29.15) | 26.59 (13795.10) | 4.22 (4.07) | 18.62 (17.14) |
| Flatulence (PT: 10016766) | 437 | 4.20 (3.81-4.63) | 4.19 (990.54) | 1.99 (1.84) | 3.97 (3.61) |
| Pancreatitis (PT: 10033645) | 386 | 10.14 (9.10-11.29) | 10.10 (2701.63) | 3.13 (2.95) | 8.76 (7.87) |
| Impaired gastric emptying (PT: 10021518) | 296 | 15.32 (13.48-17.41) | 15.27 (3134.35) | 3.62 (3.38) | 12.33 (10.85) |
| Gastrointestinal sounds abnormal (PT: 10067715) | 48 | 4.07 (3.04-5.45) | 4.07 (103.87) | 1.95 (1.44) | 3.87 (2.89) |
| Pancreatitis acute (PT: 10033647) | 48 | 6.90 (5.12-9.31) | 6.90 (216.81) | 2.65 (2.07) | 6.28 (4.66) |
| Ileus (PT: 10021328) | 33 | 5.39 (3.78-7.70) | 5.39 (108.11) | 2.33 (1.65) | 5.02 (3.52) |
| Vomiting projectile (PT: 10047708) | 27 | 5.57 (3.76-8.27) | 5.57 (92.51) | 2.37 (1.60) | 5.18 (3.49) |
| Ileus paralytic (PT: 10021333) | 9 | 5.82 (2.93-11.54) | 5.82 (32.66) | 2.43 (0.94) | 5.38 (2.71) |
| Pancreatitis necrotising (PT: 10033654) | 7 | 6.53 (2.99-14.27) | 6.53 (29.53) | 2.58 (0.79) | 5.98 (2.74) |
| Obstructive pancreatitis (PT: 10079822) | 4 | 6.36 (2.27-17.84) | 6.36 (16.30) | 2.54 (0.19) | 5.83 (2.08) |
| **Metabolism and nutrition disorders (SOC: 10027433)** | | | | | |
| Dehydration (PT: 10012174) | 420 | 2.59 (2.35-2.86) | 2.58 (391.43) | 1.33 (1.18) | 2.52 (2.28) |
| Feeding disorder (PT: 10061148) | 235 | 4.70 (4.12-5.37) | 4.69 (632.76) | 2.14 (1.93) | 4.42 (3.87) |
| Food craving (PT: 10056465) | 115 | 11.46 (9.38-13.99) | 11.44 (917.60) | 3.28 (2.89) | 9.74 (7.98) |
| Diabetic ketoacidosis (PT: 10012671) | 46 | 4.38 (3.25-5.91) | 4.38 (111.57) | 2.05 (1.52) | 4.14 (3.07) |
| Fluid intake reduced (PT: 10056291) | 41 | 4.80 (3.49-6.61) | 4.80 (114.15) | 2.18 (1.59) | 4.52 (3.28) |
| Food aversion (PT: 10049238) | 23 | 5.66 (3.69-8.68) | 5.66 (80.49) | 2.39 (1.54) | 5.25 (3.42) |
| Ketoacidosis (PT: 10023379) | 16 | 4.90 (2.94-8.16) | 4.90 (45.86) | 2.20 (1.19) | 4.60 (2.76) |
| Euglycaemic diabetic ketoacidosis (PT: 10080061) | 12 | 16.41 (8.66-31.13) | 16.41 (135.78) | 3.71 (1.86) | 13.05 (6.88) |
| Hypovolaemia (PT: 10021137) | 8 | 8.11 (3.87-16.99) | 8.11 (43.83) | 2.86 (1.06) | 7.25 (3.46) |
| Starvation ketoacidosis (PT: 10082528) | 4 | 78.41 (17.55-350.37) | 78.41 (131.01) | 5.09 (0.50) | 34.18 (7.65) |
| **Hepatobiliary disorders (SOC: 10019805)** | | | | | |
| Cholelithiasis (PT: 10008629) | 101 | 3.00 (2.45-3.66) | 3.00 (127.78) | 1.54 (1.22) | 2.90 (2.37) |
| Gallbladder disorder (PT: 10017626) | 75 | 3.43 (2.72-4.33) | 3.43 (122.01) | 1.72 (1.34) | 3.30 (2.61) |
| Cholecystitis (PT: 10008612) | 43 | 6.75 (4.92-9.25) | 6.74 (188.72) | 2.62 (2.00) | 6.15 (4.49) |
| Cholecystitis acute (PT: 10008614) | 12 | 9.54 (5.18-17.55) | 9.54 (78.90) | 3.06 (1.55) | 8.35 (4.53) |
| Cholangitis acute (PT: 10008605) | 7 | 34.31 (13.51-87.14) | 34.30 (142.95) | 4.46 (1.38) | 22.03 (8.67) |
| **Investigations (SOC: 10022891)** | | | | | |
| Lipase increased (PT: 10024574) | 70 | 24.67 (18.66-32.61) | 24.65 (1119.26) | 4.14 (3.44) | 17.66 (13.36) |
| Amylase increased (PT: 10002016) | 21 | 15.06 (9.33-24.33) | 15.06 (219.47) | 3.61 (2.33) | 12.19 (7.55) |
| Pancreatic enzymes increased (PT: 10061900) | 16 | 14.05 (8.14-24.23) | 14.04 (156.47) | 3.53 (2.05) | 11.53 (6.68) |
| Gastric pH decreased (PT: 10060041) | 13 | 7.21 (4.05-12.83) | 7.21 (61.96) | 2.71 (1.40) | 6.53 (3.67) |
| Body mass index decreased (PT: 10005895) | 6 | 8.61 (3.65-20.27) | 8.61 (35.18) | 2.93 (0.79) | 7.64 (3.24) |
| Blood folate decreased (PT: 10005527) | 5 | 6.00 (2.39-15.06) | 6.00 (18.91) | 2.47 (0.40) | 5.54 (2.21) |
| Blood calcitonin increased (PT: 10005390) | 5 | 24.50 (8.63-69.56) | 24.50 (79.57) | 4.14 (0.85) | 17.59 (6.20) |
| **Psychiatric disorders (SOC: 10037175)** | | | | | |
| Sleep disorder due to general medical condition, insomnia type (PT: 10040986) | 71 | 14.66 (11.30-19.02) | 14.65 (722.97) | 3.58 (3.00) | 11.93 (9.20) |
| **Nervous system disorders (SOC: 10029205)** | | | | | |
| Allodynia (PT: 10053552) | 21 | 8.02 (5.08-12.65) | 8.02 (113.54) | 2.84 (1.83) | 7.18 (4.55) |
| Hypoglycaemic unconsciousness (PT: 10065981) | 8 | 5.23 (2.54-10.77) | 5.23 (25.12) | 2.29 (0.76) | 4.88 (2.37) |
| Wernicke's encephalopathy (PT: 10047911) | 3 | 44.11 (9.87-197.08) | 44.11 (72.22) | 4.68 (0.05) | 25.63 (5.74) |
| **Reproductive system and breast disorders (SOC: 10038604)** | | | | | |
| Postmenopausal haemorrhage (PT: 10055870) | 24 | 5.35 (3.52-8.12) | 5.35 (77.74) | 2.32 (1.50) | 4.98 (3.28) |
| Oligomenorrhoea (PT: 10030295) | 8 | 4.44 (2.16-9.11) | 4.44 (19.81) | 2.07 (0.62) | 4.20 (2.05) |
| **Endocrine disorders (SOC: 10014698)** | | | | | |
| Thyroid mass (PT: 10058900) | 31 | 3.57 (2.48-5.13) | 3.57 (54.01) | 1.77 (1.14) | 3.42 (2.38) |
| **Infections and infestations (SOC: 10021881)** | | | | | |
| Injection site infection (PT: 10022076) | 17 | 4.78 (2.92-7.84) | 4.78 (47.05) | 2.17 (1.20) | 4.50 (2.74) |
| Injection site pustule (PT: 10054994) | 5 | 7.95 (3.12-20.22) | 7.95 (26.75) | 2.83 (0.55) | 7.12 (2.80) |
| **Skin and subcutaneous tissue disorders (SOC: 10040785)** | | | | | |
| Skin sensitisation (PT: 10070835) | 8 | 5.81 (2.81-12.01) | 5.81 (28.98) | 2.43 (0.84) | 5.38 (2.60) |
| **Immune system disorders (SOC: 10021428)** | | | | | |
| Allergy to metals (PT: 10066414) | 5 | 6.00 (2.39-15.06) | 6.00 (18.91) | 2.47 (0.40) | 5.54 (2.21) |
| **Respiratory, thoracic and mediastinal disorders (SOC: 10038738)** | | | | | |
| Pneumomediastinum (PT: 10050184) | 4 | 14.70 (4.92-43.98) | 14.70 (40.86) | 3.58 (0.45) | 11.96 (4.00) |

For both tirzepatide and all other drugs, only reports from non-professionals were included. **Abbreviations:** PT, preferred term; ROR, reporting odds ratio; CI, confidence interval; PRR, proportional reporting ratio; χ2, chi-squared; IC, information component; IC025, lower limit of 95% confidence interval of IC; EBGM, empirical Bayesian geometric mean; EBGM05, lower limit of 95% confidence interval of EBGM.

## Supplementary Table S12. Number and signal strength of tirzepatide-related signals at the PT level from the FAERS database stratified by males.

| **PT** | **Number** | **ROR (95% CI)** | **PRR (χ2)** | **IC (IC025)** | **EBGM (EBGM05)** |  |
| --- | --- | --- | --- | --- | --- | --- |
| **General disorders and administration site conditions (SOC: 10018065)** | | | | | | |
| Injection site pain (PT: 10022086) | 1086 | 13.94 (13.09-14.85) | 13.21 (11514.95) | 3.63 (3.53) | 12.42 (11.66) |  |
| Injection site haemorrhage (PT: 10022067) | 556 | 24.51 (22.42-26.81) | 23.83 (10829.44) | 4.41 (4.23) | 21.30 (19.48) |  |
| Injection site erythema (PT: 10022061) | 328 | 19.58 (17.46-21.96) | 19.26 (5164.86) | 4.14 (3.90) | 17.59 (15.69) |  |
| Injection site mass (PT: 10022081) | 204 | 14.87 (12.89-17.16) | 14.72 (2424.94) | 3.78 (3.48) | 13.74 (11.91) |  |
| Injection site bruising (PT: 10022052) | 173 | 14.09 (12.07-16.45) | 13.97 (1943.20) | 3.71 (3.39) | 13.09 (11.21) |  |
| Injection site injury (PT: 10066083) | 123 | 83.11 (67.23-102.72) | 82.58 (6924.95) | 5.86 (5.01) | 57.98 (46.91) |  |
| Injection site swelling (PT: 10053425) | 118 | 6.44 (5.36-7.74) | 6.41 (521.50) | 2.64 (2.31) | 6.23 (5.18) |  |
| Injection site pruritus (PT: 10022093) | 109 | 12.53 (10.32-15.22) | 12.47 (1079.76) | 3.56 (3.14) | 11.76 (9.69) |  |
| Injection site rash (PT: 10022094) | 65 | 11.45 (8.91-14.71) | 11.41 (582.79) | 3.44 (2.87) | 10.82 (8.42) |  |
| Injection site urticaria (PT: 10022107) | 62 | 13.29 (10.27-17.20) | 13.25 (657.13) | 3.64 (3.02) | 12.46 (9.63) |  |
| Injury associated with device (PT: 10069803) | 58 | 13.91 (10.65-18.17) | 13.87 (646.08) | 3.70 (3.04) | 13.00 (9.96) |  |
| Injection site discomfort (PT: 10054266) | 55 | 15.59 (11.84-20.52) | 15.54 (692.38) | 3.85 (3.14) | 14.45 (10.98) |  |
| Injection site reaction (PT: 10022095) | 49 | 4.94 (3.72-6.56) | 4.93 (149.70) | 2.27 (1.75) | 4.83 (3.64) |  |
| Injection site irritation (PT: 10022079) | 28 | 15.28 (10.40-22.47) | 15.26 (345.67) | 3.83 (2.73) | 14.21 (9.67) |  |
| Injection site coldness (PT: 10050082) | 24 | 143.68 (84.62-243.97) | 143.50 (1940.74) | 6.37 (3.58) | 82.43 (48.55) |  |
| Injection site paraesthesia (PT: 10022088) | 20 | 53.95 (32.84-88.63) | 53.90 (810.15) | 5.40 (3.13) | 42.27 (25.73) |  |
| Injection site vesicles (PT: 10022111) | 17 | 8.00 (4.92-13.00) | 7.99 (99.83) | 2.95 (1.79) | 7.71 (4.75) |  |
| Injection site warmth (PT: 10022112) | 16 | 8.39 (5.09-13.85) | 8.39 (99.74) | 3.01 (1.79) | 8.08 (4.90) |  |
| Injection site hypersensitivity (PT: 10022071) | 16 | 25.32 (15.03-42.66) | 25.30 (329.84) | 4.49 (2.57) | 22.46 (13.33) |  |
| Injection site discolouration (PT: 10051572) | 11 | 5.75 (3.16-10.48) | 5.75 (41.91) | 2.49 (1.17) | 5.61 (3.08) |  |
| Feeling jittery (PT: 10016338) | 10 | 3.93 (2.10-7.35) | 3.93 (21.40) | 1.95 (0.74) | 3.87 (2.07) |  |
| Injection site induration (PT: 10022075) | 9 | 4.10 (2.12-7.94) | 4.10 (20.66) | 2.01 (0.70) | 4.04 (2.08) |  |
| Injection site inflammation (PT: 10022078) | 9 | 8.66 (4.44-16.89) | 8.65 (58.29) | 3.06 (1.33) | 8.32 (4.27) |  |
| Injection site indentation (PT: 10079277) | 7 | 10.23 (4.78-21.88) | 10.22 (55.30) | 3.29 (1.17) | 9.76 (4.56) |  |
| Injection site scar (PT: 10059009) | 6 | 8.14 (3.60-18.44) | 8.14 (36.06) | 2.97 (0.87) | 7.85 (3.47) |  |
| Injection site laceration (PT: 10067253) | 5 | 50.37 (18.80-134.90) | 50.35 (191.47) | 5.32 (1.10) | 40.07 (14.96) |  |
| **Gastrointestinal disorders (SOC: 10017947)** | | | | | | |
| Nausea (PT: 10028813) | 795 | 5.58 (5.19-6.00) | 5.39 (2786.84) | 2.40 (2.28) | 5.27 (4.90) |  |
| Diarrhoea (PT: 10012735) | 634 | 3.34 (3.08-3.61) | 3.26 (985.65) | 1.69 (1.56) | 3.22 (2.97) |  |
| Vomiting (PT: 10047700) | 360 | 3.81 (3.43-4.23) | 3.75 (716.98) | 1.89 (1.72) | 3.70 (3.33) |  |
| Constipation (PT: 10010774) | 327 | 5.25 (4.70-5.86) | 5.18 (1076.73) | 2.34 (2.16) | 5.07 (4.54) |  |
| Eructation (PT: 10015137) | 201 | 55.04 (47.03-64.42) | 54.47 (8214.86) | 5.41 (4.92) | 42.62 (36.42) |  |
| Abdominal discomfort (PT: 10000059) | 188 | 4.46 (3.86-5.16) | 4.43 (488.61) | 2.12 (1.88) | 4.35 (3.76) |  |
| Flatulence (PT: 10016766) | 136 | 8.98 (7.56-10.67) | 8.92 (915.00) | 3.10 (2.77) | 8.57 (7.21) |  |
| Pancreatitis (PT: 10033645) | 98 | 8.04 (6.56-9.84) | 8.00 (576.51) | 2.95 (2.56) | 7.72 (6.30) |  |
| Impaired gastric emptying (PT: 10021518) | 39 | 21.86 (15.69-30.46) | 21.82 (695.51) | 4.30 (3.26) | 19.69 (14.13) |  |
| Small intestinal obstruction (PT: 10041101) | 24 | 6.73 (4.48-10.11) | 6.72 (112.98) | 2.71 (1.83) | 6.53 (4.34) |  |
| Bowel movement irregularity (PT: 10063541) | 14 | 3.94 (2.32-6.69) | 3.94 (30.09) | 1.96 (0.95) | 3.88 (2.29) |  |
| Gastrointestinal pain (PT: 10017999) | 12 | 3.66 (2.07-6.49) | 3.66 (22.79) | 1.85 (0.78) | 3.61 (2.04) |  |
| Food poisoning (PT: 10016952) | 11 | 5.34 (2.94-9.73) | 5.34 (37.77) | 2.39 (1.10) | 5.22 (2.87) |  |
| Gastrointestinal sounds abnormal (PT: 10067715) | 11 | 6.12 (3.36-11.16) | 6.12 (45.64) | 2.58 (1.23) | 5.96 (3.27) |  |
| Regurgitation (PT: 10067171) | 6 | 5.80 (2.57-13.07) | 5.80 (23.12) | 2.50 (0.65) | 5.66 (2.51) |  |
| Vomiting projectile (PT: 10047708) | 4 | 7.22 (2.66-19.60) | 7.22 (20.66) | 2.81 (0.35) | 6.99 (2.58) |  |
| **Metabolism and nutrition disorders (SOC: 10027433)** | | | | | | |
| Dehydration (PT: 10012174) | 113 | 3.38 (2.80-4.07) | 3.37 (184.93) | 1.73 (1.43) | 3.32 (2.76) |  |
| Feeding disorder (PT: 10061148) | 41 | 6.27 (4.59-8.56) | 6.26 (175.37) | 2.61 (1.99) | 6.09 (4.46) |  |
| Diabetic ketoacidosis (PT: 10012671) | 23 | 3.15 (2.08-4.75) | 3.15 (33.13) | 1.64 (0.92) | 3.11 (2.06) |  |
| Food craving (PT: 10056465) | 15 | 17.41 (10.26-29.54) | 17.39 (212.47) | 4.00 (2.29) | 16.03 (9.45) |  |
| Weight loss poor (PT: 10049084) | 5 | 6.79 (2.78-16.56) | 6.79 (23.82) | 2.72 (0.56) | 6.59 (2.70) |  |
| **Hepatobiliary disorders (SOC: 10019805)** | | | | | | |
| Cholelithiasis (PT: 10008629) | 28 | 4.37 (3.01-6.36) | 4.37 (71.16) | 2.10 (1.40) | 4.29 (2.95) |  |
| Gallbladder disorder (PT: 10017626) | 16 | 4.59 (2.80-7.54) | 4.59 (43.88) | 2.17 (1.19) | 4.51 (2.74) |  |
| Cholecystitis (PT: 10008612) | 13 | 3.83 (2.21-6.64) | 3.83 (26.69) | 1.92 (0.87) | 3.78 (2.18) |  |
| Cholangitis acute (PT: 10008605) | 5 | 13.67 (5.52-33.87) | 13.67 (54.79) | 3.68 (0.88) | 12.82 (5.17) |  |
| Acute cholecystitis necrotic (PT: 10090325) | 3 | 20.50 (6.23-67.45) | 20.50 (50.26) | 4.22 (0.25) | 18.61 (5.66) |  |
| **Investigations (SOC: 10022891)** | | | | | | |
| Lipase increased (PT: 10024574) | 23 | 11.59 (7.61-17.66) | 11.58 (209.68) | 3.46 (2.35) | 10.98 (7.20) |  |
| Amylase increased (PT: 10002016) | 7 | 5.17 (2.44-10.96) | 5.17 (22.93) | 2.34 (0.71) | 5.06 (2.39) |  |
| Pancreatic enzymes increased (PT: 10061900) | 6 | 15.52 (6.75-35.66) | 15.51 (75.36) | 3.85 (1.16) | 14.43 (6.28) |  |
| Gastric pH decreased (PT: 10060041) | 5 | 31.90 (12.37-82.22) | 31.89 (128.23) | 4.78 (1.06) | 27.48 (10.66) |  |
| Blood electrolytes decreased (PT: 10061715) | 3 | 7.46 (2.35-23.63) | 7.45 (16.14) | 2.85 (0.01) | 7.21 (2.28) |  |
| **Psychiatric disorders (SOC: 10037175)** | | | | | | |
| Sleep disorder due to general medical condition, insomnia type (PT: 10040986) | 19 | 33.08 (20.32-53.85) | 33.05 (503.55) | 4.82 (2.88) | 28.33 (17.40) |  |
| **Nervous system disorders (SOC: 10029205)** | | | | | | |
| Allodynia (PT: 10053552) | 8 | 23.56 (11.30-49.11) | 23.55 (153.81) | 4.40 (1.68) | 21.08 (10.11) |  |
| Ophthalmic migraine (PT: 10030867) | 4 | 16.64 (5.99-46.23) | 16.64 (54.09) | 3.94 (0.63) | 15.39 (5.54) |  |
| **Endocrine disorders (SOC: 10014698)** | | | | | | |
| Thyroid mass (PT: 10058900) | 5 | 7.30 (2.99-17.85) | 7.30 (26.20) | 2.82 (0.61) | 7.07 (2.89) |  |
| Goitre (PT: 10018498) | 4 | 7.65 (2.82-20.80) | 7.65 (22.25) | 2.89 (0.37) | 7.40 (2.72) |  |
| **Infections and infestations (SOC: 10021881)** | | | | | | |
| Injection site pustule (PT: 10054994) | 3 | 26.10 (7.81-87.19) | 26.09 (63.70) | 4.53 (0.27) | 23.08 (6.91) |  |

For both tirzepatide and all other drugs, only reports of males were included. **Abbreviations:** PT, preferred term; ROR, reporting odds ratio; CI, confidence interval; PRR, proportional reporting ratio; χ2, chi-squared; IC, information component; IC025, lower limit of 95% confidence interval of IC; EBGM, empirical Bayesian geometric mean; EBGM05, lower limit of 95% confidence interval of EBGM.

## Supplementary Table S13. Number and signal strength of tirzepatide-related signals at the PT level from the FAERS database stratified by females.

| **PT** | **Number** | **ROR (95% CI)** | **PRR (χ2)** | **IC (IC025)** | **EBGM (EBGM05)** |
| --- | --- | --- | --- | --- | --- |
| **General disorders and administration site conditions (SOC: 10018065)** | | | | | |
| Injection site pain (PT: 10022086) | 4981 | 15.91 (15.43-16.41) | 14.81 (55420.07) | 3.69 (3.64) | 12.86 (12.47) |
| Injection site haemorrhage (PT: 10022067) | 2090 | 26.08 (24.83-27.40) | 25.31 (38172.23) | 4.32 (4.24) | 19.99 (19.03) |
| Injection site erythema (PT: 10022061) | 1698 | 14.80 (14.05-15.58) | 14.45 (18360.16) | 3.65 (3.57) | 12.59 (11.96) |
| Injection site bruising (PT: 10022052) | 1163 | 12.69 (11.93-13.50) | 12.49 (10817.12) | 3.47 (3.37) | 11.09 (10.43) |
| Injection site pruritus (PT: 10022093) | 901 | 12.45 (11.61-13.36) | 12.30 (8243.41) | 3.45 (3.33) | 10.95 (10.21) |
| Injection site mass (PT: 10022081) | 529 | 14.21 (12.96-15.57) | 14.10 (5573.20) | 3.62 (3.46) | 12.33 (11.25) |
| Injection site rash (PT: 10022094) | 496 | 12.77 (11.62-14.03) | 12.68 (4683.61) | 3.49 (3.32) | 11.24 (10.23) |
| Injection site injury (PT: 10066083) | 467 | 84.09 (74.13-95.39) | 83.52 (19784.79) | 5.46 (5.16) | 43.87 (38.68) |
| Injection site swelling (PT: 10053425) | 463 | 5.16 (4.70-5.67) | 5.13 (1458.80) | 2.30 (2.14) | 4.91 (4.47) |
| Injection site urticaria (PT: 10022107) | 403 | 12.08 (10.89-13.41) | 12.02 (3594.11) | 3.42 (3.24) | 10.72 (9.66) |
| Injury associated with device (PT: 10069803) | 385 | 9.26 (8.34-10.29) | 9.21 (2559.70) | 3.08 (2.90) | 8.45 (7.61) |
| Injection site reaction (PT: 10022095) | 252 | 4.18 (3.69-4.75) | 4.17 (581.49) | 2.01 (1.81) | 4.03 (3.55) |
| Injection site irritation (PT: 10022079) | 144 | 14.18 (11.89-16.91) | 14.15 (1521.85) | 3.63 (3.26) | 12.37 (10.38) |
| Injection site warmth (PT: 10022112) | 111 | 6.84 (5.64-8.30) | 6.83 (513.58) | 2.68 (2.33) | 6.42 (5.29) |
| Injection site discomfort (PT: 10054266) | 104 | 8.26 (6.75-10.10) | 8.25 (606.96) | 2.93 (2.55) | 7.64 (6.25) |
| Injection site coldness (PT: 10050082) | 89 | 157.82 (111.85-222.68) | 157.61 (5045.35) | 5.86 (4.76) | 58.05 (41.14) |
| Injection site hypersensitivity (PT: 10022071) | 73 | 17.65 (13.73-22.68) | 17.63 (958.08) | 3.90 (3.28) | 14.91 (11.60) |
| Injection site vesicles (PT: 10022111) | 62 | 7.57 (5.84-9.81) | 7.57 (326.07) | 2.82 (2.31) | 7.06 (5.45) |
| Injection site paraesthesia (PT: 10022088) | 56 | 28.12 (20.83-37.96) | 28.10 (1116.25) | 4.44 (3.56) | 21.67 (16.05) |
| Injection site discolouration (PT: 10051572) | 53 | 3.48 (2.64-4.57) | 3.47 (89.92) | 1.76 (1.29) | 3.38 (2.57) |
| Injection site inflammation (PT: 10022078) | 45 | 7.20 (5.31-9.75) | 7.19 (222.27) | 2.75 (2.14) | 6.74 (4.97) |
| Injection site induration (PT: 10022075) | 43 | 4.49 (3.30-6.09) | 4.48 (110.93) | 2.11 (1.56) | 4.32 (3.18) |
| Injection site indentation (PT: 10079277) | 24 | 5.78 (3.83-8.74) | 5.78 (89.17) | 2.46 (1.62) | 5.49 (3.64) |
| Injection site scar (PT: 10059009) | 21 | 6.08 (3.91-9.46) | 6.08 (83.50) | 2.53 (1.60) | 5.76 (3.70) |
| Injection site hypoaesthesia (PT: 10074586) | 21 | 9.88 (6.30-15.50) | 9.88 (151.05) | 3.17 (2.07) | 9.00 (5.74) |
| Injection site scab (PT: 10066210) | 14 | 17.56 (9.91-31.14) | 17.56 (183.06) | 3.89 (2.13) | 14.87 (8.39) |
| Injection site laceration (PT: 10067253) | 10 | 23.16 (11.56-46.40) | 23.16 (168.74) | 4.22 (1.87) | 18.64 (9.30) |
| Thirst decreased (PT: 10050200) | 9 | 12.51 (6.23-25.11) | 12.51 (83.68) | 3.47 (1.49) | 11.11 (5.53) |
| Injection site thrombosis (PT: 10022104) | 4 | 14.45 (5.03-41.52) | 14.45 (43.17) | 3.65 (0.52) | 12.60 (4.38) |
| Injection site dermatitis (PT: 10022056) | 3 | 22.58 (6.37-80.02) | 22.58 (49.50) | 4.19 (0.15) | 18.26 (5.15) |
| **Gastrointestinal disorders (SOC: 10017947)** | | | | | |
| Nausea (PT: 10028813) | 3402 | 3.92 (3.79-4.06) | 3.77 (6751.66) | 1.87 (1.82) | 3.66 (3.54) |
| Vomiting (PT: 10047700) | 1222 | 2.43 (2.29-2.57) | 2.40 (979.05) | 1.24 (1.15) | 2.36 (2.23) |
| Constipation (PT: 10010774) | 889 | 3.73 (3.48-3.99) | 3.69 (1680.66) | 1.84 (1.74) | 3.58 (3.35) |
| Eructation (PT: 10015137) | 461 | 29.39 (26.45-32.65) | 29.20 (9489.66) | 4.48 (4.26) | 22.31 (20.08) |
| Flatulence (PT: 10016766) | 230 | 4.33 (3.79-4.94) | 4.32 (560.04) | 2.06 (1.84) | 4.17 (3.65) |
| Pancreatitis (PT: 10033645) | 199 | 5.85 (5.07-6.75) | 5.84 (749.33) | 2.47 (2.23) | 5.54 (4.80) |
| Impaired gastric emptying (PT: 10021518) | 130 | 10.37 (8.65-12.44) | 10.35 (985.74) | 3.23 (2.88) | 9.39 (7.83) |
| Gastrointestinal sounds abnormal (PT: 10067715) | 28 | 4.80 (3.28-7.02) | 4.80 (79.95) | 2.20 (1.48) | 4.61 (3.15) |
| Vomiting projectile (PT: 10047708) | 26 | 8.36 (5.59-12.49) | 8.36 (154.13) | 2.95 (2.05) | 7.73 (5.17) |
| Oesophageal rupture (PT: 10052211) | 4 | 6.34 (2.30-17.47) | 6.34 (16.80) | 2.58 (0.23) | 5.99 (2.17) |
| **Metabolism and nutrition disorders (SOC: 10027433)** | | | | | |
| Dehydration (PT: 10012174) | 276 | 2.39 (2.12-2.69) | 2.38 (216.12) | 1.23 (1.05) | 2.35 (2.08) |
| Feeding disorder (PT: 10061148) | 161 | 5.39 (4.60-6.32) | 5.38 (542.57) | 2.36 (2.09) | 5.14 (4.38) |
| Food craving (PT: 10056465) | 98 | 17.01 (13.71-21.11) | 16.99 (1241.27) | 3.85 (3.36) | 14.46 (11.65) |
| Fluid intake reduced (PT: 10056291) | 34 | 6.69 (4.72-9.48) | 6.69 (153.21) | 2.65 (1.94) | 6.30 (4.44) |
| Food aversion (PT: 10049238) | 16 | 6.63 (3.99-11.02) | 6.63 (71.25) | 2.64 (1.53) | 6.24 (3.76) |
| Starvation ketoacidosis (PT: 10082528) | 5 | 22.58 (8.47-60.17) | 22.58 (82.49) | 4.19 (0.92) | 18.26 (6.85) |
| **Hepatobiliary disorders (SOC: 10019805)** | | | | | |
| Gallbladder disorder (PT: 10017626) | 47 | 3.56 (2.66-4.76) | 3.56 (83.06) | 1.79 (1.29) | 3.46 (2.58) |
| Cholecystitis (PT: 10008612) | 28 | 3.71 (2.55-5.42) | 3.71 (53.33) | 1.85 (1.18) | 3.61 (2.47) |
| **Reproductive system and breast disorders (SOC: 10038604)** | | | | | |
| Menstrual disorder (PT: 10027327) | 33 | 3.13 (2.21-4.43) | 3.13 (46.16) | 1.61 (1.02) | 3.06 (2.16) |
| Postmenopausal haemorrhage (PT: 10055870) | 29 | 6.95 (4.76-10.14) | 6.95 (137.11) | 2.71 (1.91) | 6.52 (4.47) |
| Oligomenorrhoea (PT: 10030295) | 10 | 5.13 (2.71-9.71) | 5.13 (31.48) | 2.30 (0.96) | 4.91 (2.60) |
| **Investigations (SOC: 10022891)** | | | | | |
| Lipase increased (PT: 10024574) | 36 | 7.71 (5.48-10.83) | 7.70 (193.54) | 2.84 (2.12) | 7.18 (5.11) |
| Gastric pH decreased (PT: 10060041) | 8 | 6.12 (2.99-12.53) | 6.12 (32.11) | 2.54 (0.92) | 5.80 (2.83) |
| Pancreatic enzymes increased (PT: 10061900) | 7 | 5.65 (2.63-12.11) | 5.64 (25.18) | 2.43 (0.74) | 5.37 (2.50) |
| Blood folate decreased (PT: 10005527) | 5 | 5.72 (2.32-14.11) | 5.72 (18.30) | 2.44 (0.42) | 5.44 (2.20) |
| Blood calcitonin increased (PT: 10005390) | 3 | 24.63 (6.87-88.30) | 24.63 (53.44) | 4.29 (0.16) | 19.57 (5.46) |
| **Skin and subcutaneous tissue disorders (SOC: 10040785)** | | | | | |
| Sensitive skin (PT: 10081765) | 43 | 3.95 (2.91-5.36) | 3.95 (90.81) | 1.94 (1.40) | 3.83 (2.82) |
| Skin laxity (PT: 10057064) | 6 | 6.30 (2.75-14.42) | 6.30 (25.01) | 2.57 (0.66) | 5.96 (2.60) |
| Skin sensitisation (PT: 10070835) | 6 | 5.65 (2.47-12.88) | 5.64 (21.58) | 2.43 (0.59) | 5.37 (2.35) |
| **Psychiatric disorders (SOC: 10037175)** | | | | | |
| Sleep disorder due to general medical condition, insomnia type (PT: 10040986) | 48 | 2.81 (2.11-3.74) | 2.81 (54.12) | 1.46 (0.99) | 2.75 (2.06) |
| **Nervous system disorders (SOC: 10029205)** | | | | | |
| Allodynia (PT: 10053552) | 16 | 8.31 (4.98-13.86) | 8.30 (94.15) | 2.94 (1.73) | 7.69 (4.61) |
| Hypoglycaemic unconsciousness (PT: 10065981) | 9 | 11.78 (5.88-23.60) | 11.78 (78.54) | 3.40 (1.45) | 10.54 (5.26) |
| Wernicke's encephalopathy (PT: 10047911) | 5 | 10.04 (3.98-25.28) | 10.03 (36.61) | 3.19 (0.70) | 9.13 (3.62) |
| **Infections and infestations (SOC: 10021881)** | | | | | |
| Injection site infection (PT: 10022076) | 16 | 6.18 (3.72-10.25) | 6.18 (64.96) | 2.55 (1.46) | 5.84 (3.52) |
| Injection site cellulitis (PT: 10050057) | 7 | 5.45 (2.54-11.69) | 5.45 (23.99) | 2.38 (0.71) | 5.20 (2.42) |
| **Blood and lymphatic system disorders (SOC: 10005329)** | | | | | |
| Lymph node pain (PT: 10025182) | 11 | 4.75 (2.59-8.72) | 4.75 (30.97) | 2.19 (0.96) | 4.57 (2.49) |

For both tirzepatide and all other drugs, only reports of females were included. **Abbreviations:** PT, preferred term; ROR, reporting odds ratio; CI, confidence interval; PRR, proportional reporting ratio; χ2, chi-squared; IC, information component; IC025, lower limit of 95% confidence interval of IC; EBGM, empirical Bayesian geometric mean; EBGM05, lower limit of 95% confidence interval of EBGM.

## Supplementary Table S14. Number and signal strength of tirzepatide-related signals at the PT level from the FAERS database stratified by patients 18 to 65 years.

| **PT** | **Number** | **ROR (95% CI)** | **PRR (χ2)** | **IC (IC025)** | **EBGM (EBGM05)** |
| --- | --- | --- | --- | --- | --- |
| **General disorders and administration site conditions (SOC: 10018065)** | | | | | |
| Injection site pain (PT: 10022086) | 4670 | 20.63 (19.95-21.33) | 19.04 (63629.89) | 3.94 (3.88) | 15.30 (14.80) |
| Injection site haemorrhage (PT: 10022067) | 2063 | 34.25 (32.49-36.10) | 33.06 (44189.03) | 4.53 (4.44) | 23.05 (21.87) |
| Injection site erythema (PT: 10022061) | 1402 | 16.18 (15.26-17.15) | 15.81 (16012.95) | 3.72 (3.62) | 13.17 (12.42) |
| Injection site bruising (PT: 10022052) | 1004 | 16.47 (15.38-17.65) | 16.20 (11732.09) | 3.75 (3.63) | 13.44 (12.54) |
| Injection site pruritus (PT: 10022093) | 618 | 11.25 (10.34-12.25) | 11.14 (4955.05) | 3.29 (3.15) | 9.80 (9.00) |
| Injection site mass (PT: 10022081) | 517 | 16.18 (14.70-17.80) | 16.04 (5979.94) | 3.74 (3.56) | 13.33 (12.11) |
| Injection site injury (PT: 10066083) | 501 | 122.72 (106.34-141.63) | 121.67 (22460.08) | 5.53 (5.24) | 46.19 (40.03) |
| Injection site swelling (PT: 10053425) | 381 | 5.00 (4.51-5.55) | 4.98 (1134.41) | 2.24 (2.07) | 4.72 (4.25) |
| Injection site rash (PT: 10022094) | 345 | 11.47 (10.23-12.85) | 11.40 (2833.16) | 3.32 (3.12) | 10.00 (8.92) |
| Injection site urticaria (PT: 10022107) | 331 | 13.08 (11.64-14.71) | 13.01 (3116.27) | 3.48 (3.27) | 11.19 (9.95) |
| Injury associated with device (PT: 10069803) | 262 | 23.79 (20.69-27.35) | 23.68 (4296.57) | 4.18 (3.89) | 18.12 (15.76) |
| Injection site reaction (PT: 10022095) | 150 | 3.18 (2.70-3.74) | 3.17 (213.75) | 1.62 (1.36) | 3.08 (2.61) |
| Injection site discomfort (PT: 10054266) | 123 | 12.15 (10.04-14.71) | 12.13 (1076.69) | 3.40 (3.01) | 10.54 (8.71) |
| Injection site irritation (PT: 10022079) | 117 | 14.65 (12.01-17.87) | 14.62 (1236.70) | 3.63 (3.20) | 12.34 (10.12) |
| Injection site coldness (PT: 10050082) | 97 | 186.28 (128.01-271.09) | 185.97 (5023.38) | 5.73 (4.73) | 53.07 (36.46) |
| Injection site warmth (PT: 10022112) | 84 | 6.13 (4.91-7.66) | 6.13 (332.43) | 2.52 (2.11) | 5.73 (4.58) |
| Injection site vesicles (PT: 10022111) | 66 | 9.13 (7.07-11.80) | 9.12 (424.35) | 3.04 (2.52) | 8.22 (6.36) |
| Injection site paraesthesia (PT: 10022088) | 55 | 33.70 (24.48-46.40) | 33.67 (1192.51) | 4.55 (3.61) | 23.34 (16.96) |
| Injection site hypersensitivity (PT: 10022071) | 52 | 15.73 (11.66-21.23) | 15.72 (589.57) | 3.71 (2.98) | 13.11 (9.71) |
| Injection site discolouration (PT: 10051572) | 41 | 3.97 (2.90-5.44) | 3.97 (86.29) | 1.93 (1.38) | 3.81 (2.78) |
| Injection site inflammation (PT: 10022078) | 34 | 6.39 (4.50-9.07) | 6.38 (141.96) | 2.57 (1.87) | 5.95 (4.19) |
| Injection site induration (PT: 10022075) | 29 | 3.43 (2.36-4.98) | 3.43 (47.68) | 1.73 (1.08) | 3.32 (2.29) |
| Injection site indentation (PT: 10079277) | 24 | 9.25 (6.05-14.15) | 9.25 (156.73) | 3.06 (2.07) | 8.32 (5.44) |
| Injection site scar (PT: 10059009) | 21 | 7.19 (4.59-11.25) | 7.18 (101.74) | 2.73 (1.75) | 6.63 (4.23) |
| Injection site laceration (PT: 10067253) | 12 | 36.43 (18.22-72.86) | 36.43 (275.64) | 4.62 (2.18) | 24.62 (12.31) |
| Injection site scab (PT: 10066210) | 10 | 11.39 (5.85-22.17) | 11.38 (81.92) | 3.32 (1.52) | 9.98 (5.12) |
| Injection site hypoaesthesia (PT: 10074586) | 10 | 5.65 (2.97-10.75) | 5.65 (35.50) | 2.41 (1.02) | 5.31 (2.79) |
| Injection site thrombosis (PT: 10022104) | 5 | 19.17 (7.16-51.35) | 19.17 (68.18) | 3.94 (0.85) | 15.39 (5.74) |
| Thirst decreased (PT: 10050200) | 4 | 7.47 (2.67-20.91) | 7.47 (20.34) | 2.78 (0.29) | 6.87 (2.45) |
| **Gastrointestinal disorders (SOC: 10017947)** | | | | | |
| Nausea (PT: 10028813) | 2562 | 3.66 (3.51-3.81) | 3.54 (4505.24) | 1.77 (1.71) | 3.42 (3.28) |
| Vomiting (PT: 10047700) | 969 | 2.35 (2.20-2.50) | 2.32 (713.20) | 1.19 (1.09) | 2.28 (2.14) |
| Constipation (PT: 10010774) | 639 | 3.98 (3.67-4.31) | 3.94 (1336.15) | 1.92 (1.80) | 3.79 (3.50) |
| Eructation (PT: 10015137) | 390 | 35.35 (31.32-39.90) | 35.12 (8725.65) | 4.59 (4.33) | 24.02 (21.28) |
| Flatulence (PT: 10016766) | 209 | 5.26 (4.57-6.05) | 5.24 (670.01) | 2.31 (2.08) | 4.96 (4.31) |
| Impaired gastric emptying (PT: 10021518) | 164 | 13.08 (11.07-15.45) | 13.04 (1547.21) | 3.49 (3.16) | 11.21 (9.50) |
| Pancreatitis (PT: 10033645) | 132 | 3.86 (3.24-4.60) | 3.85 (264.82) | 1.89 (1.60) | 3.71 (3.11) |
| Gastrointestinal sounds abnormal (PT: 10067715) | 22 | 4.57 (2.97-7.03) | 4.57 (57.67) | 2.12 (1.30) | 4.36 (2.83) |
| Vomiting projectile (PT: 10047708) | 20 | 8.38 (5.27-13.31) | 8.37 (116.49) | 2.93 (1.87) | 7.61 (4.79) |
| Oesophageal rupture (PT: 10052211) | 5 | 9.85 (3.87-25.05) | 9.85 (35.00) | 3.14 (0.67) | 8.79 (3.46) |
| **Metabolism and nutrition disorders (SOC: 10027433)** | | | | | |
| Dehydration (PT: 10012174) | 213 | 2.66 (2.32-3.05) | 2.66 (212.78) | 1.38 (1.17) | 2.60 (2.27) |
| Feeding disorder (PT: 10061148) | 121 | 6.32 (5.25-7.61) | 6.31 (497.79) | 2.56 (2.23) | 5.89 (4.89) |
| Food craving (PT: 10056465) | 78 | 18.24 (14.23-23.38) | 18.21 (1015.25) | 3.88 (3.29) | 14.77 (11.52) |
| Fluid intake reduced (PT: 10056291) | 19 | 6.07 (3.80-9.70) | 6.07 (74.30) | 2.51 (1.53) | 5.68 (3.56) |
| Food aversion (PT: 10049238) | 14 | 8.95 (5.14-15.59) | 8.95 (88.02) | 3.01 (1.66) | 8.08 (4.64) |
| Starvation ketoacidosis (PT: 10082528) | 5 | 22.77 (8.34-62.15) | 22.77 (79.28) | 4.14 (0.88) | 17.58 (6.44) |
| **Skin and subcutaneous tissue disorders (SOC: 10040785)** | | | | | |
| Sensitive skin (PT: 10081765) | 35 | 3.72 (2.65-5.22) | 3.72 (66.15) | 1.84 (1.25) | 3.59 (2.55) |
| Skin sensitisation (PT: 10070835) | 6 | 5.20 (2.27-11.91) | 5.20 (19.02) | 2.30 (0.52) | 4.92 (2.15) |
| Skin laxity (PT: 10057064) | 4 | 7.29 (2.61-20.36) | 7.29 (19.72) | 2.75 (0.28) | 6.71 (2.40) |
| **Investigations (SOC: 10022891)** | | | | | |
| Lipase increased (PT: 10024574) | 19 | 4.01 (2.53-6.37) | 4.01 (40.73) | 1.95 (1.09) | 3.86 (2.43) |
| Gastric pH decreased (PT: 10060041) | 9 | 9.11 (4.55-18.21) | 9.11 (57.73) | 3.04 (1.28) | 8.21 (4.10) |
| Blood calcitonin increased (PT: 10005390) | 3 | 18.21 (5.14-64.55) | 18.21 (39.04) | 3.88 (0.10) | 14.77 (4.17) |
| **Nervous system disorders (SOC: 10029205)** | | | | | |
| Allodynia (PT: 10053552) | 16 | 6.04 (3.63-10.06) | 6.04 (62.14) | 2.50 (1.42) | 5.65 (3.40) |
| Wernicke's encephalopathy (PT: 10047911) | 4 | 7.47 (2.67-20.91) | 7.47 (20.34) | 2.78 (0.29) | 6.87 (2.45) |
| **Reproductive system and breast disorders (SOC: 10038604)** | | | | | |
| Postmenopausal haemorrhage (PT: 10055870) | 17 | 5.84 (3.57-9.58) | 5.84 (63.16) | 2.45 (1.42) | 5.48 (3.34) |
| **Infections and infestations (SOC: 10021881)** | | | | | |
| Injection site infection (PT: 10022076) | 11 | 5.01 (2.72-9.23) | 5.01 (33.02) | 2.25 (0.99) | 4.75 (2.58) |
| Injection site pustule (PT: 10054994) | 3 | 9.94 (2.97-33.20) | 9.93 (21.21) | 3.15 (0.02) | 8.86 (2.65) |
| **Blood and lymphatic system disorders (SOC: 10005329)** | | | | | |
| Lymph node pain (PT: 10025182) | 9 | 4.43 (2.26-8.68) | 4.43 (22.53) | 2.08 (0.73) | 4.23 (2.16) |

For both tirzepatide and all other drugs, only reports of patients 18 to 65 years were included. **Abbreviations:** PT, preferred term; ROR, reporting odds ratio; CI, confidence interval; PRR, proportional reporting ratio; χ2, chi-squared; IC, information component; IC025, lower limit of 95% confidence interval of IC; EBGM, empirical Bayesian geometric mean; EBGM05, lower limit of 95% confidence interval of EBGM.

## Supplementary Table S15. Number and signal strength of tirzepatide-related signals at the PT level from the FAERS database stratified by patients over 65 years.

| **PT** | **Number** | **ROR (95% CI)** | **PRR (χ2)** | **IC (IC025)** | **EBGM (EBGM05)** |
| --- | --- | --- | --- | --- | --- |
| **General disorders and administration site conditions (SOC: 10018065)** | | | | | |
| Injection site pain (PT: 10022086) | 648 | 16.54 (15.24-17.96) | 15.70 (8297.86) | 3.87 (3.72) | 14.63 (13.47) |
| Injection site haemorrhage (PT: 10022067) | 281 | 21.93 (19.36-24.83) | 21.44 (4948.28) | 4.28 (4.01) | 19.45 (17.17) |
| Injection site erythema (PT: 10022061) | 245 | 30.51 (26.64-34.93) | 29.90 (5954.07) | 4.71 (4.37) | 26.12 (22.81) |
| Injection site bruising (PT: 10022052) | 143 | 17.29 (14.56-20.53) | 17.10 (1997.03) | 3.98 (3.59) | 15.82 (13.32) |
| Injection site pruritus (PT: 10022093) | 116 | 26.98 (22.20-32.77) | 26.72 (2533.03) | 4.57 (4.03) | 23.68 (19.49) |
| Injury associated with device (PT: 10069803) | 102 | 26.08 (21.20-32.08) | 25.87 (2158.43) | 4.52 (3.94) | 23.00 (18.70) |
| Injection site mass (PT: 10022081) | 100 | 15.13 (12.34-18.56) | 15.02 (1217.15) | 3.81 (3.34) | 14.03 (11.44) |
| Injection site swelling (PT: 10053425) | 65 | 9.73 (7.58-12.49) | 9.69 (483.15) | 3.21 (2.68) | 9.28 (7.23) |
| Injection site urticaria (PT: 10022107) | 48 | 32.81 (24.17-44.55) | 32.68 (1266.28) | 4.82 (3.74) | 28.21 (20.78) |
| Injection site rash (PT: 10022094) | 48 | 19.67 (14.62-26.47) | 19.60 (771.23) | 4.16 (3.30) | 17.93 (13.32) |
| Injection site injury (PT: 10066083) | 42 | 58.19 (41.24-82.10) | 57.99 (1821.23) | 5.50 (3.98) | 45.12 (31.98) |
| Injection site reaction (PT: 10022095) | 28 | 7.53 (5.16-10.98) | 7.51 (152.38) | 2.86 (2.03) | 7.28 (4.99) |
| Injection site discomfort (PT: 10054266) | 19 | 12.13 (7.63-19.27) | 12.11 (182.52) | 3.52 (2.24) | 11.47 (7.22) |
| Injection site warmth (PT: 10022112) | 19 | 19.50 (12.17-31.25) | 19.47 (303.27) | 4.16 (2.60) | 17.82 (11.12) |
| Injection site hypersensitivity (PT: 10022071) | 12 | 49.75 (26.42-93.68) | 49.70 (458.17) | 5.32 (2.43) | 39.96 (21.22) |
| Injection site irritation (PT: 10022079) | 12 | 22.74 (12.51-41.34) | 22.72 (223.65) | 4.36 (2.19) | 20.49 (11.28) |
| Injection site paraesthesia (PT: 10022088) | 10 | 52.36 (26.09-105.11) | 52.32 (398.53) | 5.38 (2.18) | 41.63 (20.74) |
| Injection site discolouration (PT: 10051572) | 10 | 8.02 (4.26-15.10) | 8.02 (59.04) | 2.95 (1.37) | 7.74 (4.11) |
| Injection site coldness (PT: 10050082) | 9 | 179.07 (72.75-440.77) | 178.94 (838.15) | 6.56 (2.07) | 94.65 (38.45) |
| Injection site vesicles (PT: 10022111) | 9 | 14.21 (7.22-27.95) | 14.20 (103.09) | 3.74 (1.63) | 13.32 (6.77) |
| Injection site induration (PT: 10022075) | 8 | 8.74 (4.31-17.76) | 8.74 (52.53) | 3.07 (1.22) | 8.41 (4.14) |
| Injection site inflammation (PT: 10022078) | 7 | 14.36 (6.66-30.92) | 14.35 (81.07) | 3.75 (1.33) | 13.45 (6.24) |
| Injection site hypoaesthesia (PT: 10074586) | 6 | 51.89 (21.13-127.46) | 51.87 (237.39) | 5.37 (1.39) | 41.34 (16.83) |
| Injection site indentation (PT: 10079277) | 4 | 5.85 (2.16-15.81) | 5.85 (15.62) | 2.51 (0.24) | 5.71 (2.11) |
| Injection site scar (PT: 10059009) | 3 | 11.26 (3.52-36.03) | 11.25 (26.53) | 3.42 (0.15) | 10.70 (3.34) |
| **Gastrointestinal disorders (SOC: 10017947)** | | | | | |
| Nausea (PT: 10028813) | 514 | 4.19 (3.83-4.58) | 4.05 (1169.97) | 2.00 (1.86) | 3.99 (3.65) |
| Constipation (PT: 10010774) | 239 | 4.08 (3.58-4.64) | 4.02 (533.53) | 1.98 (1.78) | 3.96 (3.48) |
| Vomiting (PT: 10047700) | 208 | 2.89 (2.52-3.32) | 2.86 (248.98) | 1.50 (1.29) | 2.83 (2.47) |
| Abdominal discomfort (PT: 10000059) | 109 | 3.56 (2.94-4.31) | 3.54 (195.50) | 1.80 (1.49) | 3.49 (2.89) |
| Eructation (PT: 10015137) | 100 | 32.59 (26.36-40.29) | 32.33 (2612.08) | 4.80 (4.15) | 27.95 (22.61) |
| Flatulence (PT: 10016766) | 75 | 6.83 (5.42-8.60) | 6.79 (358.64) | 2.72 (2.28) | 6.60 (5.24) |
| Pancreatitis (PT: 10033645) | 25 | 4.22 (2.84-6.28) | 4.22 (60.08) | 2.05 (1.31) | 4.15 (2.79) |
| Impaired gastric emptying (PT: 10021518) | 19 | 14.55 (9.13-23.19) | 14.53 (223.08) | 3.77 (2.39) | 13.61 (8.54) |
| Retching (PT: 10038776) | 13 | 3.56 (2.06-6.17) | 3.56 (23.52) | 1.81 (0.79) | 3.52 (2.03) |
| Gastrointestinal sounds abnormal (PT: 10067715) | 11 | 11.46 (6.24-21.05) | 11.45 (99.21) | 3.44 (1.72) | 10.88 (5.92) |
| Regurgitation (PT: 10067171) | 6 | 11.15 (4.90-25.39) | 11.15 (52.49) | 3.41 (1.03) | 10.61 (4.66) |
| Food poisoning (PT: 10016952) | 6 | 6.00 (2.66-13.51) | 5.99 (24.24) | 2.55 (0.68) | 5.85 (2.60) |
| Vomiting projectile (PT: 10047708) | 4 | 9.47 (3.47-25.83) | 9.47 (28.92) | 3.18 (0.46) | 9.08 (3.33) |
| **Metabolism and nutrition disorders (SOC: 10027433)** | | | | | |
| Feeding disorder (PT: 10061148) | 33 | 5.55 (3.92-7.85) | 5.54 (119.41) | 2.44 (1.76) | 5.41 (3.83) |
| Food craving (PT: 10056465) | 12 | 35.12 (19.01-64.89) | 35.09 (337.77) | 4.91 (2.35) | 29.97 (16.22) |
| Fluid intake reduced (PT: 10056291) | 10 | 7.74 (4.11-14.57) | 7.74 (56.46) | 2.90 (1.34) | 7.48 (3.98) |
| Appetite disorder (PT: 10060961) | 8 | 4.67 (2.31-9.41) | 4.66 (22.51) | 2.20 (0.74) | 4.58 (2.27) |
| Hyperphagia (PT: 10020710) | 4 | 8.37 (3.08-22.78) | 8.37 (24.91) | 3.01 (0.41) | 8.07 (2.97) |
| Food aversion (PT: 10049238) | 4 | 8.74 (3.21-23.80) | 8.74 (26.26) | 3.07 (0.43) | 8.41 (3.09) |
| **Psychiatric disorders (SOC: 10037175)** | | | | | |
| Sleep disorder due to general medical condition, insomnia type (PT: 10040986) | 19 | 38.22 (23.38-62.46) | 38.16 (576.81) | 5.01 (2.95) | 32.17 (19.69) |
| **Investigations (SOC: 10022891)** | | | | | |
| Lipase increased (PT: 10024574) | 7 | 5.88 (2.77-12.46) | 5.87 (27.49) | 2.52 (0.81) | 5.73 (2.70) |
| Pancreatic enzymes increased (PT: 10061900) | 3 | 12.69 (3.95-40.79) | 12.69 (30.37) | 3.58 (0.17) | 11.99 (3.73) |
| **Skin and subcutaneous tissue disorders (SOC: 10040785)** | | | | | |
| Sensitive skin (PT: 10081765) | 7 | 5.55 (2.62-11.76) | 5.54 (25.37) | 2.44 (0.76) | 5.42 (2.56) |
| **Endocrine disorders (SOC: 10014698)** | | | | | |
| Thyroid mass (PT: 10058900) | 6 | 8.52 (3.76-19.30) | 8.52 (38.19) | 3.04 (0.90) | 8.21 (3.63) |
| **Hepatobiliary disorders (SOC: 10019805)** | | | | | |
| Cholangitis acute (PT: 10008605) | 5 | 14.62 (5.90-36.27) | 14.62 (59.09) | 3.77 (0.91) | 13.69 (5.52) |
| **Infections and infestations (SOC: 10021881)** | | | | | |
| Injection site infection (PT: 10022076) | 4 | 17.68 (6.36-49.16) | 17.67 (57.78) | 4.03 (0.65) | 16.31 (5.87) |
| **Musculoskeletal and connective tissue disorders (SOC: 10028395)** | | | | | |
| Neck mass (PT: 10049146) | 4 | 7.10 (2.62-19.26) | 7.10 (20.24) | 2.78 (0.34) | 6.89 (2.54) |
| **Nervous system disorders (SOC: 10029205)** | | | | | |
| Allodynia (PT: 10053552) | 4 | 46.80 (15.74-139.09) | 46.78 (145.07) | 5.25 (0.74) | 38.06 (12.80) |

For both tirzepatide and all other drugs, only reports of patients over 65 years were included. **Abbreviations:** PT, preferred term; ROR, reporting odds ratio; CI, confidence interval; PRR, proportional reporting ratio; χ2, chi-squared; IC, information component; IC025, lower limit of 95% confidence interval of IC; EBGM, empirical Bayesian geometric mean; EBGM05, lower limit of 95% confidence interval of EBGM.

## Supplementary Table S16. Number and signal strength of tirzepatide-related signals at the PT level from the FAERS database based on reports taking 2.5 mg dose.

| **PT** | **Number** | **ROR (95% CI)** | **PRR (χ2)** | **IC (IC025)** | **EBGM (EBGM05)** |
| --- | --- | --- | --- | --- | --- |
| **General disorders and administration site conditions (SOC: 10018065)** | | | | | |
| Injection site pain (PT: 10022086) | 2452 | 15.26 (14.64-15.92) | 14.31 (29161.76) | 3.78 (3.71) | 13.72 (13.16) |
| Injection site haemorrhage (PT: 10022067) | 1055 | 23.73 (22.27-25.28) | 23.07 (20774.04) | 4.43 (4.31) | 21.56 (20.23) |
| Injection site erythema (PT: 10022061) | 752 | 14.58 (13.55-15.70) | 14.30 (8911.88) | 3.78 (3.65) | 13.72 (12.75) |
| Injection site bruising (PT: 10022052) | 579 | 14.98 (13.78-16.30) | 14.76 (7101.63) | 3.82 (3.67) | 14.14 (13.00) |
| Injection site pruritus (PT: 10022093) | 344 | 11.15 (10.00-12.42) | 11.05 (3039.75) | 3.42 (3.22) | 10.71 (9.61) |
| Injury associated with device (PT: 10069803) | 223 | 13.50 (11.80-15.44) | 13.43 (2460.06) | 3.69 (3.42) | 12.91 (11.29) |
| Injection site mass (PT: 10022081) | 220 | 9.78 (8.55-11.19) | 9.73 (1672.23) | 3.24 (2.99) | 9.47 (8.27) |
| Injection site swelling (PT: 10053425) | 214 | 5.19 (4.53-5.94) | 5.17 (707.89) | 2.35 (2.12) | 5.10 (4.45) |
| Injection site rash (PT: 10022094) | 208 | 12.01 (10.46-13.80) | 11.95 (2011.42) | 3.53 (3.25) | 11.55 (10.05) |
| Injection site injury (PT: 10066083) | 207 | 52.33 (45.15-60.64) | 52.04 (8883.28) | 5.48 (4.99) | 44.75 (38.61) |
| Injection site urticaria (PT: 10022107) | 146 | 9.99 (8.47-11.79) | 9.96 (1140.73) | 3.28 (2.95) | 9.68 (8.21) |
| Injection site reaction (PT: 10022095) | 103 | 3.57 (2.94-4.34) | 3.57 (188.30) | 1.82 (1.50) | 3.54 (2.91) |
| Injection site discomfort (PT: 10054266) | 70 | 10.61 (8.36-13.46) | 10.59 (587.89) | 3.36 (2.83) | 10.27 (8.09) |
| Injection site warmth (PT: 10022112) | 60 | 9.18 (7.10-11.86) | 9.16 (423.99) | 3.16 (2.61) | 8.93 (6.91) |
| Injection site irritation (PT: 10022079) | 53 | 11.08 (8.43-14.58) | 11.07 (468.95) | 3.42 (2.78) | 10.73 (8.15) |
| Injection site coldness (PT: 10050082) | 48 | 98.16 (70.96-135.77) | 98.03 (3509.04) | 6.23 (4.44) | 74.86 (54.12) |
| Injection site paraesthesia (PT: 10022088) | 41 | 41.51 (29.96-57.50) | 41.46 (1429.24) | 5.20 (3.84) | 36.72 (26.51) |
| Injection site discolouration (PT: 10051572) | 37 | 6.14 (4.43-8.50) | 6.13 (155.93) | 2.59 (1.94) | 6.03 (4.36) |
| Injection site hypersensitivity (PT: 10022071) | 32 | 16.09 (11.28-22.96) | 16.08 (430.29) | 3.94 (2.90) | 15.34 (10.75) |
| Injection site vesicles (PT: 10022111) | 28 | 6.90 (4.74-10.03) | 6.89 (138.07) | 2.76 (1.95) | 6.77 (4.65) |
| Injection site inflammation (PT: 10022078) | 24 | 8.59 (5.72-12.88) | 8.58 (156.44) | 3.07 (2.11) | 8.38 (5.58) |
| Injection site induration (PT: 10022075) | 20 | 4.28 (2.75-6.65) | 4.28 (49.55) | 2.08 (1.24) | 4.23 (2.72) |
| Injection site scar (PT: 10059009) | 15 | 9.09 (5.44-15.18) | 9.08 (104.86) | 3.15 (1.84) | 8.85 (5.30) |
| Injection site indentation (PT: 10079277) | 13 | 7.22 (4.16-12.51) | 7.21 (68.03) | 2.82 (1.52) | 7.07 (4.08) |
| Injection site hypoaesthesia (PT: 10074586) | 10 | 10.42 (5.55-19.56) | 10.42 (82.37) | 3.34 (1.58) | 10.11 (5.38) |
| Injection site scab (PT: 10066210) | 7 | 15.85 (7.42-33.88) | 15.85 (92.69) | 3.92 (1.40) | 15.13 (7.08) |
| Injection site laceration (PT: 10067253) | 7 | 29.97 (13.80-65.09) | 29.96 (178.82) | 4.78 (1.60) | 27.43 (12.63) |
| Early satiety (PT: 10059186) | 5 | 6.59 (2.72-15.99) | 6.59 (23.23) | 2.70 (0.56) | 6.48 (2.67) |
| Thirst decreased (PT: 10050200) | 4 | 11.36 (4.19-30.82) | 11.36 (36.48) | 3.46 (0.55) | 11.00 (4.06) |
| **Gastrointestinal disorders (SOC: 10017947)** | | | | | |
| Nausea (PT: 10028813) | 2185 | 5.58 (5.34-5.83) | 5.30 (7592.15) | 2.39 (2.32) | 5.23 (5.01) |
| Diarrhoea (PT: 10012735) | 1049 | 2.76 (2.59-2.93) | 2.71 (1131.79) | 1.43 (1.34) | 2.69 (2.53) |
| Vomiting (PT: 10047700) | 804 | 3.41 (3.18-3.66) | 3.36 (1323.87) | 1.74 (1.63) | 3.33 (3.10) |
| Constipation (PT: 10010774) | 675 | 5.39 (5.00-5.82) | 5.31 (2332.17) | 2.39 (2.27) | 5.24 (4.85) |
| Eructation (PT: 10015137) | 364 | 39.74 (35.62-44.34) | 39.36 (12087.97) | 5.13 (4.84) | 35.06 (31.43) |
| Abdominal discomfort (PT: 10000059) | 311 | 2.95 (2.63-3.30) | 2.93 (392.78) | 1.54 (1.37) | 2.91 (2.60) |
| Flatulence (PT: 10016766) | 204 | 7.12 (6.19-8.18) | 7.09 (1043.46) | 2.80 (2.55) | 6.95 (6.05) |
| Pancreatitis (PT: 10033645) | 103 | 4.81 (3.95-5.84) | 4.79 (304.87) | 2.24 (1.91) | 4.74 (3.90) |
| Impaired gastric emptying (PT: 10021518) | 41 | 5.85 (4.29-7.97) | 5.84 (161.65) | 2.52 (1.92) | 5.76 (4.23) |
| Gastrointestinal sounds abnormal (PT: 10067715) | 23 | 7.27 (4.81-10.99) | 7.27 (121.48) | 2.83 (1.91) | 7.12 (4.71) |
| Vomiting projectile (PT: 10047708) | 14 | 9.88 (5.80-16.82) | 9.87 (108.25) | 3.26 (1.85) | 9.60 (5.64) |
| Regurgitation (PT: 10067171) | 9 | 5.22 (2.70-10.08) | 5.22 (30.18) | 2.36 (0.94) | 5.15 (2.66) |
| Duodenogastric reflux (PT: 10060865) | 3 | 11.87 (3.75-37.59) | 11.87 (28.76) | 3.52 (0.18) | 11.47 (3.62) |
| **Metabolism and nutrition disorders (SOC: 10027433)** | | | | | |
| Dehydration (PT: 10012174) | 179 | 2.92 (2.52-3.39) | 2.92 (223.50) | 1.53 (1.30) | 2.90 (2.50) |
| Feeding disorder (PT: 10061148) | 100 | 6.74 (5.53-8.22) | 6.72 (477.21) | 2.72 (2.35) | 6.60 (5.42) |
| Food craving (PT: 10056465) | 46 | 16.58 (12.32-22.31) | 16.56 (638.71) | 3.98 (3.15) | 15.78 (11.73) |
| Fluid intake reduced (PT: 10056291) | 17 | 5.98 (3.70-9.66) | 5.98 (69.11) | 2.56 (1.52) | 5.88 (3.64) |
| Food aversion (PT: 10049238) | 12 | 10.33 (5.81-18.37) | 10.33 (97.88) | 3.33 (1.75) | 10.03 (5.64) |
| Lack of satiety (PT: 10064720) | 3 | 19.13 (5.96-61.38) | 19.13 (48.57) | 4.18 (0.28) | 18.08 (5.64) |
| **Psychiatric disorders (SOC: 10037175)** | | | | | |
| Sleep disorder due to general medical condition, insomnia type (PT: 10040986) | 41 | 6.31 (4.63-8.60) | 6.30 (179.41) | 2.63 (2.01) | 6.20 (4.55) |
| **Investigations (SOC: 10022891)** | | | | | |
| Lipase increased (PT: 10024574) | 19 | 5.92 (3.76-9.31) | 5.91 (76.13) | 2.54 (1.58) | 5.82 (3.70) |
| Gastric pH decreased (PT: 10060041) | 8 | 15.25 (7.50-31.00) | 15.24 (101.51) | 3.87 (1.55) | 14.58 (7.17) |
| Pancreatic enzymes increased (PT: 10061900) | 6 | 8.26 (3.67-18.58) | 8.26 (37.30) | 3.01 (0.90) | 8.07 (3.59) |
| Body mass index decreased (PT: 10005895) | 4 | 12.02 (4.43-32.63) | 12.02 (38.91) | 3.54 (0.57) | 11.61 (4.28) |
| **Reproductive system and breast disorders (SOC: 10038604)** | | | | | |
| Postmenopausal haemorrhage (PT: 10055870) | 12 | 8.85 (4.98-15.70) | 8.84 (81.18) | 3.11 (1.63) | 8.63 (4.86) |
| Oligomenorrhoea (PT: 10030295) | 6 | 9.52 (4.22-21.45) | 9.52 (44.38) | 3.21 (0.97) | 9.26 (4.11) |
| **Hepatobiliary disorders (SOC: 10019805)** | | | | | |
| Biliary colic (PT: 10004663) | 8 | 5.08 (2.53-10.22) | 5.08 (25.80) | 2.33 (0.82) | 5.02 (2.49) |
| Cholangitis acute (PT: 10008605) | 6 | 13.49 (5.96-30.55) | 13.49 (66.50) | 3.70 (1.14) | 12.97 (5.73) |
| **Infections and infestations (SOC: 10021881)** | | | | | |
| Injection site infection (PT: 10022076) | 8 | 5.64 (2.80-11.36) | 5.64 (30.02) | 2.48 (0.91) | 5.56 (2.76) |
| Injection site pustule (PT: 10054994) | 3 | 12.84 (4.05-40.75) | 12.84 (31.46) | 3.63 (0.20) | 12.37 (3.90) |
| **Nervous system disorders (SOC: 10029205)** | | | | | |
| Allodynia (PT: 10053552) | 6 | 6.58 (2.93-14.77) | 6.58 (27.80) | 2.69 (0.75) | 6.46 (2.88) |
| Hypoglycaemic unconsciousness (PT: 10065981) | 4 | 9.19 (3.40-24.84) | 9.19 (28.36) | 3.16 (0.47) | 8.96 (3.31) |

For tirzepatide, only the reports taking 2.5 mg dose were included. **Abbreviations:** PT, preferred term; ROR, reporting odds ratio; CI, confidence interval; PRR, proportional reporting ratio; χ2, chi-squared; IC, information component; IC025, lower limit of 95% confidence interval of IC; EBGM, empirical Bayesian geometric mean; EBGM05, lower limit of 95% confidence interval of EBGM.

## Supplementary Table S17. Number and signal strength of tirzepatide-related signals at the PT level from the FAERS database based on reports taking 5.0 mg dose.

| **PT** | **Number** | **ROR (95% CI)** | **PRR (χ2)** | **IC (IC025)** | **EBGM (EBGM05)** |  |
| --- | --- | --- | --- | --- | --- | --- |
| **General disorders and administration site conditions (SOC: 10018065)** | | | | | | |
| Injection site pain (PT: 10022086) | 726 | 11.23 (10.42-12.11) | 10.70 (6332.59) | 3.40 (3.27) | 10.57 (9.81) |  |
| Injection site haemorrhage (PT: 10022067) | 412 | 23.13 (20.95-25.55) | 22.48 (8241.29) | 4.45 (4.24) | 21.91 (19.84) |  |
| Injection site erythema (PT: 10022061) | 287 | 14.11 (12.54-15.88) | 13.84 (3367.73) | 3.77 (3.53) | 13.63 (12.11) |  |
| Injection site bruising (PT: 10022052) | 208 | 13.61 (11.85-15.62) | 13.42 (2355.15) | 3.72 (3.44) | 13.22 (11.52) |  |
| Injection site pruritus (PT: 10022093) | 163 | 13.55 (11.60-15.83) | 13.40 (1842.28) | 3.72 (3.39) | 13.20 (11.30) |  |
| Injection site mass (PT: 10022081) | 124 | 14.22 (11.90-17.00) | 14.11 (1485.43) | 3.80 (3.39) | 13.88 (11.62) |  |
| Injection site injury (PT: 10066083) | 104 | 63.41 (51.91-77.47) | 62.95 (5886.59) | 5.87 (4.95) | 58.51 (47.89) |  |
| Injection site swelling (PT: 10053425) | 100 | 6.28 (5.15-7.65) | 6.24 (437.03) | 2.63 (2.27) | 6.20 (5.09) |  |
| Injection site rash (PT: 10022094) | 96 | 14.18 (11.58-17.37) | 14.09 (1148.43) | 3.79 (3.32) | 13.87 (11.33) |  |
| Injection site urticaria (PT: 10022107) | 90 | 15.91 (12.91-19.61) | 15.81 (1225.62) | 3.96 (3.44) | 15.53 (12.60) |  |
| Injury associated with device (PT: 10069803) | 85 | 13.07 (10.54-16.20) | 13.00 (927.02) | 3.68 (3.18) | 12.81 (10.33) |  |
| Injection site reaction (PT: 10022095) | 60 | 5.41 (4.20-6.98) | 5.39 (213.44) | 2.42 (1.95) | 5.36 (4.16) |  |
| Injection site warmth (PT: 10022112) | 29 | 11.40 (7.90-16.45) | 11.38 (270.72) | 3.49 (2.53) | 11.23 (7.78) |  |
| Injection site irritation (PT: 10022079) | 21 | 11.21 (7.29-17.25) | 11.20 (192.40) | 3.47 (2.30) | 11.06 (7.19) |  |
| Injection site hypersensitivity (PT: 10022071) | 16 | 20.45 (12.45-33.60) | 20.43 (288.48) | 4.32 (2.53) | 19.96 (12.15) |  |
| Injection site discomfort (PT: 10054266) | 14 | 5.38 (3.18-9.10) | 5.38 (49.54) | 2.42 (1.30) | 5.35 (3.16) |  |
| Injection site vesicles (PT: 10022111) | 12 | 7.61 (4.31-13.45) | 7.61 (68.25) | 2.92 (1.52) | 7.55 (4.27) |  |
| Injection site induration (PT: 10022075) | 10 | 5.54 (2.97-10.32) | 5.54 (36.95) | 2.46 (1.09) | 5.51 (2.96) |  |
| Injection site inflammation (PT: 10022078) | 10 | 9.18 (4.92-17.13) | 9.17 (72.03) | 3.18 (1.51) | 9.08 (4.87) |  |
| Injection site coldness (PT: 10050082) | 9 | 38.21 (19.58-74.58) | 38.19 (311.36) | 5.19 (2.07) | 36.53 (18.72) |  |
| Injection site hypoaesthesia (PT: 10074586) | 6 | 16.09 (7.17-36.10) | 16.08 (83.22) | 3.98 (1.24) | 15.79 (7.04) |  |
| Injection site indentation (PT: 10079277) | 4 | 5.70 (2.13-15.24) | 5.70 (15.39) | 2.50 (0.25) | 5.66 (2.12) |  |
| Injection site paraesthesia (PT: 10022088) | 3 | 7.05 (2.26-21.95) | 7.04 (15.42) | 2.81 (0.03) | 6.99 (2.24) |  |
| **Gastrointestinal disorders (SOC: 10017947)** | | | | | | |
| Nausea (PT: 10028813) | 596 | 3.85 (3.54-4.18) | 3.73 (1197.43) | 1.89 (1.77) | 3.71 (3.42) |  |
| Vomiting (PT: 10047700) | 267 | 2.92 (2.59-3.30) | 2.88 (329.78) | 1.53 (1.34) | 2.88 (2.55) |  |
| Constipation (PT: 10010774) | 184 | 3.77 (3.26-4.36) | 3.73 (367.33) | 1.89 (1.66) | 3.72 (3.21) |  |
| Eructation (PT: 10015137) | 110 | 28.72 (23.73-34.76) | 28.51 (2821.58) | 4.79 (4.20) | 27.58 (22.78) |  |
| Pancreatitis (PT: 10033645) | 66 | 8.00 (6.27-10.20) | 7.97 (398.48) | 2.98 (2.49) | 7.90 (6.20) |  |
| Flatulence (PT: 10016766) | 60 | 5.37 (4.16-6.92) | 5.35 (210.91) | 2.41 (1.94) | 5.32 (4.13) |  |
| Impaired gastric emptying (PT: 10021518) | 28 | 10.36 (7.14-15.05) | 10.35 (233.47) | 3.35 (2.42) | 10.23 (7.04) |  |
| Breath odour (PT: 10006326) | 3 | 7.48 (2.40-23.30) | 7.47 (16.67) | 2.89 (0.06) | 7.42 (2.38) |  |
| **Metabolism and nutrition disorders (SOC: 10027433)** | | | | | | |
| Dehydration (PT: 10012174) | 69 | 2.92 (2.31-3.70) | 2.91 (86.54) | 1.54 (1.15) | 2.91 (2.29) |  |
| Feeding disorder (PT: 10061148) | 37 | 6.41 (4.64-8.87) | 6.40 (167.35) | 2.67 (2.01) | 6.36 (4.60) |  |
| Food craving (PT: 10056465) | 17 | 15.46 (9.56-24.99) | 15.44 (225.35) | 3.92 (2.40) | 15.17 (9.39) |  |
| Fluid intake reduced (PT: 10056291) | 9 | 8.18 (4.24-15.77) | 8.17 (56.11) | 3.02 (1.33) | 8.10 (4.20) |  |
| Ketoacidosis (PT: 10023379) | 6 | 4.78 (2.14-10.66) | 4.77 (17.80) | 2.25 (0.53) | 4.75 (2.13) |  |
| **Investigations (SOC: 10022891)** | | | | | | |
| Lipase increased (PT: 10024574) | 16 | 12.96 (7.91-21.24) | 12.95 (173.62) | 3.67 (2.21) | 12.76 (7.78) |  |
| Amylase increased (PT: 10002016) | 6 | 7.54 (3.37-16.84) | 7.53 (33.68) | 2.90 (0.86) | 7.47 (3.34) |  |
| **Reproductive system and breast disorders (SOC: 10038604)** | | | | | | |
| Menstrual disorder (PT: 10027327) | 8 | 6.00 (2.99-12.03) | 6.00 (33.06) | 2.58 (0.98) | 5.96 (2.97) |  |
| Postmenopausal haemorrhage (PT: 10055870) | 6 | 11.37 (5.08-25.46) | 11.37 (55.96) | 3.49 (1.09) | 11.23 (5.01) |  |
| **Respiratory, thoracic and mediastinal disorders (SOC: 10038738)** | | | | | | |
| Hiccups (PT: 10020039) | 7 | 4.43 (2.11-9.31) | 4.43 (18.48) | 2.14 (0.60) | 4.41 (2.10) |  |
| **Nervous system disorders (SOC: 10029205)** | | | | | | |
| Ophthalmic migraine (PT: 10030867) | 3 | 7.96 (2.55-24.83) | 7.96 (18.09) | 2.98 (0.08) | 7.89 (2.53) |  |

For tirzepatide, only the reports taking 5.0 mg dose were included. **Abbreviations:** PT, preferred term; ROR, reporting odds ratio; CI, confidence interval; PRR, proportional reporting ratio; χ2, chi-squared; IC, information component; IC025, lower limit of 95% confidence interval of IC; EBGM, empirical Bayesian geometric mean; EBGM05, lower limit of 95% confidence interval of EBGM.

## Supplementary Table S18. Number and signal strength of tirzepatide-related signals at the PT level from the FAERS database based on reports taking 7.5 mg dose.

| **PT** | **Number** | **ROR (95% CI)** | **PRR (χ2)** | **IC (IC025)** | **EBGM (EBGM05)** |
| --- | --- | --- | --- | --- | --- |
| **General disorders and administration site conditions (SOC: 10018065)** | | | | | |
| Injection site pain (PT: 10022086) | 322 | 9.75 (8.72-10.91) | 9.35 (2400.38) | 3.22 (3.02) | 9.31 (8.32) |
| Injection site haemorrhage (PT: 10022067) | 182 | 19.91 (17.17-23.08) | 19.42 (3146.19) | 4.26 (3.91) | 19.20 (16.56) |
| Injection site erythema (PT: 10022061) | 151 | 14.64 (12.45-17.21) | 14.35 (1860.94) | 3.83 (3.47) | 14.23 (12.10) |
| Injection site bruising (PT: 10022052) | 95 | 12.21 (9.97-14.97) | 12.06 (957.79) | 3.58 (3.13) | 11.98 (9.78) |
| Injection site pruritus (PT: 10022093) | 89 | 14.59 (11.83-18.00) | 14.42 (1102.90) | 3.84 (3.33) | 14.30 (11.59) |
| Injection site injury (PT: 10066083) | 58 | 67.95 (52.20-88.45) | 67.40 (3642.69) | 6.02 (4.57) | 64.74 (49.74) |
| Injection site rash (PT: 10022094) | 53 | 15.44 (11.77-20.25) | 15.33 (703.54) | 3.93 (3.19) | 15.19 (11.58) |
| Injection site swelling (PT: 10053425) | 52 | 6.46 (4.91-8.49) | 6.42 (237.12) | 2.68 (2.14) | 6.40 (4.87) |
| Injection site urticaria (PT: 10022107) | 42 | 14.58 (10.75-19.78) | 14.50 (523.47) | 3.85 (3.01) | 14.38 (10.60) |
| Injury associated with device (PT: 10069803) | 41 | 12.41 (9.12-16.89) | 12.35 (424.45) | 3.62 (2.82) | 12.26 (9.01) |
| Injection site mass (PT: 10022081) | 39 | 8.75 (6.38-12.00) | 8.71 (264.77) | 3.12 (2.40) | 8.66 (6.32) |
| Injection site reaction (PT: 10022095) | 27 | 4.81 (3.30-7.03) | 4.80 (81.06) | 2.26 (1.53) | 4.79 (3.28) |
| Injection site irritation (PT: 10022079) | 18 | 19.06 (11.97-30.36) | 19.02 (303.74) | 4.23 (2.61) | 18.81 (11.81) |
| Injection site hypersensitivity (PT: 10022071) | 8 | 20.05 (9.98-40.29) | 20.03 (142.89) | 4.31 (1.71) | 19.80 (9.86) |
| Injection site discomfort (PT: 10054266) | 7 | 5.32 (2.53-11.18) | 5.32 (24.47) | 2.41 (0.76) | 5.30 (2.52) |
| Injection site warmth (PT: 10022112) | 6 | 4.62 (2.07-10.31) | 4.62 (16.98) | 2.21 (0.51) | 4.61 (2.07) |
| Injection site vesicles (PT: 10022111) | 6 | 7.52 (3.37-16.78) | 7.52 (33.75) | 2.90 (0.86) | 7.49 (3.36) |
| Injection site coldness (PT: 10050082) | 5 | 41.29 (16.99-100.35) | 41.26 (191.56) | 5.33 (1.22) | 40.26 (16.57) |
| Injection site inflammation (PT: 10022078) | 5 | 9.06 (3.76-21.83) | 9.06 (35.64) | 3.17 (0.77) | 9.01 (3.74) |
| Injection site induration (PT: 10022075) | 5 | 5.48 (2.28-13.20) | 5.48 (18.25) | 2.45 (0.47) | 5.46 (2.27) |
| Injection site paraesthesia (PT: 10022088) | 3 | 13.99 (4.49-43.60) | 13.98 (35.86) | 3.79 (0.27) | 13.87 (4.45) |
| **Gastrointestinal disorders (SOC: 10017947)** | | | | | |
| Nausea (PT: 10028813) | 301 | 3.85 (3.43-4.32) | 3.73 (606.55) | 1.90 (1.71) | 3.72 (3.32) |
| Vomiting (PT: 10047700) | 124 | 2.68 (2.25-3.21) | 2.65 (128.54) | 1.41 (1.13) | 2.65 (2.22) |
| Constipation (PT: 10010774) | 81 | 3.28 (2.63-4.08) | 3.25 (126.51) | 1.70 (1.34) | 3.25 (2.61) |
| Eructation (PT: 10015137) | 47 | 23.86 (17.87-31.85) | 23.71 (1007.70) | 4.55 (3.57) | 23.38 (17.51) |
| Pancreatitis (PT: 10033645) | 36 | 8.63 (6.21-11.98) | 8.59 (240.30) | 3.10 (2.35) | 8.55 (6.16) |
| Flatulence (PT: 10016766) | 29 | 5.13 (3.56-7.39) | 5.11 (95.78) | 2.35 (1.64) | 5.10 (3.54) |
| Impaired gastric emptying (PT: 10021518) | 20 | 14.66 (9.43-22.78) | 14.62 (251.52) | 3.86 (2.51) | 14.50 (9.33) |
| **Metabolism and nutrition disorders (SOC: 10027433)** | | | | | |
| Feeding disorder (PT: 10061148) | 19 | 6.52 (4.15-10.23) | 6.50 (88.11) | 2.70 (1.70) | 6.48 (4.13) |
| Food craving (PT: 10056465) | 5 | 8.90 (3.70-21.45) | 8.90 (34.86) | 3.15 (0.76) | 8.85 (3.68) |
| Fluid intake reduced (PT: 10056291) | 4 | 7.18 (2.69-19.17) | 7.17 (21.16) | 2.84 (0.39) | 7.15 (2.68) |
| **Hepatobiliary disorders (SOC: 10019805)** | | | | | |
| Cholecystitis (PT: 10008612) | 7 | 7.26 (3.46-15.27) | 7.26 (37.61) | 2.85 (1.00) | 7.23 (3.44) |
| Gallbladder disorder (PT: 10017626) | 7 | 5.37 (2.56-11.29) | 5.37 (24.81) | 2.42 (0.77) | 5.35 (2.55) |
| **Investigations (SOC: 10022891)** | | | | | |
| Lipase increased (PT: 10024574) | 9 | 14.37 (7.45-27.72) | 14.36 (110.87) | 3.83 (1.70) | 14.24 (7.38) |
| **Nervous system disorders (SOC: 10029205)** | | | | | |
| Allodynia (PT: 10053552) | 4 | 22.56 (8.41-60.53) | 22.54 (81.23) | 4.48 (0.78) | 22.25 (8.29) |

For tirzepatide, only the reports taking 7.5 mg dose were included. **Abbreviations:** PT, preferred term; ROR, reporting odds ratio; CI, confidence interval; PRR, proportional reporting ratio; χ2, chi-squared; IC, information component; IC025, lower limit of 95% confidence interval of IC; EBGM, empirical Bayesian geometric mean; EBGM05, lower limit of 95% confidence interval of EBGM.

## Supplementary Table S19. Number and signal strength of tirzepatide-related signals at the PT level from the FAERS database based on reports with diabetes mellitus indication.

| **PT** | **Number** | **ROR (95% CI)** | **PRR (χ2)** | **IC (IC025)** | **EBGM (EBGM05)** |
| --- | --- | --- | --- | --- | --- |
| **General disorders and administration site conditions (SOC: 10018065)** | | | | | |
| Injection site pain (PT: 10022086) | 1543 | 13.33 (12.65-14.05) | 12.59 (16064.47) | 3.62 (3.53) | 12.25 (11.63) |
| Injection site haemorrhage (PT: 10022067) | 646 | 19.68 (18.17-21.32) | 19.21 (10676.46) | 4.20 (4.05) | 18.41 (17.00) |
| Injection site erythema (PT: 10022061) | 551 | 14.98 (13.74-16.32) | 14.68 (6793.43) | 3.83 (3.67) | 14.21 (13.04) |
| Injection site bruising (PT: 10022052) | 326 | 11.51 (10.31-12.86) | 11.38 (3008.21) | 3.47 (3.27) | 11.10 (9.94) |
| Injection site pruritus (PT: 10022093) | 312 | 14.25 (12.72-15.96) | 14.09 (3672.88) | 3.77 (3.55) | 13.66 (12.19) |
| Injection site mass (PT: 10022081) | 227 | 14.16 (12.40-16.18) | 14.05 (2662.94) | 3.77 (3.49) | 13.62 (11.93) |
| Injection site rash (PT: 10022094) | 175 | 14.27 (12.27-16.60) | 14.18 (2074.87) | 3.78 (3.46) | 13.75 (11.82) |
| Injection site swelling (PT: 10053425) | 165 | 5.77 (4.95-6.73) | 5.74 (637.90) | 2.50 (2.24) | 5.68 (4.87) |
| Injection site urticaria (PT: 10022107) | 124 | 11.99 (10.02-14.34) | 11.94 (1208.29) | 3.54 (3.16) | 11.63 (9.73) |
| Injection site reaction (PT: 10022095) | 112 | 5.47 (4.54-6.60) | 5.45 (402.51) | 2.43 (2.10) | 5.40 (4.48) |
| Injection site injury (PT: 10066083) | 107 | 34.52 (28.33-42.06) | 34.38 (3203.73) | 4.99 (4.34) | 31.83 (26.13) |
| Injury associated with device (PT: 10069803) | 85 | 7.35 (5.93-9.11) | 7.33 (456.98) | 2.85 (2.44) | 7.22 (5.83) |
| Injection site irritation (PT: 10022079) | 47 | 14.59 (10.90-19.52) | 14.56 (573.67) | 3.82 (3.04) | 14.10 (10.54) |
| Injection site discomfort (PT: 10054266) | 40 | 8.49 (6.21-11.61) | 8.48 (258.67) | 3.06 (2.36) | 8.33 (6.09) |
| Injection site warmth (PT: 10022112) | 36 | 7.60 (5.46-10.57) | 7.59 (202.29) | 2.90 (2.19) | 7.47 (5.37) |
| Injection site coldness (PT: 10050082) | 25 | 60.54 (39.79-92.11) | 60.48 (1276.90) | 5.73 (3.54) | 52.93 (34.79) |
| Injection site hypersensitivity (PT: 10022071) | 24 | 16.69 (11.09-25.10) | 16.67 (339.98) | 4.01 (2.74) | 16.07 (10.68) |
| Injection site paraesthesia (PT: 10022088) | 22 | 28.81 (18.70-44.39) | 28.79 (551.94) | 4.75 (3.04) | 26.99 (17.52) |
| Injection site vesicles (PT: 10022111) | 19 | 6.71 (4.26-10.56) | 6.71 (90.79) | 2.73 (1.72) | 6.62 (4.20) |
| Injection site discolouration (PT: 10051572) | 18 | 4.11 (2.58-6.53) | 4.10 (41.86) | 2.03 (1.14) | 4.07 (2.56) |
| Injection site inflammation (PT: 10022078) | 16 | 8.03 (4.89-13.16) | 8.02 (96.49) | 2.98 (1.78) | 7.89 (4.81) |
| Injection site induration (PT: 10022075) | 15 | 4.39 (2.64-7.30) | 4.39 (38.85) | 2.12 (1.12) | 4.35 (2.62) |
| Injection site indentation (PT: 10079277) | 10 | 8.15 (4.36-15.23) | 8.14 (61.46) | 3.00 (1.41) | 8.01 (4.28) |
| Injection site laceration (PT: 10067253) | 6 | 34.21 (14.88-78.65) | 34.20 (178.70) | 4.99 (1.42) | 31.68 (13.78) |
| Injection site scar (PT: 10059009) | 6 | 5.03 (2.25-11.26) | 5.03 (19.16) | 2.32 (0.57) | 4.99 (2.23) |
| Injection site hypoaesthesia (PT: 10074586) | 4 | 5.59 (2.08-14.98) | 5.59 (14.86) | 2.47 (0.23) | 5.52 (2.06) |
| Thirst decreased (PT: 10050200) | 4 | 16.01 (5.90-43.46) | 16.00 (54.18) | 3.95 (0.67) | 15.45 (5.69) |
| **Gastrointestinal disorders (SOC: 10017947)** | | | | | |
| Nausea (PT: 10028813) | 1443 | 5.14 (4.87-5.42) | 4.91 (4489.21) | 2.28 (2.20) | 4.86 (4.61) |
| Diarrhoea (PT: 10012735) | 809 | 2.97 (2.77-3.19) | 2.91 (1019.17) | 1.54 (1.43) | 2.90 (2.70) |
| Vomiting (PT: 10047700) | 623 | 3.71 (3.43-4.02) | 3.65 (1193.84) | 1.86 (1.73) | 3.62 (3.34) |
| Constipation (PT: 10010774) | 470 | 5.20 (4.74-5.70) | 5.12 (1544.83) | 2.34 (2.19) | 5.07 (4.63) |
| Eructation (PT: 10015137) | 267 | 38.81 (34.22-44.02) | 38.42 (8910.64) | 5.14 (4.78) | 35.26 (31.08) |
| Abdominal discomfort (PT: 10000059) | 219 | 2.91 (2.55-3.32) | 2.89 (270.18) | 1.53 (1.32) | 2.88 (2.52) |
| Flatulence (PT: 10016766) | 165 | 7.97 (6.83-9.30) | 7.93 (980.68) | 2.96 (2.68) | 7.80 (6.68) |
| Pancreatitis (PT: 10033645) | 141 | 9.27 (7.84-10.96) | 9.23 (1012.23) | 3.18 (2.85) | 9.05 (7.65) |
| Impaired gastric emptying (PT: 10021518) | 117 | 23.90 (19.83-28.81) | 23.80 (2417.29) | 4.50 (3.98) | 22.56 (18.72) |
| Intestinal obstruction (PT: 10022687) | 43 | 2.74 (2.03-3.70) | 2.73 (47.02) | 1.45 (0.95) | 2.72 (2.02) |
| Pancreatitis acute (PT: 10033647) | 32 | 4.22 (2.98-5.98) | 4.21 (77.67) | 2.06 (1.43) | 4.18 (2.95) |
| Small intestinal obstruction (PT: 10041101) | 15 | 3.56 (2.14-5.91) | 3.55 (27.31) | 1.82 (0.88) | 3.53 (2.12) |
| Vomiting projectile (PT: 10047708) | 8 | 7.91 (3.93-15.92) | 7.91 (47.37) | 2.96 (1.18) | 7.78 (3.86) |
| Regurgitation (PT: 10067171) | 6 | 4.86 (2.17-10.87) | 4.86 (18.17) | 2.27 (0.54) | 4.81 (2.15) |
| Abdominal rigidity (PT: 10000090) | 5 | 4.99 (2.07-12.05) | 4.99 (15.76) | 2.31 (0.39) | 4.94 (2.05) |
| **Metabolism and nutrition disorders (SOC: 10027433)** | | | | | |
| Dehydration (PT: 10012174) | 170 | 3.91 (3.36-4.55) | 3.89 (362.11) | 1.95 (1.70) | 3.86 (3.32) |
| Feeding disorder (PT: 10061148) | 67 | 6.23 (4.90-7.94) | 6.22 (289.34) | 2.62 (2.16) | 6.14 (4.83) |
| Food craving (PT: 10056465) | 40 | 19.52 (14.21-26.81) | 19.49 (670.33) | 4.22 (3.24) | 18.66 (13.59) |
| Hypophagia (PT: 10063743) | 35 | 3.71 (2.66-5.17) | 3.70 (68.47) | 1.88 (1.29) | 3.68 (2.64) |
| Diabetic ketoacidosis (PT: 10012671) | 26 | 3.44 (2.34-5.06) | 3.44 (44.57) | 1.77 (1.09) | 3.42 (2.32) |
| Fluid intake reduced (PT: 10056291) | 15 | 7.36 (4.41-12.26) | 7.35 (80.90) | 2.86 (1.65) | 7.24 (4.35) |
| Ketoacidosis (PT: 10023379) | 11 | 4.65 (2.57-8.43) | 4.65 (31.19) | 2.21 (0.99) | 4.61 (2.55) |
| Food aversion (PT: 10049238) | 6 | 6.94 (3.10-15.54) | 6.94 (29.98) | 2.77 (0.80) | 6.84 (3.05) |
| **Hepatobiliary disorders (SOC: 10019805)** | | | | | |
| Cholelithiasis (PT: 10008629) | 35 | 3.61 (2.59-5.03) | 3.60 (65.31) | 1.84 (1.26) | 3.58 (2.57) |
| Gallbladder disorder (PT: 10017626) | 19 | 3.97 (2.53-6.24) | 3.97 (41.77) | 1.98 (1.13) | 3.94 (2.51) |
| Cholecystitis (PT: 10008612) | 18 | 5.00 (3.14-7.95) | 4.99 (56.81) | 2.31 (1.37) | 4.95 (3.11) |
| Cholecystitis acute (PT: 10008614) | 11 | 6.65 (3.66-12.06) | 6.64 (51.91) | 2.71 (1.32) | 6.55 (3.61) |
| Cholangitis acute (PT: 10008605) | 5 | 15.19 (6.22-37.08) | 15.19 (63.93) | 3.88 (0.96) | 14.69 (6.02) |
| **Investigations (SOC: 10022891)** | | | | | |
| Lipase increased (PT: 10024574) | 29 | 12.73 (8.80-18.42) | 12.72 (303.79) | 3.63 (2.63) | 12.37 (8.55) |
| Glomerular filtration rate decreased (PT: 10018358) | 22 | 3.62 (2.38-5.51) | 3.62 (41.34) | 1.85 (1.09) | 3.60 (2.36) |
| Amylase increased (PT: 10002016) | 7 | 4.60 (2.19-9.70) | 4.60 (19.52) | 2.19 (0.63) | 4.56 (2.17) |
| Pancreatic enzymes increased (PT: 10061900) | 5 | 9.77 (4.02-23.72) | 9.77 (38.45) | 3.26 (0.78) | 9.57 (3.94) |
| Gastric pH decreased (PT: 10060041) | 4 | 10.34 (3.83-27.89) | 10.34 (32.92) | 3.34 (0.53) | 10.11 (3.75) |
| Blood calcitonin increased (PT: 10005390) | 3 | 48.02 (14.53-158.66) | 48.01 (123.82) | 5.43 (0.36) | 43.15 (13.06) |
| Protein urine (PT: 10037018) | 3 | 8.38 (2.67-26.28) | 8.38 (19.11) | 3.04 (0.09) | 8.23 (2.63) |
| **Skin and subcutaneous tissue disorders (SOC: 10040785)** | | | | | |
| Sensitive skin (PT: 10081765) | 16 | 3.59 (2.19-5.87) | 3.59 (29.58) | 1.83 (0.93) | 3.56 (2.18) |
| Skin odour abnormal (PT: 10040904) | 6 | 5.08 (2.27-11.35) | 5.07 (19.39) | 2.33 (0.57) | 5.03 (2.25) |
| Skin laxity (PT: 10057064) | 3 | 11.35 (3.60-35.74) | 11.35 (27.56) | 3.47 (0.18) | 11.07 (3.52) |
| **Psychiatric disorders (SOC: 10037175)** | | | | | |
| Sleep disorder due to general medical condition, insomnia type (PT: 10040986) | 24 | 4.92 (3.29-7.36) | 4.92 (74.03) | 2.28 (1.50) | 4.87 (3.26) |
| **Musculoskeletal and connective tissue disorders (SOC: 10028395)** | | | | | |
| Muscle atrophy (PT: 10028289) | 20 | 3.92 (2.52-6.09) | 3.92 (43.11) | 1.96 (1.14) | 3.89 (2.51) |
| **Endocrine disorders (SOC: 10014698)** | | | | | |
| Thyroid mass (PT: 10058900) | 9 | 4.53 (2.35-8.74) | 4.53 (24.48) | 2.17 (0.82) | 4.49 (2.33) |
| Goitre (PT: 10018498) | 6 | 5.13 (2.29-11.47) | 5.13 (19.69) | 2.34 (0.58) | 5.08 (2.27) |
| **Nervous system disorders (SOC: 10029205)** | | | | | |
| Allodynia (PT: 10053552) | 7 | 10.71 (5.06-22.69) | 10.71 (60.07) | 3.39 (1.22) | 10.47 (4.94) |
| Hypoglycaemic unconsciousness (PT: 10065981) | 4 | 12.33 (4.56-33.34) | 12.33 (40.44) | 3.59 (0.59) | 12.00 (4.44) |
| Wernicke's encephalopathy (PT: 10047911) | 4 | 19.13 (7.02-52.14) | 19.13 (65.72) | 4.20 (0.71) | 18.33 (6.73) |
| **Eye disorders (SOC: 10015919)** | | | | | |
| Retinopathy (PT: 10038923) | 9 | 7.69 (3.98-14.88) | 7.69 (51.43) | 2.92 (1.27) | 7.57 (3.91) |
| **Infections and infestations (SOC: 10021881)** | | | | | |
| Injection site infection (PT: 10022076) | 7 | 6.86 (3.25-14.47) | 6.85 (34.43) | 2.76 (0.94) | 6.76 (3.20) |
| **Reproductive system and breast disorders (SOC: 10038604)** | | | | | |
| Oligomenorrhoea (PT: 10030295) | 5 | 11.43 (4.70-27.80) | 11.43 (46.32) | 3.48 (0.85) | 11.15 (4.59) |

For tirzepatide, only the reports with “type 2 diabetes mellitus” and “diabetes mellitus” indications were included. **Abbreviations:** PT, preferred term; ROR, reporting odds ratio; CI, confidence interval; PRR, proportional reporting ratio; χ2, chi-squared; IC, information component; IC025, lower limit of 95% confidence interval of IC; EBGM, empirical Bayesian geometric mean; EBGM05, lower limit of 95% confidence interval of EBGM.

## Supplementary Table S20. Number and signal strength of tirzepatide-related signals at the PT level from the FAERS database based on reports with weight control indication.

| **PT** | **Number** | **ROR (95% CI)** | **PRR (χ2)** | **IC (IC025)** | **EBGM (EBGM05)** |
| --- | --- | --- | --- | --- | --- |
| **General disorders and administration site conditions (SOC: 10018065)** | | | | | |
| Injection site pain (PT: 10022086) | 775 | 11.76 (10.93-12.65) | 11.18 (7111.85) | 3.46 (3.34) | 11.03 (10.25) |
| Injection site haemorrhage (PT: 10022067) | 335 | 17.88 (16.02-19.95) | 17.49 (5094.54) | 4.10 (3.87) | 17.11 (15.33) |
| Injection site erythema (PT: 10022061) | 289 | 13.84 (12.30-15.56) | 13.58 (3313.03) | 3.74 (3.51) | 13.36 (11.88) |
| Injection site bruising (PT: 10022052) | 215 | 13.53 (11.81-15.50) | 13.34 (2413.97) | 3.71 (3.44) | 13.12 (11.46) |
| Injection site pruritus (PT: 10022093) | 167 | 13.46 (11.54-15.70) | 13.32 (1870.95) | 3.71 (3.39) | 13.10 (11.23) |
| Injection site rash (PT: 10022094) | 91 | 13.09 (10.64-16.12) | 13.02 (992.79) | 3.68 (3.20) | 12.81 (10.41) |
| Injection site injury (PT: 10066083) | 91 | 52.14 (42.13-64.52) | 51.81 (4240.94) | 5.60 (4.69) | 48.51 (39.20) |
| Injection site swelling (PT: 10053425) | 84 | 5.23 (4.22-6.49) | 5.21 (283.89) | 2.37 (1.99) | 5.18 (4.18) |
| Injection site mass (PT: 10022081) | 74 | 8.07 (6.41-10.15) | 8.03 (451.03) | 2.99 (2.53) | 7.96 (6.32) |
| Injection site urticaria (PT: 10022107) | 71 | 12.17 (9.62-15.39) | 12.11 (712.48) | 3.58 (3.03) | 11.93 (9.43) |
| Injection site reaction (PT: 10022095) | 57 | 4.96 (3.82-6.44) | 4.95 (178.51) | 2.30 (1.82) | 4.92 (3.79) |
| Injury associated with device (PT: 10069803) | 54 | 8.33 (6.37-10.90) | 8.30 (343.17) | 3.04 (2.47) | 8.22 (6.28) |
| Injection site irritation (PT: 10022079) | 27 | 14.81 (10.12-21.69) | 14.79 (340.37) | 3.86 (2.74) | 14.52 (9.92) |
| Injection site discomfort (PT: 10054266) | 26 | 9.83 (6.68-14.48) | 9.82 (203.25) | 3.28 (2.32) | 9.70 (6.59) |
| Injection site warmth (PT: 10022112) | 24 | 9.03 (6.04-13.52) | 9.02 (169.14) | 3.16 (2.18) | 8.92 (5.97) |
| Injection site coldness (PT: 10050082) | 16 | 66.06 (39.61-110.16) | 65.98 (940.84) | 5.92 (3.02) | 60.71 (36.40) |
| Injection site inflammation (PT: 10022078) | 13 | 11.66 (6.74-20.17) | 11.65 (124.62) | 3.52 (1.94) | 11.49 (6.64) |
| Injection site hypersensitivity (PT: 10022071) | 12 | 14.67 (8.28-25.98) | 14.66 (149.81) | 3.85 (2.02) | 14.40 (8.13) |
| Injection site paraesthesia (PT: 10022088) | 11 | 24.98 (13.69-45.55) | 24.96 (244.79) | 4.60 (2.19) | 24.18 (13.26) |
| Injection site induration (PT: 10022075) | 9 | 4.71 (2.44-9.07) | 4.70 (26.09) | 2.23 (0.86) | 4.68 (2.43) |
| Injection site vesicles (PT: 10022111) | 8 | 5.02 (2.50-10.06) | 5.02 (25.57) | 2.32 (0.82) | 4.99 (2.49) |
| Injection site indentation (PT: 10079277) | 6 | 8.70 (3.89-19.46) | 8.70 (40.40) | 3.11 (0.95) | 8.61 (3.85) |
| **Gastrointestinal disorders (SOC: 10017947)** | | | | | |
| Nausea (PT: 10028813) | 661 | 4.15 (3.84-4.49) | 4.01 (1500.97) | 2.00 (1.88) | 3.99 (3.69) |
| Vomiting (PT: 10047700) | 257 | 2.71 (2.40-3.07) | 2.68 (272.41) | 1.42 (1.23) | 2.68 (2.37) |
| Constipation (PT: 10010774) | 216 | 4.24 (3.71-4.85) | 4.19 (524.17) | 2.06 (1.84) | 4.18 (3.65) |
| Eructation (PT: 10015137) | 117 | 28.94 (24.04-34.83) | 28.71 (3014.08) | 4.79 (4.22) | 27.68 (23.00) |
| Flatulence (PT: 10016766) | 61 | 5.21 (4.05-6.71) | 5.19 (205.30) | 2.37 (1.91) | 5.16 (4.01) |
| Pancreatitis (PT: 10033645) | 29 | 3.35 (2.32-4.83) | 3.34 (47.48) | 1.74 (1.10) | 3.33 (2.31) |
| Impaired gastric emptying (PT: 10021518) | 16 | 5.57 (3.40-9.11) | 5.56 (59.45) | 2.47 (1.42) | 5.53 (3.38) |
| Vomiting projectile (PT: 10047708) | 9 | 16.00 (8.27-30.98) | 16.00 (123.87) | 3.97 (1.74) | 15.68 (8.10) |
| Gastrointestinal sounds abnormal (PT: 10067715) | 9 | 6.91 (3.58-13.32) | 6.90 (45.03) | 2.78 (1.19) | 6.85 (3.55) |
| Food poisoning (PT: 10016952) | 8 | 4.99 (2.49-10.01) | 4.99 (25.35) | 2.31 (0.82) | 4.96 (2.48) |
| Faecaloma (PT: 10056325) | 6 | 5.66 (2.54-12.65) | 5.66 (22.86) | 2.49 (0.66) | 5.63 (2.52) |
| Regurgitation (PT: 10067171) | 4 | 5.79 (2.16-15.48) | 5.79 (15.72) | 2.52 (0.26) | 5.75 (2.15) |
| Oesophageal rupture (PT: 10052211) | 4 | 25.97 (9.58-70.39) | 25.96 (92.78) | 4.65 (0.79) | 25.12 (9.27) |
| Breath odour (PT: 10006326) | 3 | 7.07 (2.27-22.03) | 7.06 (15.47) | 2.81 (0.03) | 7.01 (2.25) |
| **Metabolism and nutrition disorders (SOC: 10027433)** | | | | | |
| Feeding disorder (PT: 10061148) | 41 | 6.81 (5.00-9.26) | 6.79 (200.64) | 2.75 (2.12) | 6.74 (4.95) |
| Food craving (PT: 10056465) | 17 | 14.49 (8.96-23.41) | 14.47 (209.12) | 3.83 (2.35) | 14.21 (8.79) |
| Fluid intake reduced (PT: 10056291) | 8 | 6.98 (3.48-14.00) | 6.98 (40.58) | 2.79 (1.09) | 6.92 (3.45) |
| **Psychiatric disorders (SOC: 10037175)** | | | | | |
| Panic attack (PT: 10033664) | 22 | 3.32 (2.18-5.05) | 3.31 (35.41) | 1.72 (0.98) | 3.30 (2.17) |
| Sleep disorder due to general medical condition, insomnia type (PT: 10040986) | 16 | 5.86 (3.58-9.59) | 5.86 (63.96) | 2.54 (1.48) | 5.82 (3.56) |
| **Hepatobiliary disorders (SOC: 10019805)** | | | | | |
| Cholelithiasis (PT: 10008629) | 18 | 3.31 (2.09-5.27) | 3.31 (28.92) | 1.72 (0.89) | 3.30 (2.08) |
| Biliary colic (PT: 10004663) | 5 | 7.68 (3.18-18.54) | 7.68 (28.75) | 2.93 (0.67) | 7.61 (3.15) |
| **Investigations (SOC: 10022891)** | | | | | |
| Lipase increased (PT: 10024574) | 10 | 7.72 (4.14-14.39) | 7.71 (57.82) | 2.93 (1.38) | 7.64 (4.10) |
| Amylase increased (PT: 10002016) | 5 | 5.88 (2.44-14.18) | 5.88 (20.08) | 2.55 (0.51) | 5.84 (2.42) |
| Gastric pH decreased (PT: 10060041) | 4 | 18.55 (6.88-50.03) | 18.55 (64.79) | 4.18 (0.72) | 18.12 (6.72) |
| **Reproductive system and breast disorders (SOC: 10038604)** | | | | | |
| Postmenopausal haemorrhage (PT: 10055870) | 9 | 17.28 (8.92-33.47) | 17.27 (134.84) | 4.08 (1.78) | 16.90 (8.73) |
| Menstruation delayed (PT: 10027336) | 8 | 6.72 (3.35-13.48) | 6.72 (38.58) | 2.74 (1.06) | 6.67 (3.32) |
| **Skin and subcutaneous tissue disorders (SOC: 10040785)** | | | | | |
| Sensitive skin (PT: 10081765) | 11 | 4.41 (2.44-7.98) | 4.41 (28.84) | 2.13 (0.94) | 4.39 (2.43) |
| Skin laxity (PT: 10057064) | 3 | 20.36 (6.47-64.12) | 20.36 (53.75) | 4.31 (0.33) | 19.84 (6.30) |
| **Nervous system disorders (SOC: 10029205)** | | | | | |
| Allodynia (PT: 10053552) | 5 | 13.63 (5.63-33.00) | 13.62 (57.43) | 3.74 (0.94) | 13.40 (5.53) |
| **Ear and labyrinth disorders (SOC: 10013993)** | | | | | |
| Ear swelling (PT: 10014025) | 4 | 6.51 (2.43-17.41) | 6.50 (18.47) | 2.69 (0.33) | 6.46 (2.41) |
| **Respiratory, thoracic and mediastinal disorders (SOC: 10038738)** | | | | | |
| Pneumomediastinum (PT: 10050184) | 4 | 7.74 (2.89-20.72) | 7.74 (23.22) | 2.94 (0.42) | 7.67 (2.86) |

For tirzepatide, only the reports with “weight control” and “weight decreased” indications were included. **Abbreviations:** PT, preferred term; ROR, reporting odds ratio; CI, confidence interval; PRR, proportional reporting ratio; χ2, chi-squared; IC, information component; IC025, lower limit of 95% confidence interval of IC; EBGM, empirical Bayesian geometric mean; EBGM05, lower limit of 95% confidence interval of EBGM.

## Supplementary Table S21. Number and signal strength of tirzepatide-related signals at the PT level from the FAERS database based on reports in year 2022-2023.

| **PT** | **Number** | **ROR (95% CI)** | **PRR (χ2)** | **IC (IC025)** | **EBGM (EBGM05)** |
| --- | --- | --- | --- | --- | --- |
| **General disorders and administration site conditions (SOC: 10018065)** | | | | | |
| Injection site pain (PT: 10022086) | 2148 | 12.02 (11.50-12.56) | 11.43 (19748.38) | 3.46 (3.39) | 11.03 (10.55) |
| Injection site haemorrhage (PT: 10022067) | 1080 | 22.27 (20.92-23.71) | 21.69 (19845.51) | 4.34 (4.22) | 20.24 (19.01) |
| Injection site erythema (PT: 10022061) | 812 | 14.49 (13.49-15.56) | 14.21 (9517.70) | 3.76 (3.64) | 13.59 (12.66) |
| Injection site bruising (PT: 10022052) | 492 | 11.55 (10.55-12.65) | 11.42 (4503.66) | 3.46 (3.30) | 11.02 (10.07) |
| Injection site pruritus (PT: 10022093) | 475 | 14.34 (13.07-15.73) | 14.18 (5549.89) | 3.76 (3.59) | 13.56 (12.36) |
| Injection site mass (PT: 10022081) | 278 | 11.44 (10.14-12.90) | 11.37 (2529.59) | 3.46 (3.23) | 10.97 (9.73) |
| Injection site swelling (PT: 10053425) | 272 | 6.08 (5.39-6.86) | 6.05 (1123.19) | 2.57 (2.37) | 5.94 (5.27) |
| Injection site rash (PT: 10022094) | 261 | 13.97 (12.33-15.82) | 13.89 (2978.09) | 3.73 (3.48) | 13.29 (11.73) |
| Injection site urticaria (PT: 10022107) | 213 | 13.59 (11.84-15.59) | 13.52 (2358.87) | 3.70 (3.41) | 12.95 (11.29) |
| Injury associated with device (PT: 10069803) | 202 | 11.16 (9.69-12.85) | 11.11 (1789.01) | 3.42 (3.15) | 10.73 (9.32) |
| Injection site injury (PT: 10066083) | 186 | 42.35 (36.30-49.41) | 42.16 (6515.76) | 5.20 (4.73) | 36.88 (31.61) |
| Injection site reaction (PT: 10022095) | 159 | 5.10 (4.36-5.96) | 5.08 (512.42) | 2.32 (2.06) | 5.01 (4.28) |
| Injection site irritation (PT: 10022079) | 72 | 13.99 (11.04-17.73) | 13.97 (826.50) | 3.74 (3.17) | 13.36 (10.55) |
| Injection site warmth (PT: 10022112) | 57 | 7.98 (6.13-10.38) | 7.97 (338.08) | 2.96 (2.42) | 7.78 (5.98) |
| Injection site discomfort (PT: 10054266) | 47 | 6.45 (4.83-8.62) | 6.45 (211.52) | 2.66 (2.09) | 6.33 (4.74) |
| Injection site discolouration (PT: 10051572) | 31 | 4.70 (3.29-6.70) | 4.70 (88.75) | 2.21 (1.54) | 4.64 (3.25) |
| Injection site paraesthesia (PT: 10022088) | 29 | 25.90 (17.71-37.88) | 25.88 (636.24) | 4.57 (3.21) | 23.82 (16.29) |
| Injection site vesicles (PT: 10022111) | 27 | 6.09 (4.16-8.92) | 6.09 (112.50) | 2.58 (1.79) | 5.98 (4.09) |
| Injection site coldness (PT: 10050082) | 26 | 42.59 (28.21-64.31) | 42.56 (918.78) | 5.22 (3.40) | 37.19 (24.63) |
| Injection site hypersensitivity (PT: 10022071) | 23 | 10.45 (6.89-15.84) | 10.44 (189.48) | 3.34 (2.27) | 10.11 (6.67) |
| Injection site induration (PT: 10022075) | 21 | 4.12 (2.68-6.34) | 4.12 (48.93) | 2.03 (1.22) | 4.08 (2.65) |
| Injection site inflammation (PT: 10022078) | 19 | 6.20 (3.93-9.76) | 6.19 (80.98) | 2.60 (1.62) | 6.08 (3.86) |
| Injection site indentation (PT: 10079277) | 11 | 5.58 (3.07-10.13) | 5.58 (40.54) | 2.46 (1.15) | 5.49 (3.02) |
| Injection site laceration (PT: 10067253) | 9 | 36.32 (18.15-72.68) | 36.32 (274.31) | 5.02 (2.00) | 32.34 (16.16) |
| Injection site hypoaesthesia (PT: 10074586) | 9 | 8.57 (4.41-16.63) | 8.57 (58.40) | 3.06 (1.34) | 8.35 (4.30) |
| Injection site scab (PT: 10066210) | 7 | 14.53 (6.80-31.06) | 14.53 (83.95) | 3.79 (1.36) | 13.88 (6.49) |
| Thirst decreased (PT: 10050200) | 6 | 15.92 (7.00-36.22) | 15.92 (79.46) | 3.92 (1.20) | 15.13 (6.65) |
| Application site wound (PT: 10078380) | 3 | 21.49 (6.65-69.46) | 21.49 (54.51) | 4.33 (0.29) | 20.06 (6.20) |
| **Gastrointestinal disorders (SOC: 10017947)** | | | | | |
| Nausea (PT: 10028813) | 2296 | 5.37 (5.14-5.60) | 5.12 (7554.41) | 2.33 (2.27) | 5.04 (4.83) |
| Diarrhoea (PT: 10012735) | 1109 | 2.67 (2.52-2.84) | 2.63 (1117.85) | 1.38 (1.29) | 2.61 (2.46) |
| Vomiting (PT: 10047700) | 962 | 3.76 (3.52-4.00) | 3.69 (1873.68) | 1.87 (1.77) | 3.65 (3.43) |
| Constipation (PT: 10010774) | 646 | 4.72 (4.36-5.10) | 4.66 (1833.00) | 2.20 (2.08) | 4.60 (4.25) |
| Eructation (PT: 10015137) | 398 | 40.32 (36.29-44.79) | 39.92 (13259.98) | 5.14 (4.86) | 35.16 (31.65) |
| Abdominal discomfort (PT: 10000059) | 311 | 2.70 (2.41-3.02) | 2.69 (327.07) | 1.42 (1.24) | 2.67 (2.39) |
| Pancreatitis (PT: 10033645) | 220 | 9.60 (8.39-10.99) | 9.56 (1632.07) | 3.21 (2.96) | 9.28 (8.11) |
| Flatulence (PT: 10016766) | 195 | 6.23 (5.40-7.18) | 6.20 (833.80) | 2.61 (2.36) | 6.09 (5.29) |
| Impaired gastric emptying (PT: 10021518) | 108 | 14.60 (12.03-17.71) | 14.56 (1298.21) | 3.80 (3.35) | 13.90 (11.46) |
| Gastrointestinal sounds abnormal (PT: 10067715) | 25 | 7.26 (4.88-10.80) | 7.26 (131.54) | 2.83 (1.95) | 7.10 (4.77) |
| Small intestinal obstruction (PT: 10041101) | 21 | 3.27 (2.13-5.03) | 3.27 (32.75) | 1.70 (0.94) | 3.25 (2.11) |
| Vomiting projectile (PT: 10047708) | 10 | 6.41 (3.43-12.00) | 6.41 (44.65) | 2.65 (1.20) | 6.29 (3.36) |
| Abdominal rigidity (PT: 10000090) | 7 | 4.65 (2.21-9.82) | 4.65 (19.75) | 2.20 (0.63) | 4.59 (2.18) |
| **Metabolism and nutrition disorders (SOC: 10027433)** | | | | | |
| Dehydration (PT: 10012174) | 228 | 3.43 (3.01-3.91) | 3.41 (385.05) | 1.76 (1.55) | 3.38 (2.97) |
| Feeding disorder (PT: 10061148) | 120 | 7.45 (6.21-8.93) | 7.43 (651.14) | 2.86 (2.52) | 7.27 (6.06) |
| Food craving (PT: 10056465) | 47 | 15.55 (11.59-20.86) | 15.53 (606.15) | 3.89 (3.09) | 14.78 (11.02) |
| Fluid intake reduced (PT: 10056291) | 17 | 5.48 (3.39-8.86) | 5.48 (61.07) | 2.43 (1.43) | 5.39 (3.34) |
| Appetite disorder (PT: 10060961) | 17 | 3.41 (2.11-5.50) | 3.41 (28.59) | 1.76 (0.89) | 3.38 (2.09) |
| Food aversion (PT: 10049238) | 11 | 8.66 (4.75-15.78) | 8.66 (72.32) | 3.08 (1.53) | 8.43 (4.63) |
| Lack of satiety (PT: 10064720) | 4 | 23.88 (8.61-66.22) | 23.87 (80.92) | 4.47 (0.73) | 22.11 (7.97) |
| **Hepatobiliary disorders (SOC: 10019805)** | | | | | |
| Gallbladder disorder (PT: 10017626) | 51 | 7.08 (5.36-9.35) | 7.08 (259.69) | 2.79 (2.23) | 6.93 (5.25) |
| Cholelithiasis (PT: 10008629) | 49 | 3.37 (2.54-4.47) | 3.37 (80.70) | 1.74 (1.26) | 3.34 (2.52) |
| Biliary colic (PT: 10004663) | 9 | 5.25 (2.72-10.16) | 5.25 (30.42) | 2.37 (0.94) | 5.17 (2.68) |
| Cholangitis acute (PT: 10008605) | 4 | 8.13 (3.01-21.96) | 8.13 (24.31) | 2.99 (0.42) | 7.93 (2.94) |
| **Investigations (SOC: 10022891)** | | | | | |
| Lipase increased (PT: 10024574) | 36 | 10.46 (7.50-14.59) | 10.45 (296.82) | 3.34 (2.54) | 10.12 (7.25) |
| Pancreatic enzymes increased (PT: 10061900) | 12 | 15.56 (8.70-27.82) | 15.56 (155.03) | 3.89 (2.02) | 14.81 (8.28) |
| Amylase increased (PT: 10002016) | 9 | 3.99 (2.07-7.71) | 3.99 (19.90) | 1.98 (0.69) | 3.95 (2.05) |
| Thyroid hormones increased (PT: 10063161) | 7 | 7.35 (3.47-15.56) | 7.35 (37.42) | 2.85 (0.98) | 7.19 (3.39) |
| Gastric pH decreased (PT: 10060041) | 5 | 8.58 (3.52-20.88) | 8.58 (32.50) | 3.06 (0.71) | 8.36 (3.43) |
| Body mass index decreased (PT: 10005895) | 4 | 11.02 (4.06-29.92) | 11.02 (35.09) | 3.41 (0.54) | 10.65 (3.92) |
| Blood calcitonin increased (PT: 10005390) | 3 | 33.06 (10.01-109.23) | 33.06 (83.61) | 4.89 (0.32) | 29.74 (9.00) |
| **Reproductive system and breast disorders (SOC: 10038604)** | | | | | |
| Menstrual disorder (PT: 10027327) | 21 | 5.60 (3.64-8.63) | 5.60 (77.87) | 2.46 (1.57) | 5.51 (3.58) |
| Postmenopausal haemorrhage (PT: 10055870) | 11 | 7.42 (4.08-13.50) | 7.41 (59.51) | 2.86 (1.41) | 7.25 (3.99) |
| Oligomenorrhoea (PT: 10030295) | 5 | 7.24 (2.98-17.58) | 7.23 (26.20) | 2.82 (0.62) | 7.08 (2.91) |
| **Psychiatric disorders (SOC: 10037175)** | | | | | |
| Sleep disorder due to general medical condition, insomnia type (PT: 10040986) | 24 | 3.36 (2.24-5.02) | 3.36 (39.23) | 1.73 (1.02) | 3.33 (2.23) |
| **Nervous system disorders (SOC: 10029205)** | | | | | |
| Allodynia (PT: 10053552) | 14 | 14.48 (8.47-24.78) | 14.48 (167.23) | 3.79 (2.13) | 13.83 (8.08) |
| Hypoglycaemic unconsciousness (PT: 10065981) | 6 | 12.83 (5.66-29.07) | 12.83 (62.63) | 3.62 (1.11) | 12.32 (5.44) |
| **Endocrine disorders (SOC: 10014698)** | | | | | |
| Thyroid mass (PT: 10058900) | 14 | 4.71 (2.78-7.99) | 4.71 (40.22) | 2.22 (1.15) | 4.65 (2.74) |
| Thyroid cyst (PT: 10043706) | 3 | 9.99 (3.16-31.60) | 9.99 (23.46) | 3.28 (0.13) | 9.69 (3.06) |
| **Eye disorders (SOC: 10015919)** | | | | | |
| Retinopathy (PT: 10038923) | 10 | 5.69 (3.04-10.63) | 5.68 (37.85) | 2.48 (1.10) | 5.59 (2.99) |
| **Blood and lymphatic system disorders (SOC: 10005329)** | | | | | |
| Lymph node pain (PT: 10025182) | 6 | 5.41 (2.41-12.12) | 5.41 (21.14) | 2.41 (0.61) | 5.32 (2.37) |
| **Skin and subcutaneous tissue disorders (SOC: 10040785)** | | | | | |
| Skin sensitisation (PT: 10070835) | 5 | 9.75 (4.00-23.76) | 9.74 (37.95) | 3.24 (0.77) | 9.46 (3.88) |

For tirzepatide, only the reports in 2022-2023 were included. **Abbreviations:** PT, preferred term; ROR, reporting odds ratio; CI, confidence interval; PRR, proportional reporting ratio; χ2, chi-squared; IC, information component; IC025, lower limit of 95% confidence interval of IC; EBGM, empirical Bayesian geometric mean; EBGM05, lower limit of 95% confidence interval of EBGM.

## Supplementary Table S22. Number and signal strength of tirzepatide-related signals at the PT level from the FAERS database based on reports in year 2024.

| **PT** | **Number** | **ROR (95% CI)** | **PRR (χ2)** | **IC (IC025)** | **EBGM (EBGM05)** |
| --- | --- | --- | --- | --- | --- |
| **General disorders and administration site conditions (SOC: 10018065)** | | | | | |
| Injection site pain (PT: 10022086) | 4250 | 17.70 (17.14-18.28) | 16.45 (57284.55) | 3.93 (3.88) | 15.28 (14.79) |
| Injection site haemorrhage (PT: 10022067) | 1709 | 25.86 (24.58-27.22) | 25.12 (35213.40) | 4.49 (4.39) | 22.43 (21.32) |
| Injection site erythema (PT: 10022061) | 1402 | 18.23 (17.25-19.27) | 17.81 (20454.27) | 4.04 (3.94) | 16.43 (15.55) |
| Injection site bruising (PT: 10022052) | 918 | 15.67 (14.65-16.77) | 15.44 (11519.83) | 3.85 (3.73) | 14.40 (13.46) |
| Injection site pruritus (PT: 10022093) | 655 | 14.09 (13.01-15.26) | 13.94 (7360.99) | 3.71 (3.57) | 13.10 (12.09) |
| Injection site mass (PT: 10022081) | 505 | 15.05 (13.74-16.48) | 14.92 (6109.40) | 3.80 (3.63) | 13.96 (12.75) |
| Injection site injury (PT: 10066083) | 431 | 85.38 (76.27-95.59) | 84.75 (25069.41) | 5.90 (5.56) | 59.85 (53.46) |
| Injection site rash (PT: 10022094) | 379 | 14.51 (13.07-16.11) | 14.42 (4417.00) | 3.76 (3.56) | 13.52 (12.17) |
| Injection site swelling (PT: 10053425) | 368 | 5.79 (5.22-6.43) | 5.76 (1410.09) | 2.49 (2.32) | 5.63 (5.07) |
| Injection site urticaria (PT: 10022107) | 304 | 13.84 (12.32-15.55) | 13.77 (3370.34) | 3.69 (3.47) | 12.95 (11.53) |
| Injury associated with device (PT: 10069803) | 270 | 10.56 (9.34-11.94) | 10.52 (2210.74) | 3.33 (3.10) | 10.04 (8.88) |
| Injection site reaction (PT: 10022095) | 247 | 5.59 (4.93-6.35) | 5.57 (902.80) | 2.45 (2.23) | 5.45 (4.80) |
| Injection site discomfort (PT: 10054266) | 123 | 12.27 (10.22-14.72) | 12.24 (1196.99) | 3.54 (3.15) | 11.60 (9.66) |
| Injection site irritation (PT: 10022079) | 120 | 16.86 (13.99-20.31) | 16.83 (1648.01) | 3.96 (3.53) | 15.60 (12.95) |
| Injection site warmth (PT: 10022112) | 89 | 8.85 (7.16-10.95) | 8.84 (592.86) | 3.09 (2.66) | 8.51 (6.88) |
| Injection site coldness (PT: 10050082) | 88 | 156.27 (118.25-206.52) | 156.03 (7620.88) | 6.46 (5.11) | 88.16 (66.71) |
| Injection site hypersensitivity (PT: 10022071) | 77 | 26.77 (21.10-33.96) | 26.74 (1683.19) | 4.57 (3.85) | 23.71 (18.69) |
| Injection site vesicles (PT: 10022111) | 57 | 9.22 (7.07-12.02) | 9.21 (398.88) | 3.15 (2.57) | 8.85 (6.78) |
| Injection site paraesthesia (PT: 10022088) | 48 | 31.87 (23.50-43.22) | 31.85 (1237.45) | 4.79 (3.72) | 27.62 (20.36) |
| Injection site inflammation (PT: 10022078) | 40 | 9.35 (6.81-12.83) | 9.34 (284.67) | 3.16 (2.45) | 8.97 (6.53) |
| Injection site discolouration (PT: 10051572) | 38 | 4.04 (2.93-5.58) | 4.04 (85.27) | 1.99 (1.42) | 3.98 (2.89) |
| Injection site induration (PT: 10022075) | 35 | 4.85 (3.47-6.79) | 4.85 (104.44) | 2.25 (1.62) | 4.76 (3.40) |
| Injection site indentation (PT: 10079277) | 23 | 8.34 (5.49-12.65) | 8.33 (142.51) | 3.01 (2.03) | 8.04 (5.30) |
| Injection site scar (PT: 10059009) | 22 | 8.66 (5.65-13.28) | 8.66 (142.89) | 3.06 (2.04) | 8.34 (5.44) |
| Injection site hypoaesthesia (PT: 10074586) | 13 | 8.77 (5.03-15.29) | 8.77 (85.74) | 3.08 (1.67) | 8.44 (4.85) |
| Injection site laceration (PT: 10067253) | 8 | 22.27 (10.72-46.23) | 22.26 (146.21) | 4.33 (1.67) | 20.14 (9.70) |
| Injection site scab (PT: 10066210) | 7 | 10.16 (4.76-21.72) | 10.16 (55.04) | 3.28 (1.17) | 9.72 (4.55) |
| Thirst decreased (PT: 10050200) | 6 | 11.13 (4.89-25.33) | 11.13 (52.41) | 3.41 (1.03) | 10.60 (4.66) |
| Injection site thrombosis (PT: 10022104) | 5 | 18.55 (7.42-46.38) | 18.55 (75.99) | 4.09 (0.97) | 17.06 (6.83) |
| **Gastrointestinal disorders (SOC: 10017947)** | | | | | |
| Nausea (PT: 10028813) | 2703 | 4.39 (4.22-4.56) | 4.22 (6591.81) | 2.06 (2.00) | 4.16 (4.00) |
| Vomiting (PT: 10047700) | 1053 | 2.86 (2.69-3.04) | 2.83 (1234.35) | 1.49 (1.39) | 2.80 (2.63) |
| Constipation (PT: 10010774) | 837 | 4.29 (4.00-4.60) | 4.24 (2038.03) | 2.06 (1.95) | 4.17 (3.90) |
| Eructation (PT: 10015137) | 443 | 31.82 (28.77-35.18) | 31.58 (11333.74) | 4.78 (4.55) | 27.41 (24.79) |
| Abdominal discomfort (PT: 10000059) | 395 | 2.40 (2.17-2.65) | 2.39 (317.16) | 1.25 (1.10) | 2.38 (2.15) |
| Flatulence (PT: 10016766) | 258 | 5.80 (5.13-6.57) | 5.78 (992.30) | 2.50 (2.29) | 5.65 (4.99) |
| Impaired gastric emptying (PT: 10021518) | 214 | 21.31 (18.51-24.54) | 21.24 (3732.00) | 4.27 (3.95) | 19.30 (16.76) |
| Pancreatitis (PT: 10033645) | 205 | 6.23 (5.42-7.16) | 6.21 (870.38) | 2.60 (2.36) | 6.06 (5.27) |
| Ileus (PT: 10021328) | 32 | 3.82 (2.69-5.42) | 3.82 (65.41) | 1.91 (1.29) | 3.77 (2.66) |
| Food poisoning (PT: 10016952) | 25 | 4.11 (2.76-6.10) | 4.11 (57.56) | 2.02 (1.28) | 4.04 (2.72) |
| Gastrointestinal sounds abnormal (PT: 10067715) | 24 | 4.87 (3.25-7.30) | 4.87 (72.00) | 2.26 (1.47) | 4.78 (3.19) |
| Vomiting projectile (PT: 10047708) | 20 | 9.17 (5.86-14.36) | 9.17 (139.21) | 3.14 (2.04) | 8.81 (5.63) |
| Breath odour (PT: 10006326) | 7 | 4.34 (2.05-9.18) | 4.34 (17.63) | 2.09 (0.56) | 4.27 (2.02) |
| Oesophageal rupture (PT: 10052211) | 5 | 8.35 (3.41-20.43) | 8.35 (31.05) | 3.01 (0.68) | 8.05 (3.29) |
| **Metabolism and nutrition disorders (SOC: 10027433)** | | | | | |
| Dehydration (PT: 10012174) | 241 | 2.53 (2.23-2.87) | 2.52 (219.54) | 1.33 (1.13) | 2.51 (2.21) |
| Feeding disorder (PT: 10061148) | 134 | 5.83 (4.91-6.93) | 5.82 (520.04) | 2.51 (2.21) | 5.68 (4.79) |
| Food craving (PT: 10056465) | 76 | 18.19 (14.38-23.01) | 18.17 (1130.74) | 4.07 (3.45) | 16.74 (13.24) |
| Fluid intake reduced (PT: 10056291) | 27 | 6.16 (4.20-9.03) | 6.15 (113.09) | 2.59 (1.79) | 6.00 (4.09) |
| Food aversion (PT: 10049238) | 12 | 6.62 (3.73-11.77) | 6.62 (55.46) | 2.69 (1.37) | 6.44 (3.63) |
| Starvation ketoacidosis (PT: 10082528) | 4 | 22.26 (7.92-62.55) | 22.26 (73.11) | 4.33 (0.69) | 20.14 (7.17) |
| **Hepatobiliary disorders (SOC: 10019805)** | | | | | |
| Cholelithiasis (PT: 10008629) | 66 | 3.19 (2.50-4.07) | 3.19 (97.50) | 1.66 (1.25) | 3.15 (2.47) |
| Cholecystitis (PT: 10008612) | 34 | 4.45 (3.16-6.25) | 4.44 (88.78) | 2.13 (1.50) | 4.37 (3.11) |
| Gallbladder disorder (PT: 10017626) | 33 | 3.18 (2.25-4.48) | 3.17 (48.39) | 1.65 (1.06) | 3.14 (2.23) |
| Cholecystitis acute (PT: 10008614) | 13 | 3.67 (2.12-6.35) | 3.67 (24.78) | 1.86 (0.83) | 3.62 (2.09) |
| **Investigations (SOC: 10022891)** | | | | | |
| Lipase increased (PT: 10024574) | 44 | 9.01 (6.66-12.19) | 9.00 (299.67) | 3.11 (2.45) | 8.66 (6.40) |
| Amylase increased (PT: 10002016) | 15 | 4.70 (2.81-7.84) | 4.70 (42.64) | 2.21 (1.18) | 4.61 (2.76) |
| Pancreatic enzymes increased (PT: 10061900) | 8 | 7.12 (3.52-14.42) | 7.12 (40.67) | 2.79 (1.08) | 6.91 (3.42) |
| Gastric pH decreased (PT: 10060041) | 8 | 9.77 (4.81-19.88) | 9.77 (60.08) | 3.23 (1.29) | 9.37 (4.61) |
| Blood folate decreased (PT: 10005527) | 5 | 7.16 (2.93-17.46) | 7.16 (25.56) | 2.80 (0.60) | 6.94 (2.85) |
| **Skin and subcutaneous tissue disorders (SOC: 10040785)** | | | | | |
| Sensitive skin (PT: 10081765) | 40 | 4.21 (3.08-5.76) | 4.21 (95.88) | 2.05 (1.49) | 4.14 (3.03) |
| Skin laxity (PT: 10057064) | 6 | 10.73 (4.72-24.41) | 10.73 (50.27) | 3.36 (1.02) | 10.24 (4.50) |
| Skin sensitisation (PT: 10070835) | 5 | 6.82 (2.80-16.62) | 6.81 (23.99) | 2.73 (0.57) | 6.62 (2.72) |
| **Psychiatric disorders (SOC: 10037175)** | | | | | |
| Sleep disorder due to general medical condition, insomnia type (PT: 10040986) | 49 | 4.85 (3.66-6.44) | 4.85 (146.27) | 2.25 (1.73) | 4.76 (3.58) |
| **Reproductive system and breast disorders (SOC: 10038604)** | | | | | |
| Postmenopausal haemorrhage (PT: 10055870) | 18 | 8.63 (5.38-13.83) | 8.63 (116.38) | 3.06 (1.91) | 8.31 (5.19) |
| Oligomenorrhoea (PT: 10030295) | 6 | 6.10 (2.71-13.75) | 6.10 (24.84) | 2.57 (0.69) | 5.95 (2.64) |
| **Nervous system disorders (SOC: 10029205)** | | | | | |
| Allodynia (PT: 10053552) | 13 | 9.37 (5.37-16.34) | 9.37 (92.85) | 3.17 (1.72) | 9.00 (5.16) |
| Hypoglycaemic unconsciousness (PT: 10065981) | 5 | 7.42 (3.04-18.12) | 7.42 (26.79) | 2.85 (0.62) | 7.19 (2.95) |
| Wernicke's encephalopathy (PT: 10047911) | 5 | 11.13 (4.52-27.40) | 11.13 (43.68) | 3.41 (0.81) | 10.60 (4.31) |
| **Infections and infestations (SOC: 10021881)** | | | | | |
| Injection site infection (PT: 10022076) | 16 | 7.37 (4.48-12.14) | 7.37 (84.96) | 2.84 (1.68) | 7.14 (4.34) |
| Injection site pustule (PT: 10054994) | 5 | 14.11 (5.70-34.95) | 14.11 (56.90) | 3.73 (0.89) | 13.25 (5.35) |
| **Endocrine disorders (SOC: 10014698)** | | | | | |
| Thyroid mass (PT: 10058900) | 18 | 4.25 (2.67-6.79) | 4.25 (43.86) | 2.07 (1.17) | 4.19 (2.62) |
| **Immune system disorders (SOC: 10021428)** | | | | | |
| Allergy to metals (PT: 10066414) | 4 | 7.29 (2.69-19.76) | 7.29 (20.93) | 2.82 (0.35) | 7.07 (2.61) |

For tirzepatide, only the reports in 2024 were included. **Abbreviations:** PT, preferred term; ROR, reporting odds ratio; CI, confidence interval; PRR, proportional reporting ratio; χ2, chi-squared; IC, information component; IC025, lower limit of 95% confidence interval of IC; EBGM, empirical Bayesian geometric mean; EBGM05, lower limit of 95% confidence interval of EBGM.

## Supplementary Table S23. Number and signal strength of tirzepatide-related signals at the PT level from the FAERS database based on reports occur on the day of medication.

| **PT** | **Number** | **ROR (95% CI)** | **PRR (χ2)** | **IC (IC025)** | **EBGM (EBGM05)** |
| --- | --- | --- | --- | --- | --- |
| **General disorders and administration site conditions (SOC: 10018065)** | | | | | |
| Injection site pain (PT: 10022086) | 260 | 9.90 (8.74-11.22) | 9.49 (1975.28) | 3.24 (3.01) | 9.45 (8.34) |
| Injection site haemorrhage (PT: 10022067) | 127 | 17.35 (14.54-20.70) | 16.98 (1897.08) | 4.07 (3.65) | 16.85 (14.12) |
| Injection site erythema (PT: 10022061) | 95 | 11.49 (9.38-14.08) | 11.31 (889.70) | 3.49 (3.05) | 11.26 (9.19) |
| Injection site bruising (PT: 10022052) | 76 | 12.27 (9.78-15.40) | 12.12 (771.70) | 3.59 (3.07) | 12.05 (9.61) |
| Injection site pruritus (PT: 10022093) | 42 | 8.58 (6.33-11.63) | 8.52 (277.89) | 3.09 (2.41) | 8.49 (6.26) |
| Injury associated with device (PT: 10069803) | 37 | 14.09 (10.19-19.49) | 14.00 (443.92) | 3.80 (2.90) | 13.91 (10.06) |
| Injection site mass (PT: 10022081) | 35 | 9.88 (7.08-13.78) | 9.82 (276.19) | 3.29 (2.49) | 9.78 (7.01) |
| Injection site swelling (PT: 10053425) | 26 | 4.04 (2.75-5.94) | 4.03 (59.13) | 2.01 (1.30) | 4.02 (2.73) |
| Injection site rash (PT: 10022094) | 24 | 8.72 (5.83-13.03) | 8.69 (162.61) | 3.11 (2.15) | 8.65 (5.79) |
| Injection site injury (PT: 10066083) | 22 | 31.47 (20.64-47.98) | 31.35 (636.59) | 4.95 (3.14) | 30.89 (20.26) |
| Injection site urticaria (PT: 10022107) | 19 | 8.24 (5.24-12.93) | 8.21 (119.89) | 3.03 (1.94) | 8.18 (5.21) |
| Injection site reaction (PT: 10022095) | 18 | 4.03 (2.54-6.41) | 4.02 (40.81) | 2.01 (1.13) | 4.02 (2.53) |
| Injection site warmth (PT: 10022112) | 14 | 13.65 (8.06-23.10) | 13.62 (162.60) | 3.76 (2.13) | 13.53 (8.00) |
| Feeling cold (PT: 10016326) | 10 | 4.41 (2.37-8.21) | 4.40 (26.26) | 2.14 (0.88) | 4.40 (2.36) |
| Thirst (PT: 10043458) | 8 | 4.16 (2.08-8.33) | 4.16 (19.15) | 2.05 (0.66) | 4.15 (2.07) |
| Injection site irritation (PT: 10022079) | 5 | 6.59 (2.74-15.87) | 6.59 (23.63) | 2.72 (0.59) | 6.57 (2.73) |
| Tenderness (PT: 10043224) | 5 | 5.60 (2.33-13.48) | 5.60 (18.84) | 2.48 (0.48) | 5.59 (2.32) |
| Injection site paraesthesia (PT: 10022088) | 3 | 17.60 (5.65-54.85) | 17.59 (46.54) | 4.13 (0.32) | 17.45 (5.60) |
| Injection site indentation (PT: 10079277) | 3 | 10.66 (3.43-33.15) | 10.65 (26.10) | 3.41 (0.19) | 10.60 (3.41) |
| Injection site hypersensitivity (PT: 10022071) | 3 | 9.38 (3.02-29.17) | 9.38 (22.35) | 3.22 (0.15) | 9.34 (3.00) |
| **Gastrointestinal disorders (SOC: 10017947)** | | | | | |
| Nausea (PT: 10028813) | 338 | 5.54 (4.96-6.19) | 5.27 (1179.15) | 2.39 (2.21) | 5.26 (4.71) |
| Vomiting (PT: 10047700) | 143 | 3.93 (3.33-4.64) | 3.85 (303.38) | 1.94 (1.67) | 3.85 (3.26) |
| Constipation (PT: 10010774) | 76 | 3.88 (3.09-4.86) | 3.84 (159.72) | 1.94 (1.55) | 3.83 (3.06) |
| Eructation (PT: 10015137) | 47 | 30.06 (22.51-40.14) | 29.82 (1290.52) | 4.88 (3.79) | 29.40 (22.02) |
| Flatulence (PT: 10016766) | 25 | 5.56 (3.75-8.25) | 5.54 (92.93) | 2.47 (1.67) | 5.53 (3.73) |
| Retching (PT: 10038776) | 9 | 5.50 (2.86-10.58) | 5.49 (32.99) | 2.45 (1.01) | 5.48 (2.85) |
| Paraesthesia oral (PT: 10057372) | 6 | 4.85 (2.18-10.81) | 4.85 (18.28) | 2.27 (0.55) | 4.84 (2.17) |
| **Metabolism and nutrition disorders (SOC: 10027433)** | | | | | |
| Feeding disorder (PT: 10061148) | 30 | 13.00 (9.07-18.64) | 12.94 (328.58) | 3.69 (2.70) | 12.86 (8.98) |
| Hypophagia (PT: 10063743) | 10 | 4.78 (2.57-8.90) | 4.78 (29.80) | 2.25 (0.96) | 4.77 (2.56) |
| Fluid intake reduced (PT: 10056291) | 5 | 11.30 (4.69-27.23) | 11.29 (46.64) | 3.49 (0.87) | 11.23 (4.66) |
| Food craving (PT: 10056465) | 3 | 6.70 (2.16-20.83) | 6.70 (14.50) | 2.74 (0.02) | 6.68 (2.15) |
| **Musculoskeletal and connective tissue disorders (SOC: 10028395)** | | | | | |
| Neck pain (PT: 10028836) | 18 | 3.69 (2.32-5.86) | 3.68 (35.12) | 1.88 (1.02) | 3.68 (2.31) |
| Neck mass (PT: 10049146) | 3 | 12.62 (4.05-39.27) | 12.61 (31.88) | 3.65 (0.24) | 12.54 (4.03) |
| **Psychiatric disorders (SOC: 10037175)** | | | | | |
| Panic attack (PT: 10033664) | 10 | 3.86 (2.07-7.18) | 3.85 (21.08) | 1.94 (0.74) | 3.85 (2.07) |
| Sleep disorder due to general medical condition, insomnia type (PT: 10040986) | 9 | 8.88 (4.61-17.11) | 8.87 (62.60) | 3.14 (1.39) | 8.84 (4.59) |
| **Skin and subcutaneous tissue disorders (SOC: 10040785)** | | | | | |
| Pain of skin (PT: 10033474) | 11 | 5.64 (3.12-10.20) | 5.63 (41.79) | 2.49 (1.18) | 5.62 (3.11) |
| Sensitive skin (PT: 10081765) | 8 | 8.42 (4.20-16.87) | 8.41 (52.01) | 3.07 (1.24) | 8.38 (4.18) |
| **Immune system disorders (SOC: 10021428)** | | | | | |
| Anaphylactic reaction (PT: 10002198) | 17 | 3.39 (2.10-5.46) | 3.38 (28.48) | 1.76 (0.89) | 3.38 (2.10) |
| **Blood and lymphatic system disorders (SOC: 10005329)** | | | | | |
| Lymphadenopathy (PT: 10025197) | 11 | 3.70 (2.05-6.69) | 3.69 (21.58) | 1.88 (0.76) | 3.69 (2.04) |
| Lymph node pain (PT: 10025182) | 3 | 19.03 (6.10-59.32) | 19.02 (50.73) | 4.24 (0.33) | 18.85 (6.05) |
| **Nervous system disorders (SOC: 10029205)** | | | | | |
| Hyperaesthesia (PT: 10020568) | 6 | 10.13 (4.54-22.61) | 10.12 (49.09) | 3.33 (1.04) | 10.08 (4.52) |
| Allodynia (PT: 10053552) | 5 | 35.60 (14.70-86.22) | 35.57 (165.11) | 5.13 (1.20) | 34.98 (14.44) |
| **Investigations (SOC: 10022891)** | | | | | |
| Gastric pH decreased (PT: 10060041) | 4 | 48.48 (17.98-130.72) | 48.44 (181.54) | 5.57 (0.89) | 47.34 (17.56) |
| **Reproductive system and breast disorders (SOC: 10038604)** | | | | | |
| Breast tenderness (PT: 10006313) | 3 | 10.83 (3.48-33.68) | 10.82 (26.60) | 3.43 (0.20) | 10.77 (3.46) |

For tirzepatide, only the reports occur on the day of medication were included. **Abbreviations:** PT, preferred term; ROR, reporting odds ratio; CI, confidence interval; PRR, proportional reporting ratio; χ2, chi-squared; IC, information component; IC025, lower limit of 95% confidence interval of IC; EBGM, empirical Bayesian geometric mean; EBGM05, lower limit of 95% confidence interval of EBGM.

## Supplementary Table S24. Number and signal strength of tirzepatide-related signals at the PT level from the FAERS database based on reports occur on 1-30 days of medication.

| **PT** | **Number** | **ROR (95% CI)** | **PRR (χ2)** | **IC (IC025)** | **EBGM (EBGM05)** |
| --- | --- | --- | --- | --- | --- |
| **Gastrointestinal disorders (SOC: 10017947)** | | | | | |
| Nausea (PT: 10028813) | 191 | 4.91 (4.25-5.68) | 4.70 (562.33) | 2.23 (1.99) | 4.70 (4.06) |
| Diarrhoea (PT: 10012735) | 162 | 4.43 (3.78-5.19) | 4.27 (410.11) | 2.09 (1.83) | 4.27 (3.65) |
| Vomiting (PT: 10047700) | 120 | 5.25 (4.38-6.30) | 5.11 (398.69) | 2.35 (2.04) | 5.10 (4.25) |
| Constipation (PT: 10010774) | 56 | 4.52 (3.47-5.89) | 4.47 (151.13) | 2.16 (1.69) | 4.46 (3.43) |
| Abdominal pain (PT: 10000081) | 42 | 3.47 (2.56-4.71) | 3.44 (72.99) | 1.78 (1.26) | 3.44 (2.54) |
| Eructation (PT: 10015137) | 38 | 38.42 (27.85-52.99) | 38.02 (1354.06) | 5.23 (3.81) | 37.59 (27.25) |
| Flatulence (PT: 10016766) | 27 | 9.54 (6.53-13.93) | 9.47 (204.12) | 3.24 (2.31) | 9.45 (6.46) |
| Gastrointestinal pain (PT: 10017999) | 6 | 9.15 (4.10-20.39) | 9.13 (43.33) | 3.19 (0.98) | 9.11 (4.08) |
| Impaired gastric emptying (PT: 10021518) | 5 | 7.23 (3.00-17.40) | 7.22 (26.74) | 2.85 (0.64) | 7.21 (2.99) |
| Small intestinal obstruction (PT: 10041101) | 4 | 6.94 (2.60-18.52) | 6.93 (20.27) | 2.79 (0.37) | 6.92 (2.59) |
| Vomiting projectile (PT: 10047708) | 4 | 28.44 (10.62-76.15) | 28.41 (104.86) | 4.82 (0.83) | 28.17 (10.52) |
| **General disorders and administration site conditions (SOC: 10018065)** | | | | | |
| Injection site pain (PT: 10022086) | 74 | 4.33 (3.44-5.45) | 4.26 (185.06) | 2.09 (1.69) | 4.25 (3.38) |
| Injection site erythema (PT: 10022061) | 49 | 9.32 (7.03-12.36) | 9.20 (357.84) | 3.20 (2.57) | 9.18 (6.92) |
| Injection site haemorrhage (PT: 10022067) | 46 | 9.79 (7.32-13.10) | 9.68 (357.26) | 3.27 (2.60) | 9.65 (7.21) |
| Injection site bruising (PT: 10022052) | 32 | 8.11 (5.72-11.49) | 8.04 (197.07) | 3.00 (2.22) | 8.02 (5.66) |
| Injection site pruritus (PT: 10022093) | 24 | 7.73 (5.17-11.56) | 7.69 (139.37) | 2.94 (2.02) | 7.67 (5.13) |
| Injection site swelling (PT: 10053425) | 20 | 4.92 (3.17-7.64) | 4.90 (62.01) | 2.29 (1.41) | 4.89 (3.15) |
| Injection site rash (PT: 10022094) | 17 | 9.76 (6.06-15.73) | 9.72 (132.60) | 3.28 (2.02) | 9.69 (6.01) |
| Injection site mass (PT: 10022081) | 13 | 5.77 (3.34-9.95) | 5.75 (50.98) | 2.52 (1.33) | 5.74 (3.33) |
| Injury associated with device (PT: 10069803) | 13 | 7.77 (4.50-13.40) | 7.75 (76.23) | 2.95 (1.61) | 7.73 (4.48) |
| Injection site injury (PT: 10066083) | 13 | 29.21 (16.90-50.48) | 29.11 (349.72) | 4.85 (2.49) | 28.86 (16.70) |
| Injection site urticaria (PT: 10022107) | 11 | 7.52 (4.16-13.61) | 7.50 (61.90) | 2.90 (1.45) | 7.49 (4.14) |
| Injection site warmth (PT: 10022112) | 7 | 10.75 (5.11-22.59) | 10.73 (61.57) | 3.42 (1.25) | 10.70 (5.09) |
| Facial pain (PT: 10016059) | 5 | 9.53 (3.96-22.93) | 9.51 (37.99) | 3.25 (0.79) | 9.49 (3.94) |
| Injection site hypersensitivity (PT: 10022071) | 3 | 14.84 (4.77-46.15) | 14.83 (38.51) | 3.88 (0.28) | 14.76 (4.75) |
| **Metabolism and nutrition disorders (SOC: 10027433)** | | | | | |
| Dehydration (PT: 10012174) | 48 | 8.09 (6.08-10.76) | 7.99 (293.51) | 3.00 (2.39) | 7.98 (6.00) |
| Hypophagia (PT: 10063743) | 11 | 8.33 (4.61-15.07) | 8.31 (70.58) | 3.05 (1.53) | 8.29 (4.58) |
| Feeding disorder (PT: 10061148) | 9 | 6.12 (3.18-11.79) | 6.11 (38.42) | 2.61 (1.10) | 6.10 (3.17) |
| Diabetic ketoacidosis (PT: 10012671) | 6 | 5.79 (2.60-12.91) | 5.79 (23.71) | 2.53 (0.69) | 5.78 (2.59) |
| Electrolyte imbalance (PT: 10014418) | 4 | 6.22 (2.33-16.61) | 6.22 (17.48) | 2.63 (0.31) | 6.21 (2.33) |
| Food craving (PT: 10056465) | 3 | 10.60 (3.41-32.95) | 10.60 (25.99) | 3.40 (0.19) | 10.56 (3.40) |
| Fluid intake reduced (PT: 10056291) | 3 | 10.70 (3.44-33.25) | 10.69 (26.26) | 3.41 (0.20) | 10.66 (3.43) |
| **Investigations (SOC: 10022891)** | | | | | |
| Heart rate increased (PT: 10019303) | 20 | 3.63 (2.34-5.64) | 3.62 (37.92) | 1.85 (1.05) | 3.62 (2.33) |
| Lipase increased (PT: 10024574) | 3 | 9.47 (3.05-29.43) | 9.46 (22.64) | 3.24 (0.16) | 9.44 (3.04) |
| **Reproductive system and breast disorders (SOC: 10038604)** | | | | | |
| Vaginal haemorrhage (PT: 10046910) | 8 | 6.77 (3.38-13.57) | 6.76 (39.20) | 2.75 (1.08) | 6.75 (3.37) |
| Heavy menstrual bleeding (PT: 10085423) | 5 | 4.91 (2.04-11.82) | 4.91 (15.53) | 2.29 (0.39) | 4.90 (2.04) |
| Dysmenorrhoea (PT: 10013935) | 4 | 8.21 (3.07-21.90) | 8.20 (25.22) | 3.03 (0.45) | 8.18 (3.06) |
| Menstruation delayed (PT: 10027336) | 4 | 13.48 (5.04-36.00) | 13.46 (45.96) | 3.75 (0.65) | 13.41 (5.02) |
| **Skin and subcutaneous tissue disorders (SOC: 10040785)** | | | | | |
| Sensitive skin (PT: 10081765) | 8 | 13.32 (6.65-26.70) | 13.30 (90.61) | 3.73 (1.52) | 13.24 (6.61) |
| Pain of skin (PT: 10033474) | 7 | 5.67 (2.70-11.91) | 5.66 (26.82) | 2.50 (0.81) | 5.65 (2.69) |
| Cold sweat (PT: 10009866) | 5 | 6.52 (2.71-15.69) | 6.51 (23.29) | 2.70 (0.58) | 6.50 (2.70) |
| **Musculoskeletal and connective tissue disorders (SOC: 10028395)** | | | | | |
| Neck pain (PT: 10028836) | 15 | 4.87 (2.93-8.08) | 4.85 (45.81) | 2.28 (1.24) | 4.84 (2.92) |
| **Psychiatric disorders (SOC: 10037175)** | | | | | |
| Panic attack (PT: 10033664) | 9 | 5.49 (2.85-10.57) | 5.48 (32.92) | 2.45 (1.00) | 5.47 (2.84) |
| Sleep disorder due to general medical condition, insomnia type (PT: 10040986) | 5 | 7.79 (3.24-18.74) | 7.78 (29.47) | 2.96 (0.69) | 7.76 (3.23) |
| **Hepatobiliary disorders (SOC: 10019805)** | | | | | |
| Cholelithiasis (PT: 10008629) | 7 | 5.36 (2.55-11.26) | 5.35 (24.73) | 2.42 (0.77) | 5.34 (2.54) |
| Gallbladder disorder (PT: 10017626) | 5 | 7.63 (3.17-18.36) | 7.62 (28.70) | 2.93 (0.67) | 7.61 (3.16) |
| **Nervous system disorders (SOC: 10029205)** | | | | | |
| Allodynia (PT: 10053552) | 4 | 44.89 (16.73-120.50) | 44.84 (169.11) | 5.47 (0.89) | 44.24 (16.48) |
| Hyperaesthesia (PT: 10020568) | 3 | 7.99 (2.57-24.82) | 7.98 (18.28) | 2.99 (0.09) | 7.97 (2.56) |
| **Blood and lymphatic system disorders (SOC: 10005329)** | | | | | |
| Lymph node pain (PT: 10025182) | 3 | 30.09 (9.65-93.85) | 30.07 (83.53) | 4.90 (0.41) | 29.80 (9.56) |

For tirzepatide, only the reports occur on 1-30 days of medication were included. **Abbreviations:** PT, preferred term; ROR, reporting odds ratio; CI, confidence interval; PRR, proportional reporting ratio; χ2, chi-squared; IC, information component; IC025, lower limit of 95% confidence interval of IC; EBGM, empirical Bayesian geometric mean; EBGM05, lower limit of 95% confidence interval of EBGM.

## Supplementary Table S25. Number and signal strength of tirzepatide-related signals at the PT level from the FAERS database based on reports occur over 30 days of medication.

| **PT** | **Number** | **ROR (95% CI)** | **PRR (χ2)** | **IC (IC025)** | **EBGM (EBGM05)** |
| --- | --- | --- | --- | --- | --- |
| **General disorders and administration site conditions (SOC: 10018065)** | | | | | |
| Injection site pain (PT: 10022086) | 110 | 7.54 (6.24-9.13) | 7.31 (600.91) | 2.87 (2.51) | 7.30 (6.03) |
| Injection site erythema (PT: 10022061) | 62 | 13.71 (10.66-17.64) | 13.45 (713.30) | 3.75 (3.12) | 13.41 (10.42) |
| Injection site haemorrhage (PT: 10022067) | 57 | 14.09 (10.84-18.32) | 13.85 (678.04) | 3.79 (3.12) | 13.80 (10.62) |
| Injection site pruritus (PT: 10022093) | 51 | 19.21 (14.55-25.35) | 18.91 (861.27) | 4.23 (3.40) | 18.82 (14.26) |
| Injection site bruising (PT: 10022052) | 42 | 12.35 (9.10-16.75) | 12.20 (430.73) | 3.60 (2.83) | 12.16 (8.96) |
| Injection site swelling (PT: 10053425) | 27 | 7.70 (5.27-11.24) | 7.64 (155.58) | 2.93 (2.08) | 7.62 (5.22) |
| Injection site rash (PT: 10022094) | 25 | 16.65 (11.22-24.70) | 16.52 (363.08) | 4.04 (2.80) | 16.45 (11.09) |
| Injection site urticaria (PT: 10022107) | 20 | 15.88 (10.22-24.67) | 15.78 (275.86) | 3.97 (2.57) | 15.72 (10.12) |
| Injection site injury (PT: 10066083) | 17 | 44.30 (27.43-71.56) | 44.06 (707.19) | 5.44 (3.01) | 43.56 (26.97) |
| Injection site mass (PT: 10022081) | 15 | 7.70 (4.63-12.79) | 7.66 (86.79) | 2.94 (1.71) | 7.65 (4.60) |
| Injection site reaction (PT: 10022095) | 11 | 4.50 (2.49-8.13) | 4.48 (29.76) | 2.16 (0.96) | 4.48 (2.48) |
| Injection site warmth (PT: 10022112) | 8 | 14.20 (7.08-28.45) | 14.16 (97.52) | 3.82 (1.56) | 14.11 (7.04) |
| Injury associated with device (PT: 10069803) | 7 | 4.82 (2.29-10.12) | 4.81 (21.11) | 2.26 (0.68) | 4.80 (2.29) |
| Injection site discomfort (PT: 10054266) | 5 | 8.73 (3.63-21.01) | 8.72 (34.08) | 3.12 (0.75) | 8.70 (3.61) |
| Injection site coldness (PT: 10050082) | 4 | 75.52 (28.05-203.34) | 75.42 (287.89) | 6.21 (0.94) | 73.94 (27.46) |
| Injection site irritation (PT: 10022079) | 3 | 7.21 (2.32-22.40) | 7.21 (16.01) | 2.85 (0.05) | 7.20 (2.32) |
| Injection site hypersensitivity (PT: 10022071) | 3 | 17.13 (5.51-53.29) | 17.12 (45.32) | 4.09 (0.32) | 17.04 (5.48) |
| **Gastrointestinal disorders (SOC: 10017947)** | | | | | |
| Nausea (PT: 10028813) | 99 | 2.87 (2.35-3.51) | 2.81 (116.81) | 1.49 (1.17) | 2.81 (2.30) |
| Vomiting (PT: 10047700) | 64 | 3.19 (2.49-4.09) | 3.14 (94.11) | 1.65 (1.24) | 3.14 (2.45) |
| Constipation (PT: 10010774) | 38 | 3.53 (2.56-4.86) | 3.50 (68.02) | 1.81 (1.25) | 3.50 (2.54) |
| Pancreatitis (PT: 10033645) | 28 | 15.46 (10.64-22.44) | 15.32 (373.58) | 3.93 (2.82) | 15.27 (10.51) |
| Eructation (PT: 10015137) | 19 | 21.94 (13.96-34.50) | 21.82 (375.27) | 4.44 (2.76) | 21.69 (13.80) |
| Flatulence (PT: 10016766) | 17 | 6.91 (4.29-11.13) | 6.88 (85.28) | 2.78 (1.69) | 6.87 (4.26) |
| Impaired gastric emptying (PT: 10021518) | 12 | 20.14 (11.41-35.56) | 20.07 (216.30) | 4.32 (2.22) | 19.97 (11.31) |
| Pancreatitis acute (PT: 10033647) | 9 | 10.23 (5.31-19.69) | 10.20 (74.51) | 3.35 (1.49) | 10.18 (5.28) |
| Small intestinal obstruction (PT: 10041101) | 4 | 8.01 (3.00-21.39) | 8.01 (24.47) | 3.00 (0.44) | 7.99 (2.99) |
| Ileus paralytic (PT: 10021333) | 3 | 18.31 (5.89-56.98) | 18.30 (48.82) | 4.19 (0.33) | 18.21 (5.85) |
| Oesophageal rupture (PT: 10052211) | 3 | 91.43 (29.07-287.58) | 91.34 (261.62) | 6.48 (0.49) | 89.17 (28.35) |
| Food poisoning (PT: 10016952) | 3 | 8.98 (2.89-27.90) | 8.97 (21.20) | 3.16 (0.14) | 8.95 (2.88) |
| Pancreatic cyst (PT: 10033615) | 3 | 38.46 (12.33-120.02) | 38.42 (108.24) | 5.25 (0.44) | 38.04 (12.19) |
| Gastrointestinal necrosis (PT: 10017982) | 3 | 30.73 (9.86-95.77) | 30.70 (85.49) | 4.93 (0.41) | 30.45 (9.77) |
| **Psychiatric disorders (SOC: 10037175)** | | | | | |
| Anxiety (PT: 10002855) | 36 | 3.00 (2.16-4.16) | 2.97 (47.30) | 1.57 (1.02) | 2.97 (2.14) |
| Suicidal ideation (PT: 10042458) | 22 | 6.59 (4.33-10.03) | 6.55 (103.37) | 2.71 (1.79) | 6.54 (4.30) |
| Panic attack (PT: 10033664) | 16 | 11.32 (6.92-18.51) | 11.26 (149.23) | 3.49 (2.11) | 11.23 (6.87) |
| **Metabolism and nutrition disorders (SOC: 10027433)** | | | | | |
| Dehydration (PT: 10012174) | 27 | 5.22 (3.57-7.63) | 5.19 (91.25) | 2.37 (1.62) | 5.18 (3.55) |
| Malnutrition (PT: 10061273) | 7 | 14.59 (6.94-30.67) | 14.56 (88.06) | 3.86 (1.41) | 14.51 (6.90) |
| Food craving (PT: 10056465) | 3 | 12.24 (3.94-38.05) | 12.23 (30.84) | 3.61 (0.24) | 12.20 (3.92) |
| Fluid intake reduced (PT: 10056291) | 3 | 12.35 (3.97-38.39) | 12.34 (31.16) | 3.62 (0.24) | 12.30 (3.96) |
| **Hepatobiliary disorders (SOC: 10019805)** | | | | | |
| Cholecystitis (PT: 10008612) | 7 | 16.70 (7.94-35.12) | 16.67 (102.64) | 4.05 (1.47) | 16.60 (7.89) |
| Cholelithiasis (PT: 10008629) | 7 | 6.19 (2.95-13.00) | 6.18 (30.34) | 2.63 (0.88) | 6.17 (2.94) |
| Cholecystitis acute (PT: 10008614) | 5 | 25.91 (10.74-62.48) | 25.87 (118.71) | 4.68 (1.14) | 25.69 (10.65) |
| Cholangitis acute (PT: 10008605) | 3 | 78.55 (25.02-246.60) | 78.47 (224.71) | 6.26 (0.48) | 76.87 (24.49) |
| **Investigations (SOC: 10022891)** | | | | | |
| Lipase increased (PT: 10024574) | 9 | 33.06 (17.14-63.79) | 32.97 (276.56) | 5.03 (2.05) | 32.69 (16.94) |
| Quality of life decreased (PT: 10067620) | 4 | 6.34 (2.38-16.91) | 6.33 (17.93) | 2.66 (0.32) | 6.32 (2.37) |
| **Infections and infestations (SOC: 10021881)** | | | | | |
| Gastroenteritis viral (PT: 10017918) | 6 | 6.02 (2.70-13.43) | 6.01 (25.05) | 2.59 (0.71) | 6.01 (2.69) |
| Appendicitis (PT: 10003011) | 4 | 8.13 (3.05-21.71) | 8.12 (24.94) | 3.02 (0.45) | 8.11 (3.04) |
| **Musculoskeletal and connective tissue disorders (SOC: 10028395)** | | | | | |
| Muscle atrophy (PT: 10028289) | 4 | 6.60 (2.48-17.62) | 6.60 (18.97) | 2.72 (0.34) | 6.59 (2.47) |
| **Respiratory, thoracic and mediastinal disorders (SOC: 10038738)** | | | | | |
| Pneumomediastinum (PT: 10050184) | 4 | 37.19 (13.88-99.64) | 37.14 (139.30) | 5.20 (0.87) | 36.79 (13.73) |
| **Renal and urinary disorders (SOC: 10038359)** | | | | | |
| Renal cyst (PT: 10038423) | 3 | 9.24 (2.97-28.71) | 9.23 (21.97) | 3.20 (0.15) | 9.21 (2.97) |

For tirzepatide, only the reports occur over 30 days of medication were included. **Abbreviations:** PT, preferred term; ROR, reporting odds ratio; CI, confidence interval; PRR, proportional reporting ratio; χ2, chi-squared; IC, information component; IC025, lower limit of 95% confidence interval of IC; EBGM, empirical Bayesian geometric mean; EBGM05, lower limit of 95% confidence interval of EBGM.

## Supplementary Table S26. Number and signal strength of tirzepatide-related signals at the PT level from the FAERS database based on reports with serious outcomes.

| **PT** | **Number** | **ROR (95% CI)** | **PRR (χ2)** | **IC (IC025)** | **EBGM (EBGM05)** |
| --- | --- | --- | --- | --- | --- |
| **Gastrointestinal disorders (SOC: 10017947)** | | | | | |
| Vomiting (PT: 10047700) | 532 | 6.58 (6.03-7.18) | 6.35 (2396.55) | 2.66 (2.52) | 6.31 (5.79) |
| Nausea (PT: 10028813) | 471 | 3.32 (3.03-3.65) | 3.24 (734.73) | 1.69 (1.55) | 3.23 (2.95) |
| Diarrhoea (PT: 10012735) | 427 | 3.22 (2.92-3.55) | 3.15 (629.93) | 1.65 (1.50) | 3.14 (2.85) |
| Pancreatitis (PT: 10033645) | 424 | 61.51 (55.66-67.97) | 59.50 (22883.16) | 5.80 (5.48) | 55.86 (50.55) |
| Abdominal pain (PT: 10000081) | 216 | 5.03 (4.39-5.75) | 4.96 (681.09) | 2.30 (2.08) | 4.94 (4.31) |
| Impaired gastric emptying (PT: 10021518) | 194 | 86.63 (74.69-100.48) | 85.33 (14766.38) | 6.29 (5.59) | 78.00 (67.25) |
| Constipation (PT: 10010774) | 189 | 4.27 (3.69-4.93) | 4.22 (463.54) | 2.07 (1.84) | 4.20 (3.64) |
| Intestinal obstruction (PT: 10022687) | 101 | 13.61 (11.17-16.57) | 13.51 (1153.05) | 3.74 (3.28) | 13.32 (10.94) |
| Pancreatitis acute (PT: 10033647) | 59 | 16.47 (12.72-21.32) | 16.40 (837.95) | 4.01 (3.31) | 16.12 (12.45) |
| Eructation (PT: 10015137) | 47 | 13.19 (9.88-17.60) | 13.14 (519.85) | 3.70 (2.95) | 12.97 (9.72) |
| Small intestinal obstruction (PT: 10041101) | 43 | 21.29 (15.73-28.83) | 21.22 (809.63) | 4.38 (3.40) | 20.76 (15.33) |
| Ileus (PT: 10021328) | 38 | 20.44 (14.81-28.21) | 20.38 (684.94) | 4.32 (3.28) | 19.95 (14.46) |
| Flatulence (PT: 10016766) | 37 | 3.63 (2.63-5.01) | 3.62 (69.98) | 1.85 (1.29) | 3.61 (2.61) |
| Gastritis (PT: 10017853) | 25 | 5.86 (3.96-8.69) | 5.85 (100.00) | 2.54 (1.73) | 5.82 (3.93) |
| Vomiting projectile (PT: 10047708) | 14 | 28.37 (16.66-48.32) | 28.34 (357.99) | 4.78 (2.55) | 27.50 (16.15) |
| Haematemesis (PT: 10018830) | 13 | 3.51 (2.04-6.06) | 3.51 (23.26) | 1.81 (0.80) | 3.50 (2.03) |
| Colitis ischaemic (PT: 10009895) | 11 | 11.47 (6.32-20.79) | 11.46 (103.68) | 3.50 (1.77) | 11.33 (6.25) |
| Pancreatitis necrotising (PT: 10033654) | 11 | 22.70 (12.47-41.30) | 22.68 (222.34) | 4.47 (2.16) | 22.14 (12.17) |
| Food poisoning (PT: 10016952) | 11 | 8.00 (4.42-14.49) | 7.99 (66.73) | 2.99 (1.49) | 7.93 (4.38) |
| Ileus paralytic (PT: 10021333) | 10 | 14.91 (7.98-27.86) | 14.90 (127.54) | 3.87 (1.83) | 14.67 (7.85) |
| Faecaloma (PT: 10056325) | 9 | 9.74 (5.05-18.80) | 9.74 (69.80) | 3.27 (1.45) | 9.64 (5.00) |
| Gastrointestinal necrosis (PT: 10017982) | 7 | 17.50 (8.28-36.97) | 17.49 (106.73) | 4.10 (1.47) | 17.17 (8.13) |
| Intestinal ischaemia (PT: 10022680) | 7 | 8.32 (3.95-17.51) | 8.32 (44.64) | 3.04 (1.09) | 8.25 (3.92) |
| Pancreatic disorder (PT: 10033616) | 6 | 6.76 (3.03-15.10) | 6.76 (29.23) | 2.75 (0.79) | 6.72 (3.01) |
| Volvulus (PT: 10047697) | 5 | 10.34 (4.28-24.96) | 10.33 (41.67) | 3.35 (0.82) | 10.23 (4.23) |
| Oesophageal rupture (PT: 10052211) | 5 | 37.38 (15.28-91.47) | 37.37 (169.90) | 5.17 (1.19) | 35.91 (14.68) |
| Pancreatic cyst (PT: 10033615) | 4 | 12.42 (4.63-33.31) | 12.41 (41.40) | 3.62 (0.61) | 12.26 (4.57) |
| Large intestinal obstruction (PT: 10062062) | 4 | 8.65 (3.23-23.15) | 8.64 (26.78) | 3.10 (0.47) | 8.57 (3.20) |
| Obstructive pancreatitis (PT: 10079822) | 4 | 20.16 (7.48-54.31) | 20.15 (71.21) | 4.30 (0.75) | 19.73 (7.32) |
| Pneumatosis intestinalis (PT: 10057030) | 4 | 6.06 (2.27-16.21) | 6.06 (16.79) | 2.59 (0.29) | 6.03 (2.25) |
| Pancreatic failure (PT: 10079281) | 4 | 7.77 (2.90-20.79) | 7.76 (23.37) | 2.95 (0.42) | 7.71 (2.88) |
| Obstruction gastric (PT: 10029957) | 4 | 17.50 (6.51-47.09) | 17.50 (61.04) | 4.10 (0.71) | 17.18 (6.39) |
| Gastritis haemorrhagic (PT: 10017866) | 3 | 20.39 (6.49-64.04) | 20.38 (54.07) | 4.32 (0.33) | 19.95 (6.35) |
| **Metabolism and nutrition disorders (SOC: 10027433)** | | | | | |
| Dehydration (PT: 10012174) | 266 | 12.73 (11.27-14.39) | 12.49 (2777.71) | 3.62 (3.38) | 12.33 (10.91) |
| Diabetic ketoacidosis (PT: 10012671) | 59 | 16.18 (12.50-20.95) | 16.11 (821.78) | 3.99 (3.29) | 15.85 (12.24) |
| Feeding disorder (PT: 10061148) | 55 | 10.55 (8.09-13.78) | 10.51 (468.14) | 3.38 (2.77) | 10.40 (7.97) |
| Hypophagia (PT: 10063743) | 38 | 8.07 (5.86-11.12) | 8.05 (232.73) | 3.00 (2.30) | 7.99 (5.80) |
| Ketoacidosis (PT: 10023379) | 24 | 21.43 (14.29-32.14) | 21.40 (455.79) | 4.39 (2.96) | 20.92 (13.95) |
| Fluid intake reduced (PT: 10056291) | 20 | 20.27 (13.01-31.59) | 20.24 (357.83) | 4.31 (2.75) | 19.82 (12.72) |
| Euglycaemic diabetic ketoacidosis (PT: 10080061) | 19 | 15.87 (10.08-24.99) | 15.85 (259.80) | 3.96 (2.52) | 15.59 (9.90) |
| Malnutrition (PT: 10061273) | 16 | 8.08 (4.94-13.23) | 8.08 (98.31) | 3.00 (1.80) | 8.01 (4.90) |
| Electrolyte imbalance (PT: 10014418) | 15 | 6.54 (3.93-10.87) | 6.53 (69.80) | 2.70 (1.55) | 6.49 (3.91) |
| Hypovolaemia (PT: 10021137) | 9 | 10.64 (5.51-20.54) | 10.63 (77.64) | 3.40 (1.51) | 10.52 (5.45) |
| Acidosis (PT: 10000486) | 8 | 7.24 (3.61-14.51) | 7.23 (42.63) | 2.84 (1.12) | 7.18 (3.58) |
| Starvation ketoacidosis (PT: 10082528) | 6 | 158.34 (66.46-377.22) | 158.27 (796.99) | 7.07 (1.56) | 134.68 (56.53) |
| Marasmus (PT: 10026820) | 3 | 7.20 (2.31-22.41) | 7.19 (15.87) | 2.84 (0.04) | 7.14 (2.29) |
| Ketosis (PT: 10023391) | 3 | 13.06 (4.18-40.84) | 13.06 (32.93) | 3.69 (0.24) | 12.89 (4.12) |
| **Hepatobiliary disorders (SOC: 10019805)** | | | | | |
| Cholelithiasis (PT: 10008629) | 78 | 17.00 (13.58-21.29) | 16.91 (1146.04) | 4.05 (3.46) | 16.61 (13.27) |
| Gallbladder disorder (PT: 10017626) | 48 | 20.89 (15.68-27.83) | 20.82 (885.11) | 4.35 (3.45) | 20.37 (15.29) |
| Cholecystitis (PT: 10008612) | 42 | 24.78 (18.23-33.68) | 24.70 (929.62) | 4.59 (3.52) | 24.06 (17.70) |
| Hepatitis (PT: 10019717) | 17 | 3.86 (2.40-6.22) | 3.86 (35.83) | 1.94 (1.05) | 3.84 (2.39) |
| Hepatic cirrhosis (PT: 10019641) | 16 | 4.51 (2.76-7.37) | 4.50 (43.37) | 2.16 (1.19) | 4.48 (2.74) |
| Cholecystitis acute (PT: 10008614) | 16 | 20.32 (12.38-33.36) | 20.30 (287.05) | 4.31 (2.53) | 19.87 (12.10) |
| Jaundice (PT: 10023126) | 12 | 3.91 (2.22-6.90) | 3.91 (25.91) | 1.96 (0.87) | 3.90 (2.21) |
| Biliary colic (PT: 10004663) | 10 | 18.32 (9.79-34.27) | 18.30 (160.31) | 4.17 (1.94) | 17.96 (9.60) |
| Cholangitis acute (PT: 10008605) | 7 | 45.52 (21.30-97.28) | 45.49 (289.90) | 5.44 (1.73) | 43.34 (20.28) |
| Gallbladder enlargement (PT: 10062693) | 4 | 19.40 (7.20-52.24) | 19.39 (68.30) | 4.25 (0.74) | 19.00 (7.06) |
| Portal vein thrombosis (PT: 10036206) | 4 | 8.16 (3.05-21.83) | 8.15 (24.88) | 3.02 (0.44) | 8.09 (3.02) |
| Acute cholecystitis necrotic (PT: 10090325) | 3 | 49.84 (15.58-159.41) | 49.82 (135.98) | 5.56 (0.42) | 47.25 (14.77) |
| **Nervous system disorders (SOC: 10029205)** | | | | | |
| Loss of consciousness (PT: 10024855) | 79 | 4.01 (3.21-5.00) | 3.99 (176.33) | 1.99 (1.61) | 3.97 (3.18) |
| Cerebrovascular accident (PT: 10008190) | 69 | 3.07 (2.42-3.89) | 3.06 (95.37) | 1.61 (1.22) | 3.05 (2.41) |
| Syncope (PT: 10042772) | 62 | 3.79 (2.95-4.87) | 3.78 (126.25) | 1.91 (1.49) | 3.77 (2.93) |
| Hypoglycaemic unconsciousness (PT: 10065981) | 11 | 76.54 (41.34-141.70) | 76.47 (754.99) | 6.14 (2.51) | 70.54 (38.11) |
| Allodynia (PT: 10053552) | 9 | 28.64 (14.75-55.63) | 28.62 (232.50) | 4.80 (1.99) | 27.77 (14.30) |
| Wernicke's encephalopathy (PT: 10047911) | 6 | 60.49 (26.46-138.29) | 60.46 (328.71) | 5.83 (1.53) | 56.71 (24.80) |
| **Renal and urinary disorders (SOC: 10038359)** | | | | | |
| Acute kidney injury (PT: 10069339) | 121 | 3.51 (2.93-4.19) | 3.48 (213.83) | 1.80 (1.50) | 3.47 (2.90) |
| Renal failure (PT: 10038435) | 65 | 3.37 (2.64-4.31) | 3.36 (107.57) | 1.75 (1.34) | 3.35 (2.63) |
| Nephrolithiasis (PT: 10029148) | 30 | 2.94 (2.06-4.21) | 2.94 (38.27) | 1.55 (0.94) | 2.93 (2.05) |
| Hydronephrosis (PT: 10020524) | 6 | 4.51 (2.02-10.07) | 4.51 (16.31) | 2.17 (0.49) | 4.49 (2.01) |
| Prerenal failure (PT: 10072370) | 3 | 11.21 (3.59-35.02) | 11.21 (27.56) | 3.47 (0.20) | 11.08 (3.55) |
| **Psychiatric disorders (SOC: 10037175)** | | | | | |
| Suicidal ideation (PT: 10042458) | 78 | 5.66 (4.53-7.08) | 5.63 (295.57) | 2.49 (2.08) | 5.60 (4.48) |
| Depressed mood (PT: 10012374) | 69 | 6.81 (5.37-8.64) | 6.78 (337.72) | 2.75 (2.29) | 6.74 (5.31) |
| Panic attack (PT: 10033664) | 37 | 6.33 (4.58-8.75) | 6.31 (164.37) | 2.65 (1.99) | 6.28 (4.54) |
| Self-injurious ideation (PT: 10051154) | 6 | 8.64 (3.87-19.31) | 8.64 (40.13) | 3.10 (0.94) | 8.56 (3.83) |
| Depression suicidal (PT: 10012397) | 5 | 7.37 (3.05-17.76) | 7.36 (27.27) | 2.87 (0.65) | 7.31 (3.03) |
| Obsessive-compulsive disorder (PT: 10029898) | 5 | 5.14 (2.14-12.39) | 5.14 (16.59) | 2.36 (0.42) | 5.12 (2.12) |
| **Investigations (SOC: 10022891)** | | | | | |
| Blood potassium decreased (PT: 10005724) | 25 | 4.63 (3.12-6.85) | 4.62 (70.53) | 2.20 (1.45) | 4.60 (3.10) |
| Lipase increased (PT: 10024574) | 22 | 19.74 (12.94-30.13) | 19.71 (382.38) | 4.27 (2.82) | 19.31 (12.65) |
| Glomerular filtration rate decreased (PT: 10018358) | 17 | 5.41 (3.36-8.72) | 5.41 (60.73) | 2.43 (1.43) | 5.38 (3.34) |
| Blood sodium decreased (PT: 10005802) | 12 | 3.60 (2.04-6.35) | 3.60 (22.42) | 1.84 (0.78) | 3.59 (2.03) |
| Pancreatic enzymes increased (PT: 10061900) | 8 | 31.91 (15.76-64.60) | 31.89 (231.14) | 4.95 (1.86) | 30.83 (15.23) |
| Red blood cell count increased (PT: 10038155) | 4 | 6.04 (2.26-16.15) | 6.04 (16.71) | 2.59 (0.29) | 6.01 (2.25) |
| Amylase increased (PT: 10002016) | 4 | 5.51 (2.06-14.73) | 5.51 (14.68) | 2.45 (0.24) | 5.48 (2.05) |
| Blood electrolytes decreased (PT: 10061715) | 3 | 10.00 (3.21-31.22) | 10.00 (24.04) | 3.31 (0.16) | 9.90 (3.17) |
| **Immune system disorders (SOC: 10021428)** | | | | | |
| Anaphylactic reaction (PT: 10002198) | 50 | 4.41 (3.34-5.83) | 4.40 (130.70) | 2.13 (1.63) | 4.38 (3.32) |
| Anaphylactic shock (PT: 10002199) | 20 | 4.01 (2.59-6.23) | 4.01 (44.99) | 2.00 (1.17) | 4.00 (2.57) |
| **Infections and infestations (SOC: 10021881)** | | | | | |
| Appendicitis (PT: 10003011) | 25 | 12.42 (8.37-18.44) | 12.40 (258.51) | 3.61 (2.52) | 12.25 (8.25) |
| Gastroenteritis viral (PT: 10017918) | 14 | 3.40 (2.01-5.74) | 3.40 (23.58) | 1.76 (0.80) | 3.39 (2.00) |
| Gastroenteritis (PT: 10017888) | 11 | 4.38 (2.42-7.93) | 4.38 (28.54) | 2.13 (0.93) | 4.36 (2.41) |
| Appendicitis perforated (PT: 10003012) | 7 | 15.66 (7.42-33.08) | 15.66 (94.39) | 3.95 (1.43) | 15.40 (7.29) |
| Cholecystitis infective (PT: 10062631) | 6 | 10.70 (4.78-23.94) | 10.70 (52.13) | 3.40 (1.06) | 10.58 (4.73) |
| Intestinal sepsis (PT: 10075622) | 3 | 35.88 (11.31-113.79) | 35.87 (97.79) | 5.11 (0.40) | 34.53 (10.89) |
| **Reproductive system and breast disorders (SOC: 10038604)** | | | | | |
| Vaginal haemorrhage (PT: 10046910) | 18 | 4.26 (2.68-6.76) | 4.25 (44.57) | 2.08 (1.19) | 4.24 (2.67) |
| Heavy menstrual bleeding (PT: 10085423) | 13 | 3.57 (2.07-6.15) | 3.56 (23.90) | 1.83 (0.81) | 3.55 (2.06) |
| Menstruation delayed (PT: 10027336) | 10 | 9.45 (5.07-17.62) | 9.44 (74.68) | 3.23 (1.53) | 9.35 (5.01) |
| Postmenopausal haemorrhage (PT: 10055870) | 7 | 14.64 (6.94-30.90) | 14.63 (87.49) | 3.85 (1.40) | 14.41 (6.83) |
| **Skin and subcutaneous tissue disorders (SOC: 10040785)** | | | | | |
| Angioedema (PT: 10002424) | 25 | 3.24 (2.19-4.81) | 3.24 (38.60) | 1.69 (1.01) | 3.23 (2.18) |
| Cold sweat (PT: 10009866) | 12 | 4.37 (2.48-7.71) | 4.37 (31.04) | 2.12 (0.99) | 4.35 (2.47) |
| **Eye disorders (SOC: 10015919)** | | | | | |
| Retinal detachment (PT: 10038848) | 9 | 5.26 (2.73-10.14) | 5.26 (30.89) | 2.39 (0.96) | 5.24 (2.72) |
| Retinal haemorrhage (PT: 10038867) | 5 | 5.02 (2.09-12.10) | 5.02 (16.01) | 2.32 (0.40) | 5.00 (2.08) |
| Vitreous haemorrhage (PT: 10047655) | 4 | 8.61 (3.21-23.04) | 8.60 (26.62) | 3.09 (0.47) | 8.53 (3.19) |
| Optic ischaemic neuropathy (PT: 10030924) | 3 | 7.16 (2.30-22.29) | 7.16 (15.76) | 2.83 (0.04) | 7.11 (2.28) |
| **Pregnancy, puerperium and perinatal conditions (SOC: 10036585)** | | | | | |
| Abortion spontaneous (PT: 10000234) | 19 | 3.29 (2.10-5.17) | 3.29 (30.19) | 1.71 (0.91) | 3.28 (2.09) |
| **Endocrine disorders (SOC: 10014698)** | | | | | |
| Thyroid mass (PT: 10058900) | 14 | 14.75 (8.70-25.02) | 14.74 (176.38) | 3.86 (2.18) | 14.51 (8.56) |
| Goitre (PT: 10018498) | 5 | 8.83 (3.66-21.31) | 8.83 (34.37) | 3.13 (0.75) | 8.75 (3.63) |
| **Musculoskeletal and connective tissue disorders (SOC: 10028395)** | | | | | |
| Neck mass (PT: 10049146) | 7 | 13.09 (6.20-27.60) | 13.08 (76.97) | 3.69 (1.34) | 12.91 (6.12) |
| **Blood and lymphatic system disorders (SOC: 10005329)** | | | | | |
| Lymph node pain (PT: 10025182) | 4 | 11.21 (4.18-30.07) | 11.21 (36.74) | 3.47 (0.58) | 11.08 (4.13) |
| Thrombotic thrombocytopenic purpura (PT: 10043648) | 3 | 9.38 (3.01-29.25) | 9.37 (22.21) | 3.22 (0.14) | 9.29 (2.98) |
| **Ear and labyrinth disorders (SOC: 10013993)** | | | | | |
| Deafness unilateral (PT: 10048812) | 5 | 5.37 (2.23-12.93) | 5.36 (17.65) | 2.42 (0.45) | 5.34 (2.22) |
| **General disorders and administration site conditions (SOC: 10018065)** | | | | | |
| Injection site hypersensitivity (PT: 10022071) | 4 | 5.52 (2.07-14.76) | 5.52 (14.71) | 2.46 (0.24) | 5.49 (2.05) |
| **Respiratory, thoracic and mediastinal disorders (SOC: 10038738)** | | | | | |
| Pneumomediastinum (PT: 10050184) | 4 | 8.97 (3.35-24.02) | 8.97 (28.04) | 3.15 (0.49) | 8.89 (3.32) |

For tirzepatide, only the reports with serious outcomes were included. **Abbreviations:** PT, preferred term; ROR, reporting odds ratio; CI, confidence interval; PRR, proportional reporting ratio; χ2, chi-squared; IC, information component; IC025, lower limit of 95% confidence interval of IC; EBGM, empirical Bayesian geometric mean; EBGM05, lower limit of 95% confidence interval of EBGM.
